# Supplementary material for: (S)-N-Benzyl-1-phenyl-3,4-dihydroisoqunoline-2(1H)-carboxamide Derivatives, Multi-Target Inhibitors of Monoamine Oxidase and Cholinesterase: Design, Synthesis, and Biological Activity
Source: Molecules. 2023 Feb 9;28(4):1654. doi: 10.3390/molecules28041654 (PMC9967051; doi:10.3390/molecules28041654)
Supplement: Supplementary file 1 [file molecules-28-01654-s001.zip › molecules-2167235-supplementary.pdf]

10072021
G-1 cdcl3 2021-10-7

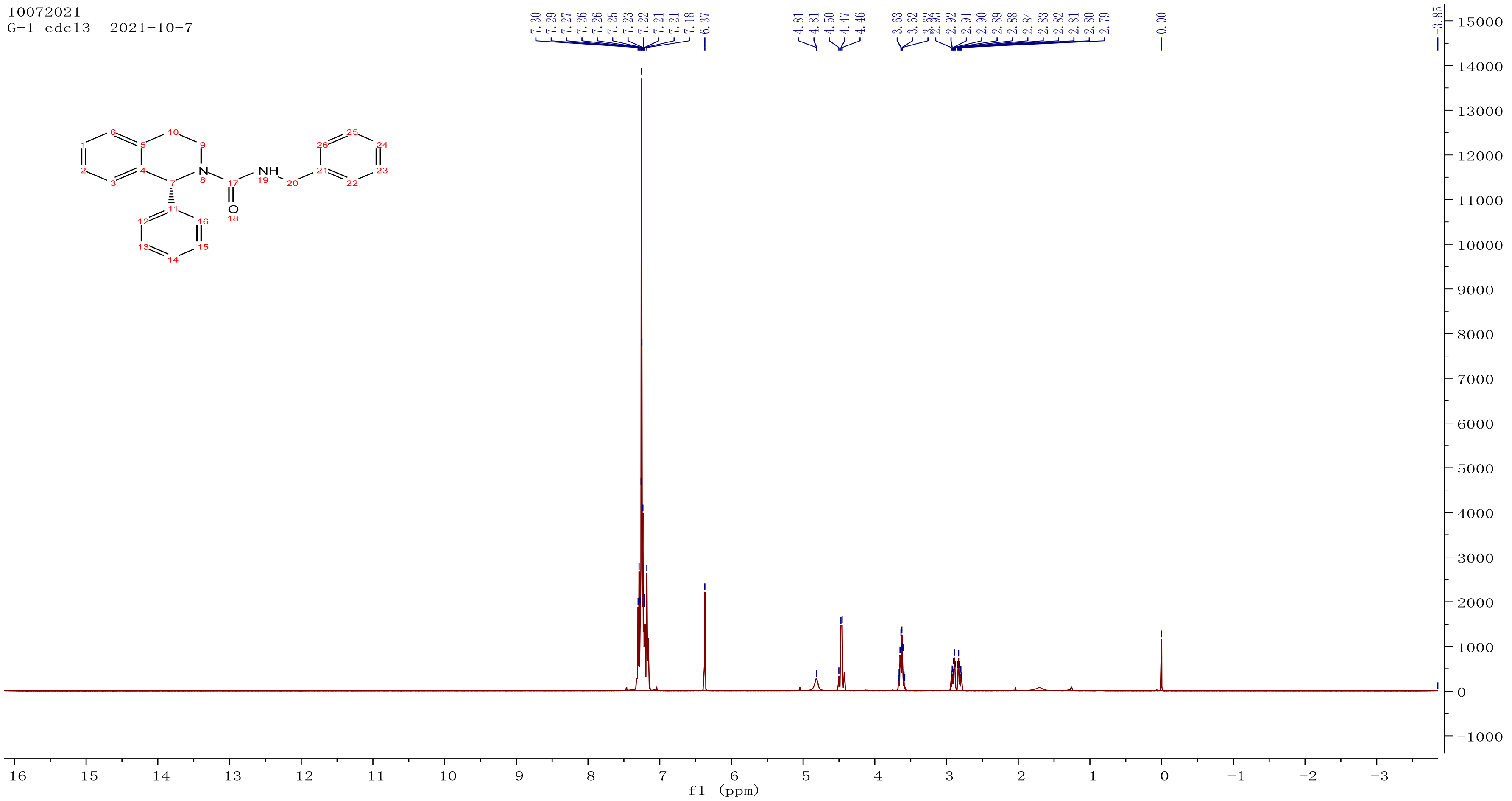

^{1}H-NMR Spectral of **2a**

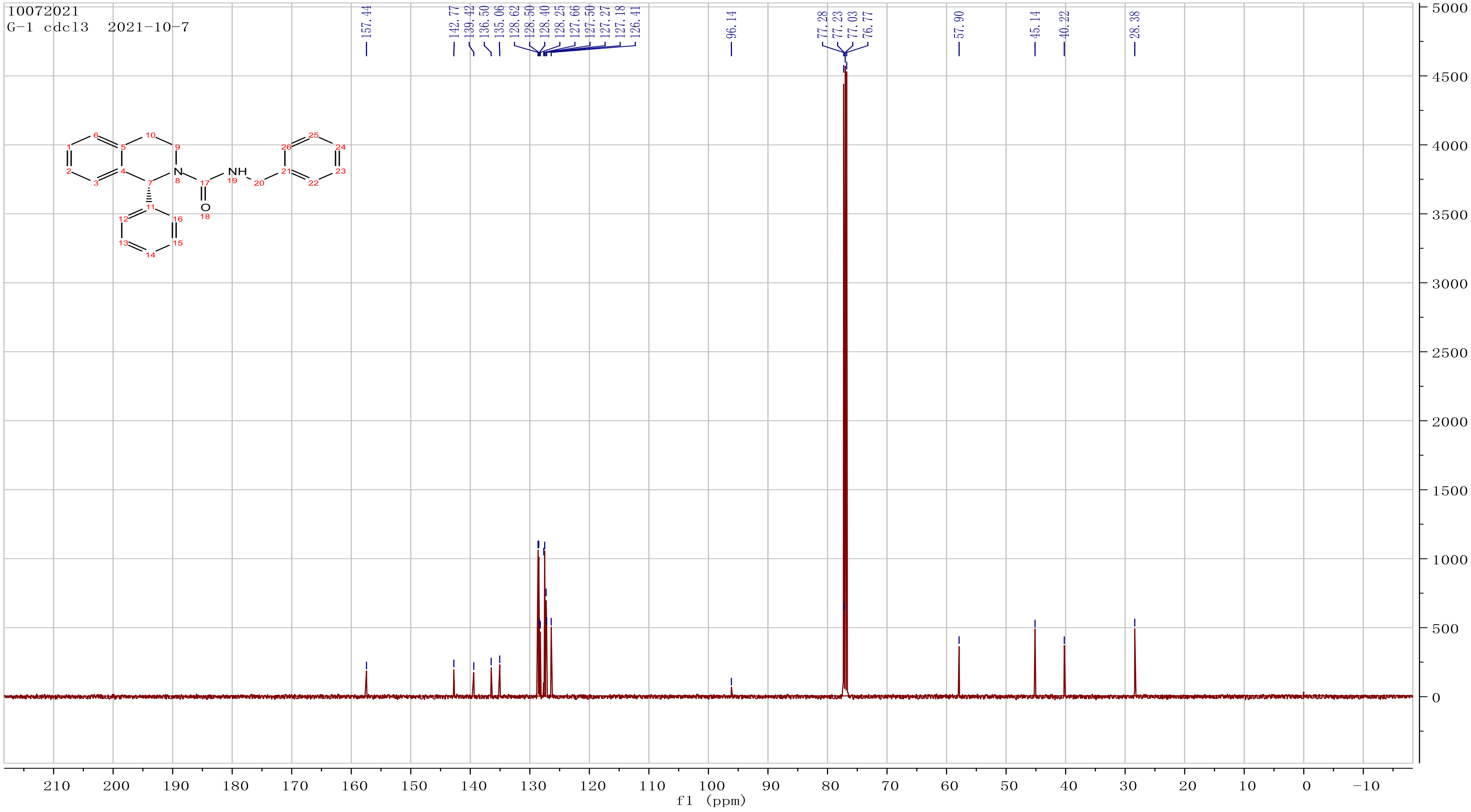

^{13}C-NMR Spectral of **2a**

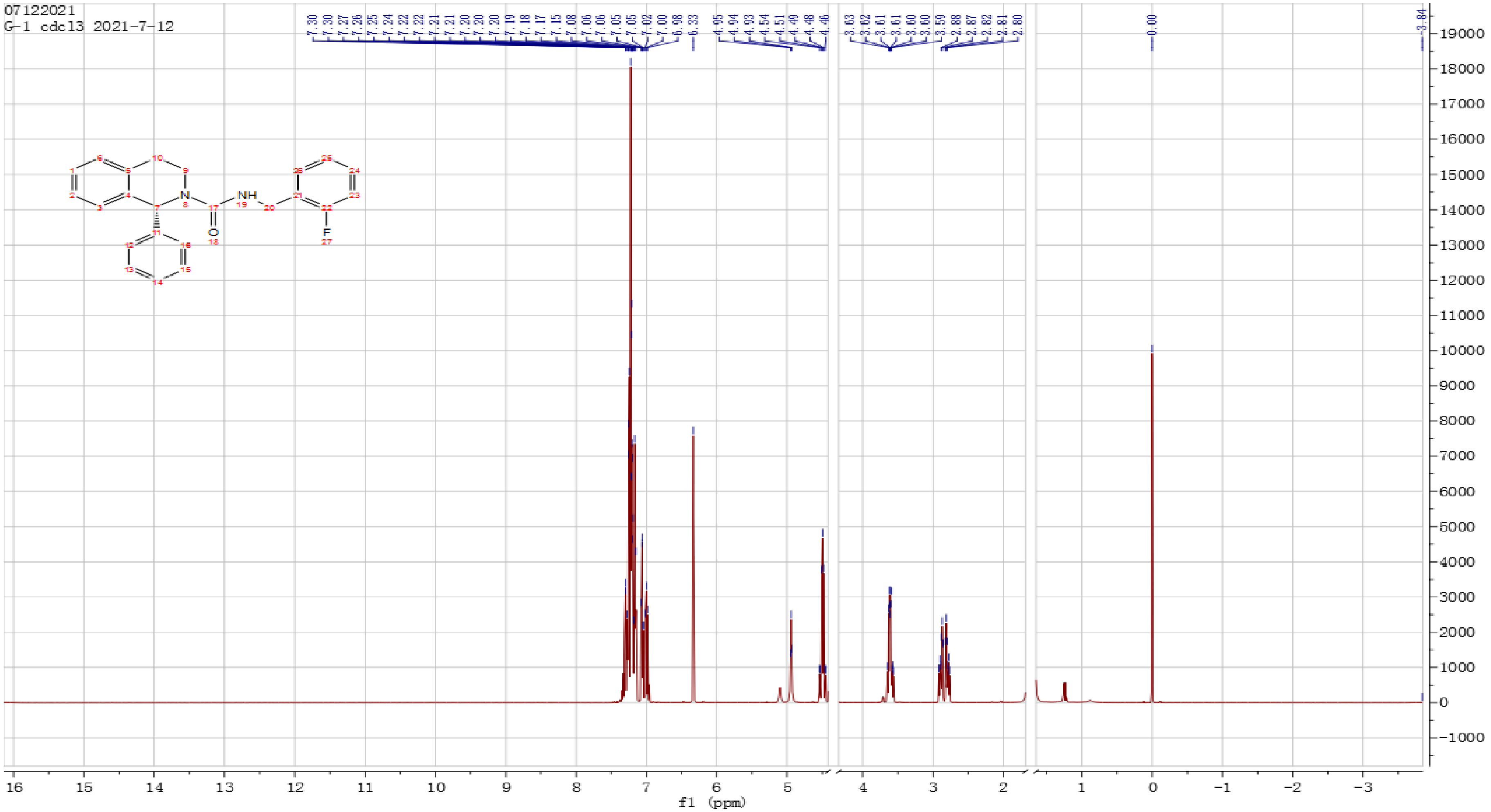

^{1}H-NMR Spectral of **2b**

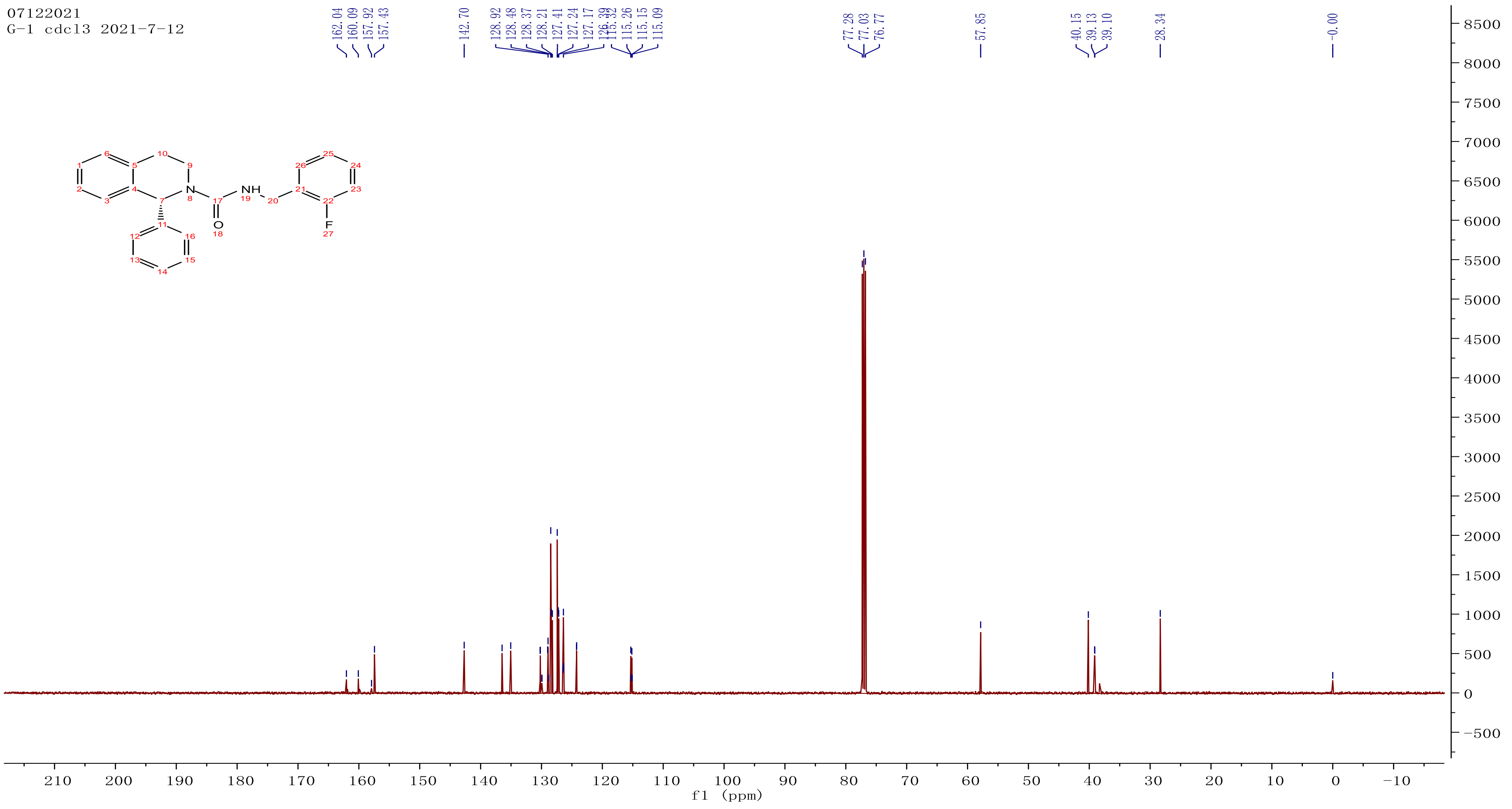

^{13}C-NMR Spectral of **2b**

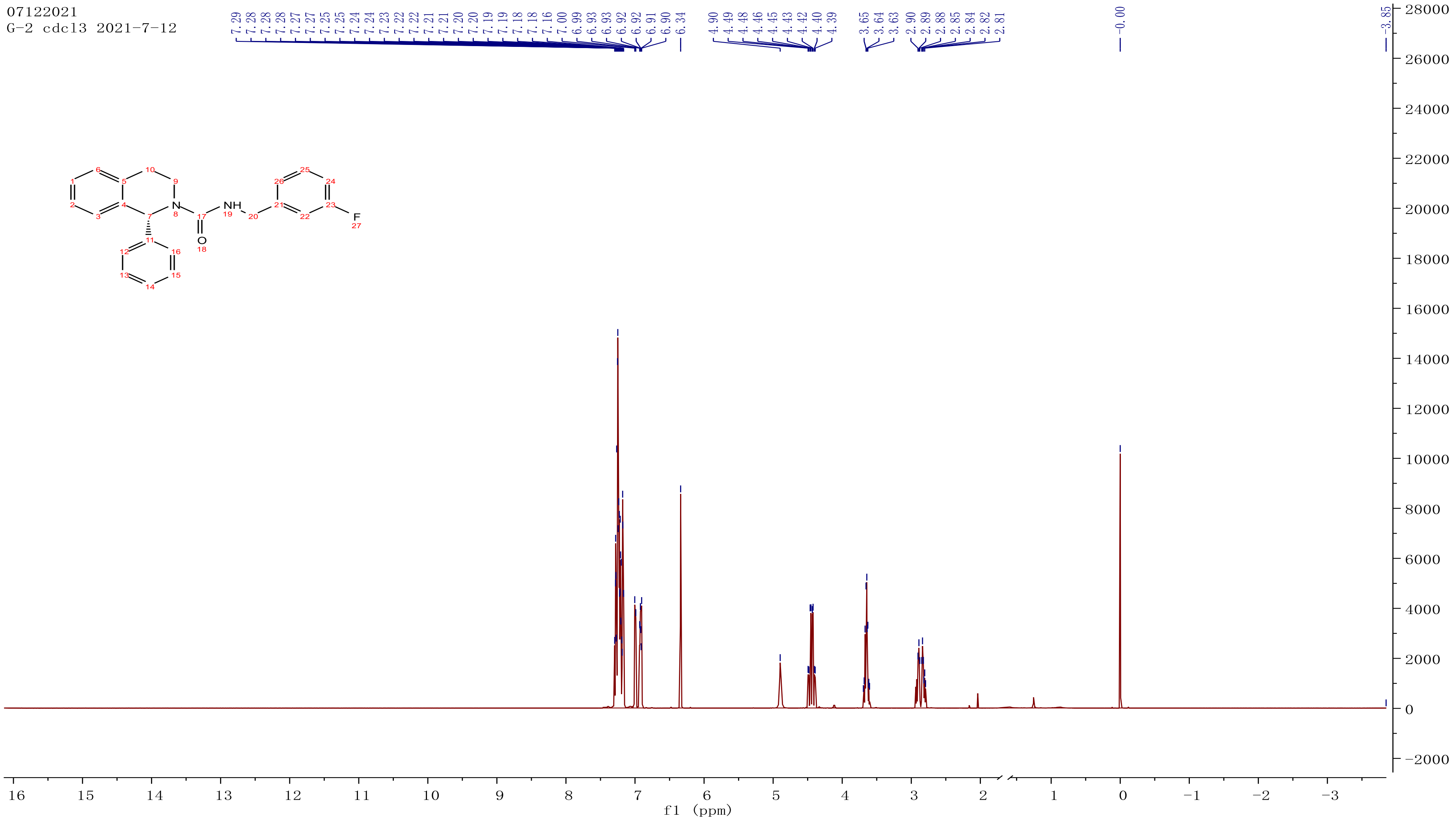

^{1}H-NMR Spectral of **2c**

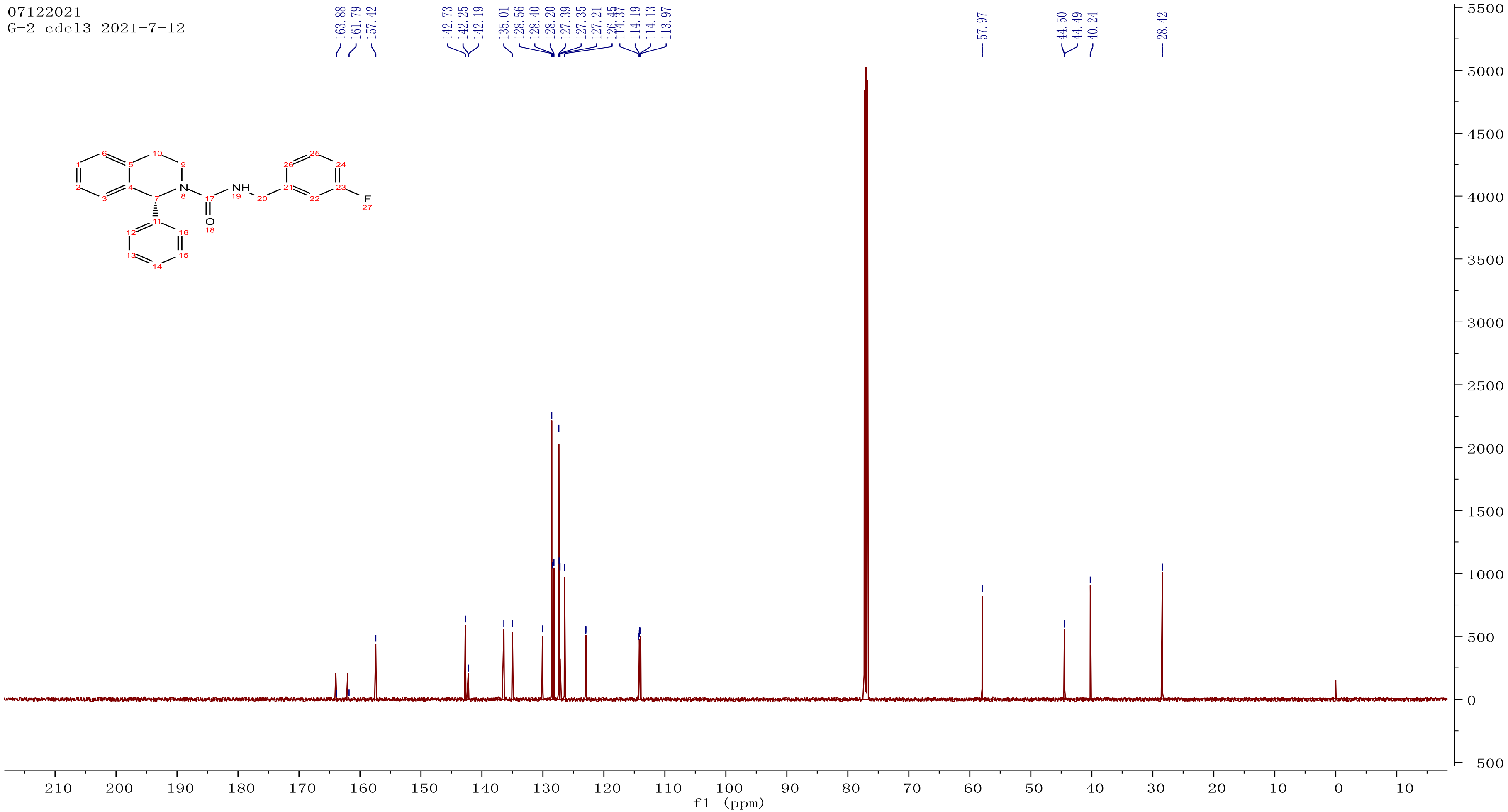

^{13}C-NMR Spectral of **2c**

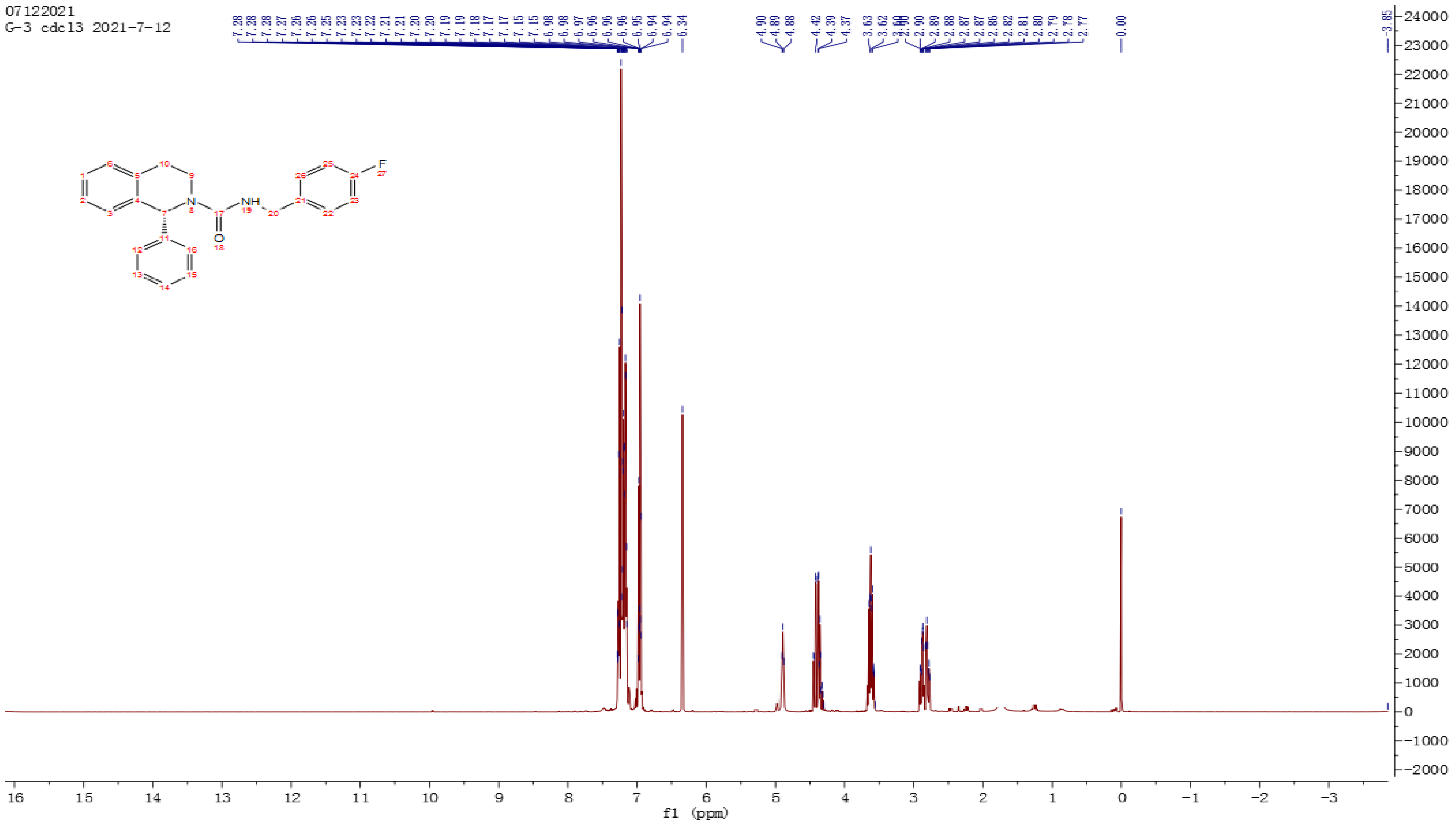

^{1}H-NMR Spectral of **2d**

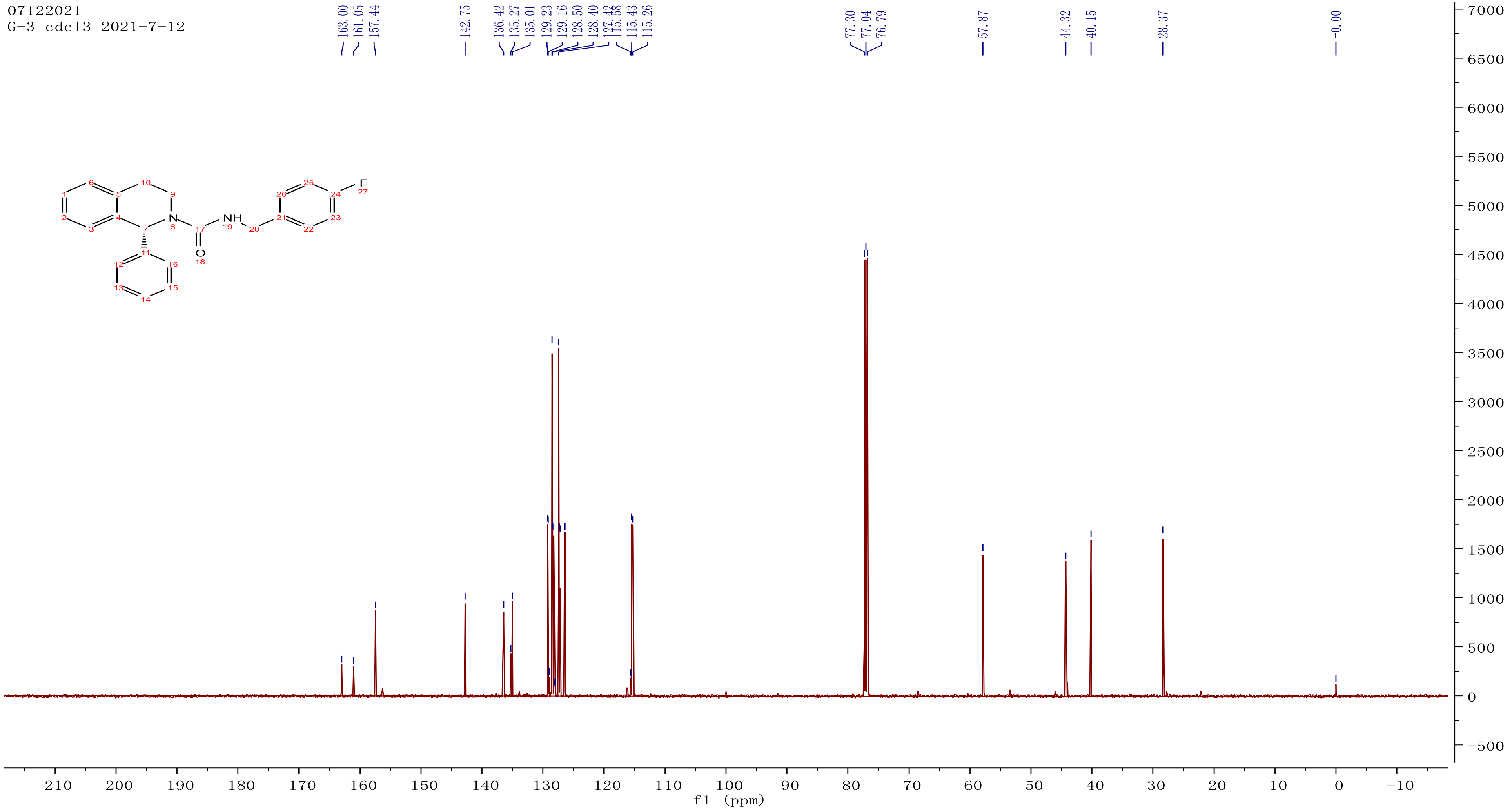

^{13}C-NMR Spectral of **2d**

10072021
G-2 cdcl3 2021-10-7

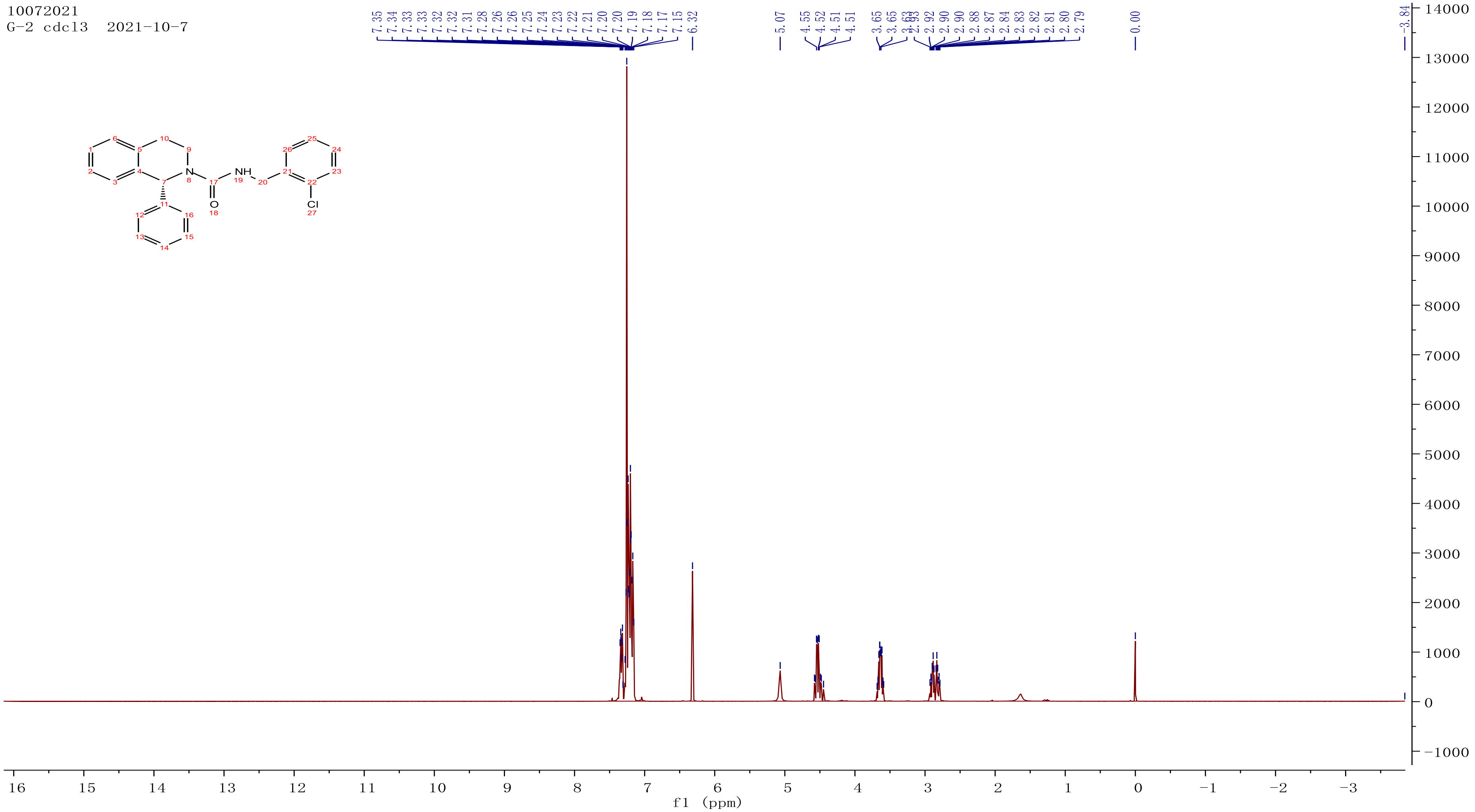

^{1}H-NMR Spectral of **2e**

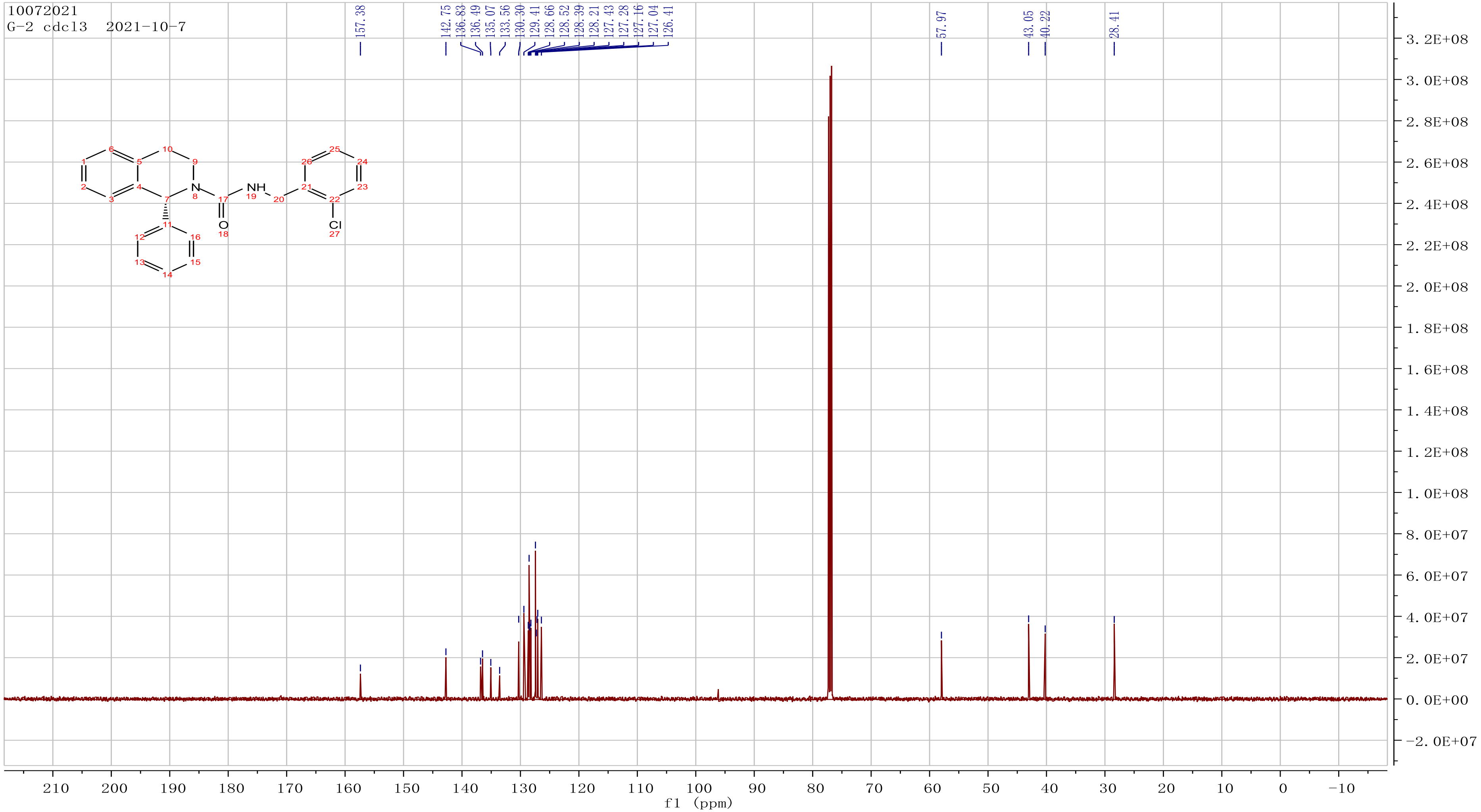

^{13}C-NMR Spectral of **2e**

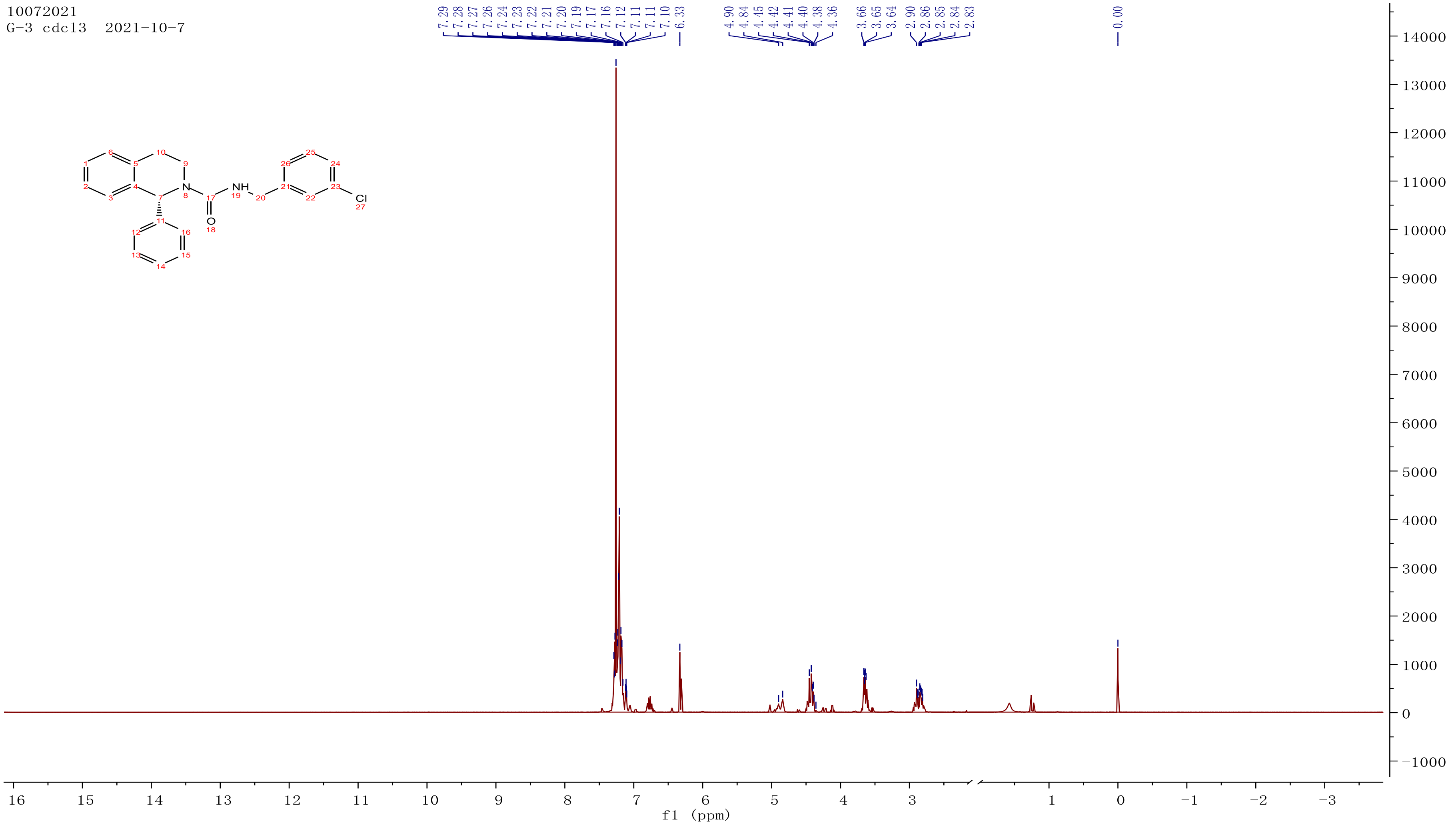

^{1}H-NMR Spectral of **2f**

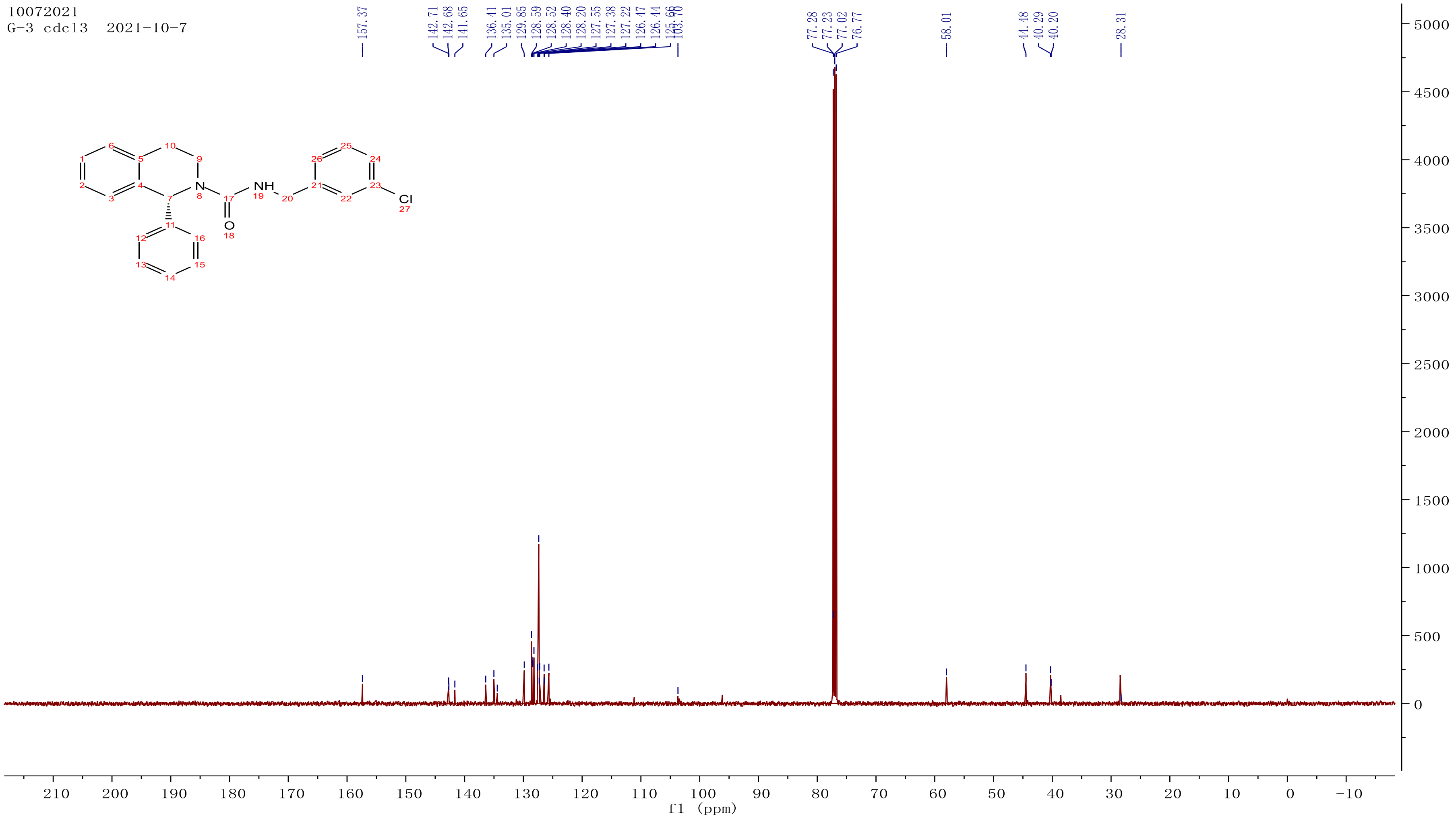

^{13}C-NMR Spectral of **2f**

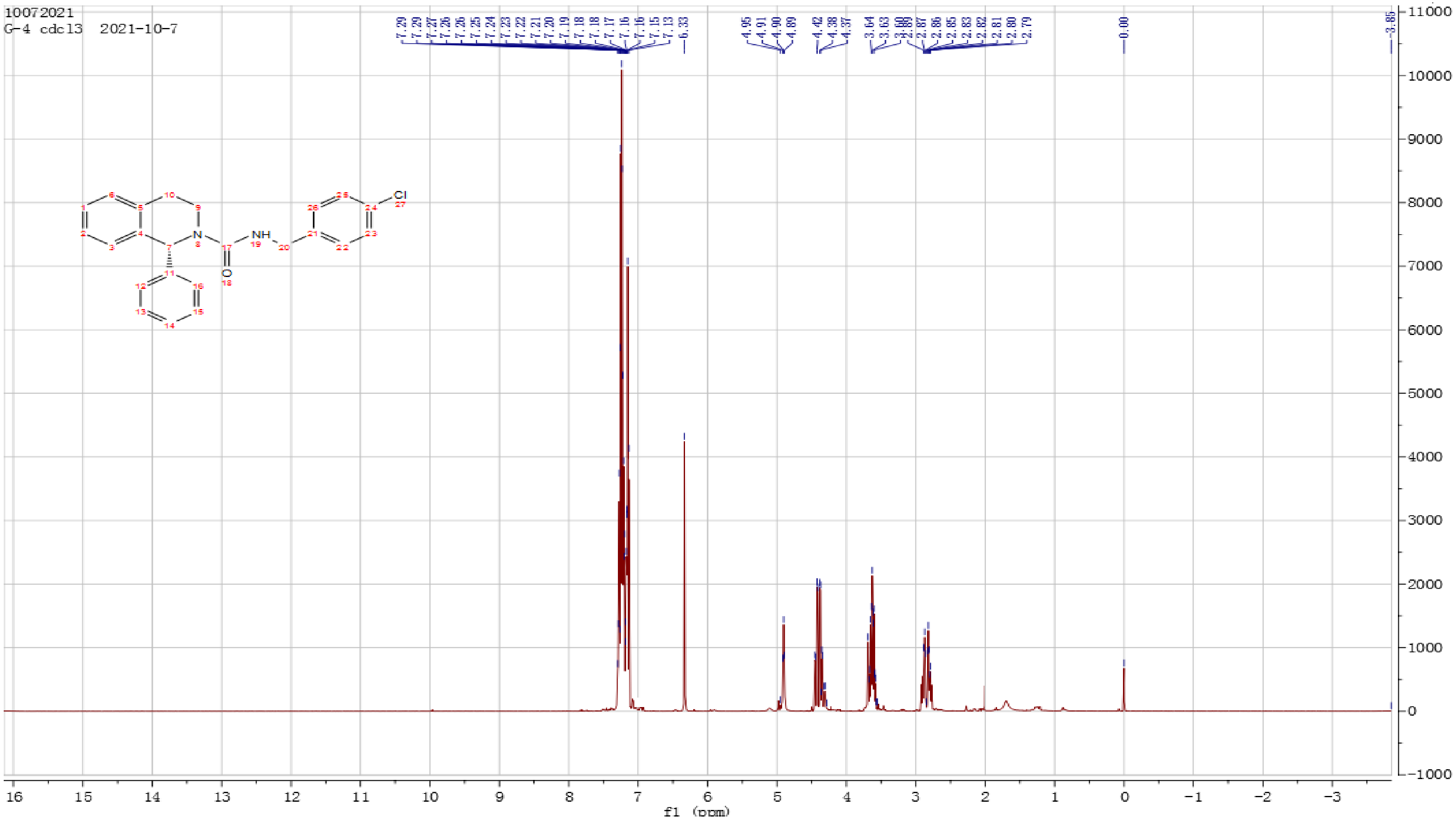

^{1}H-NMR Spectral of **2g**

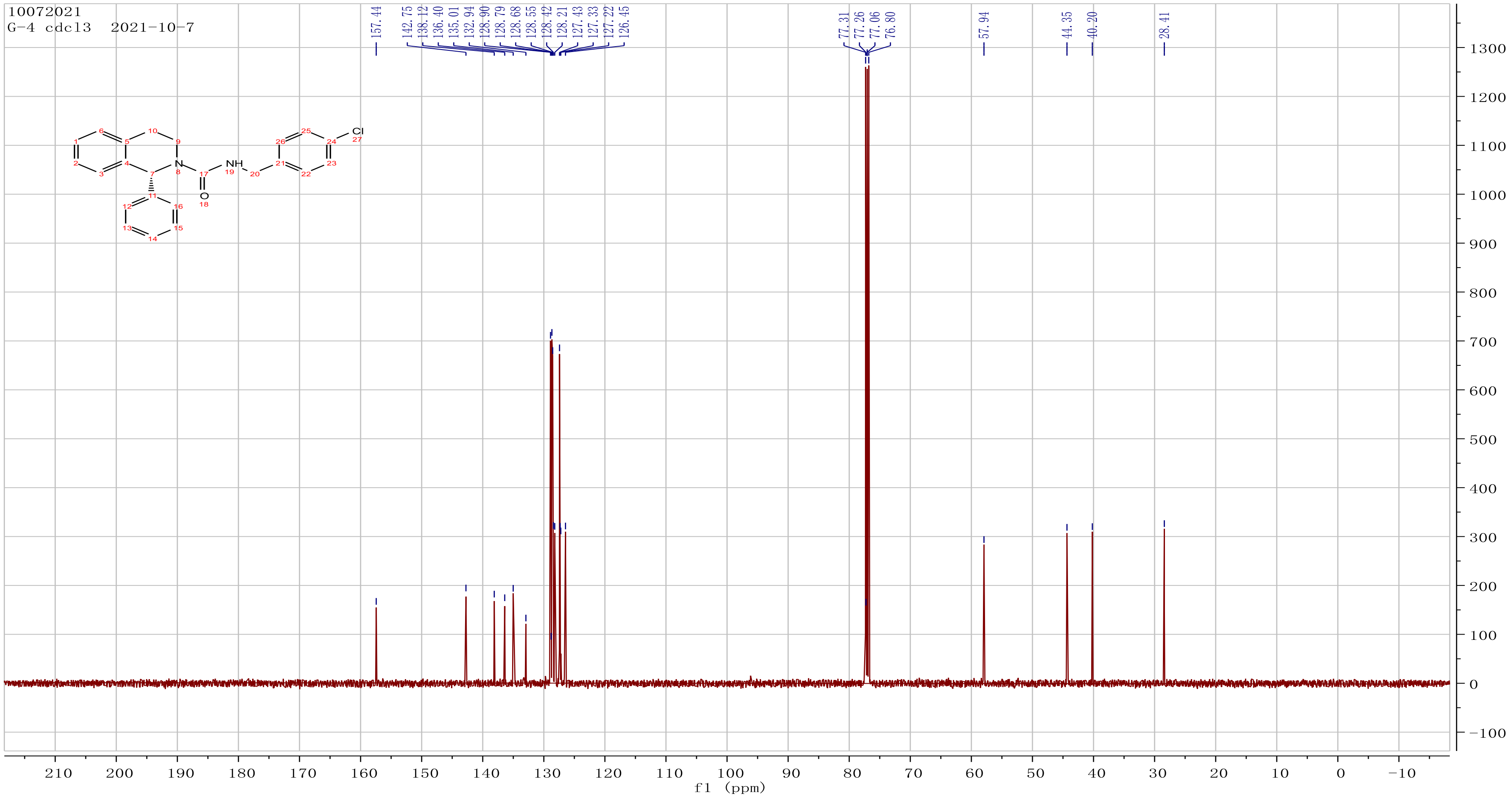

^{13}C-NMR Spectral of **2g**

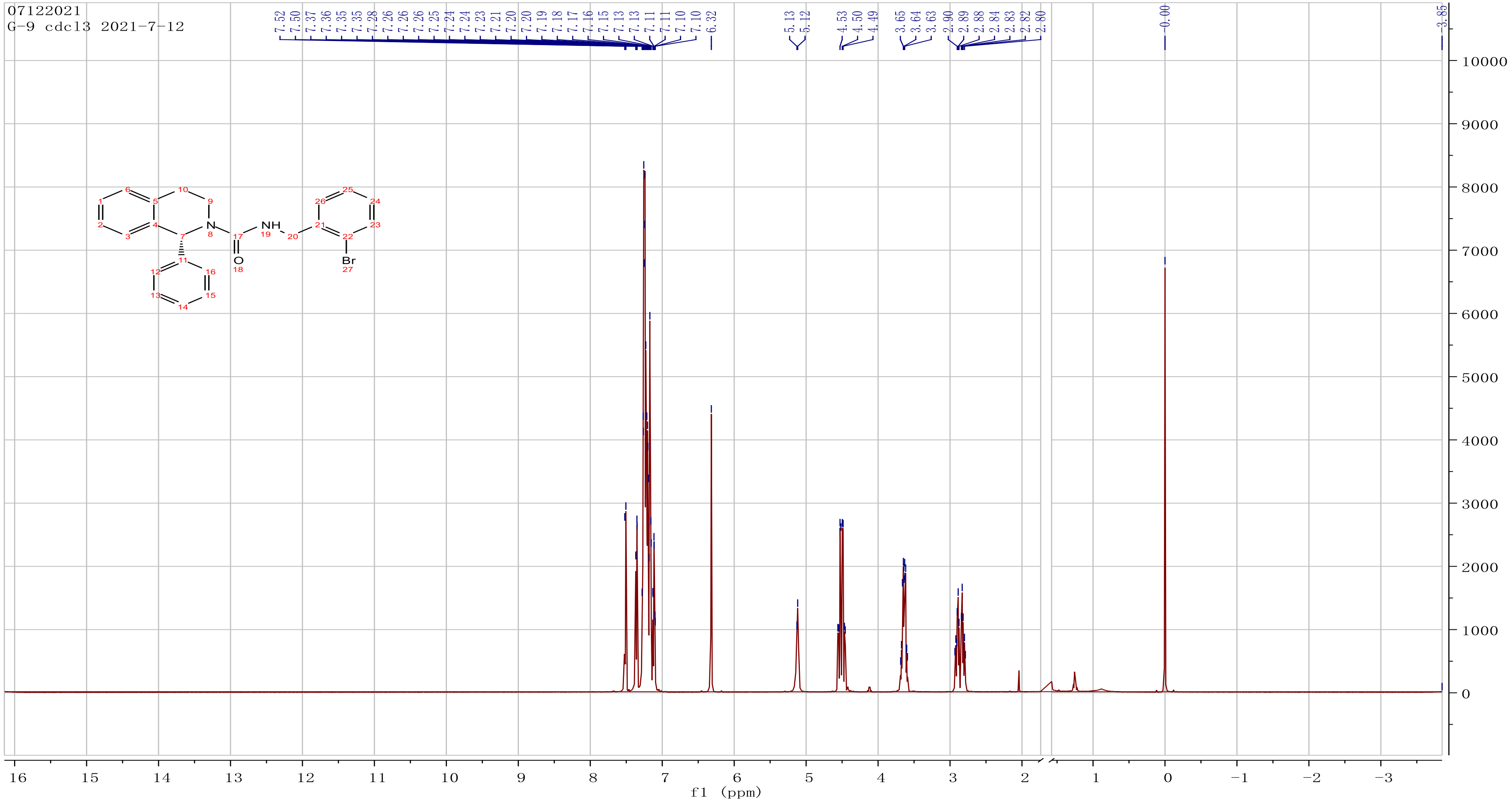

^{1}H-NMR Spectral of **2h**

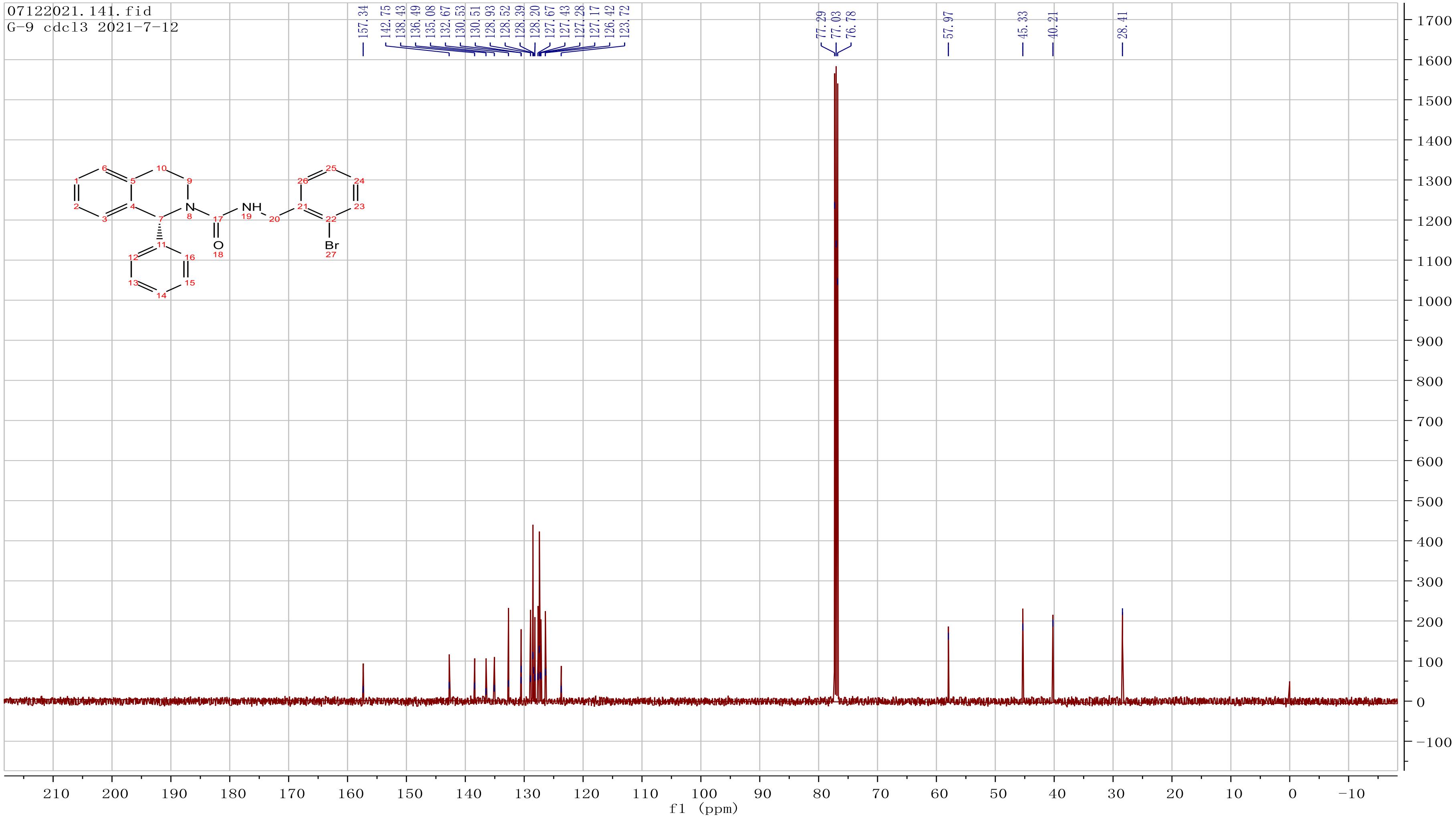

^{13}C-NMR Spectral of **2h**

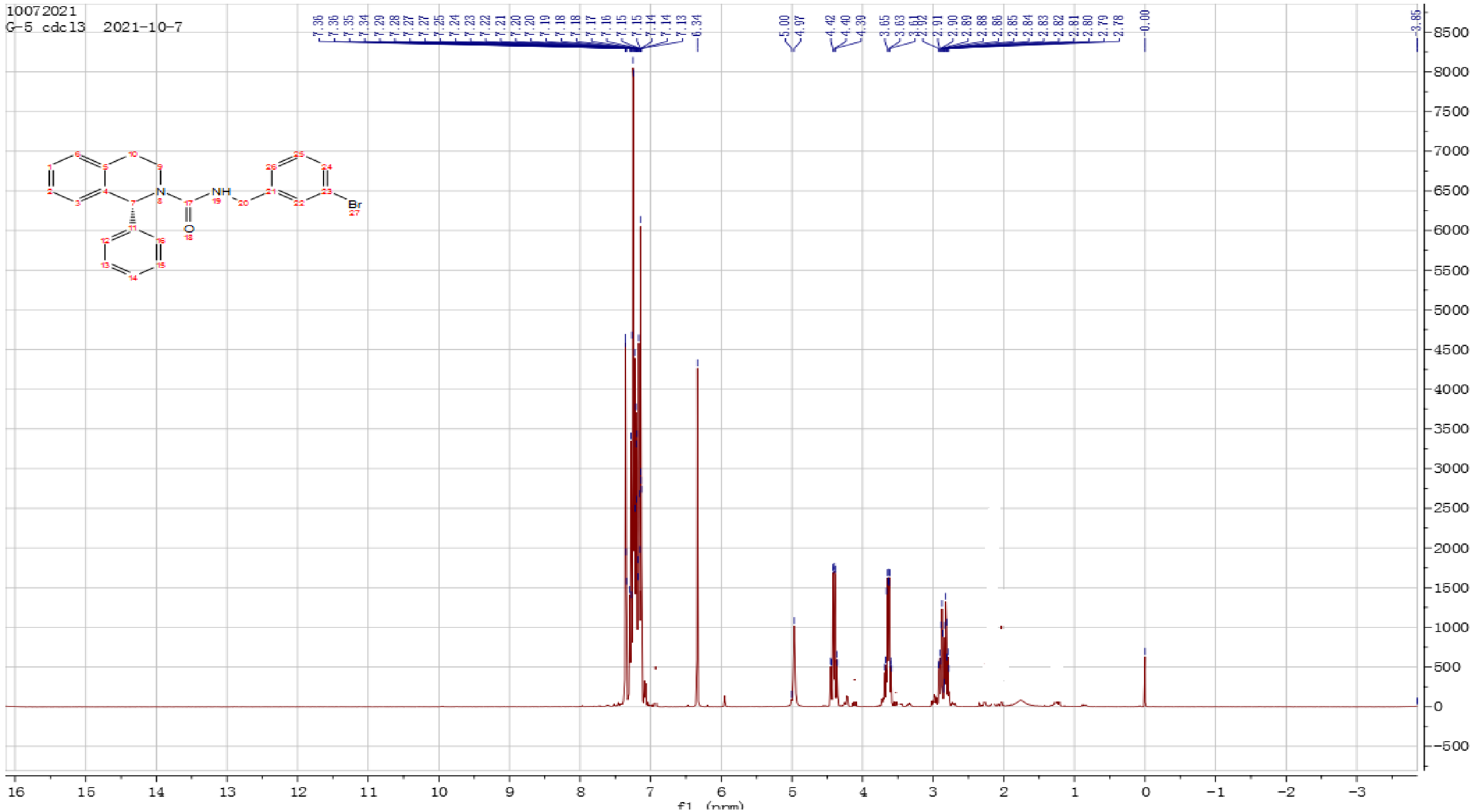

^{1}H-NMR Spectral of **2i**

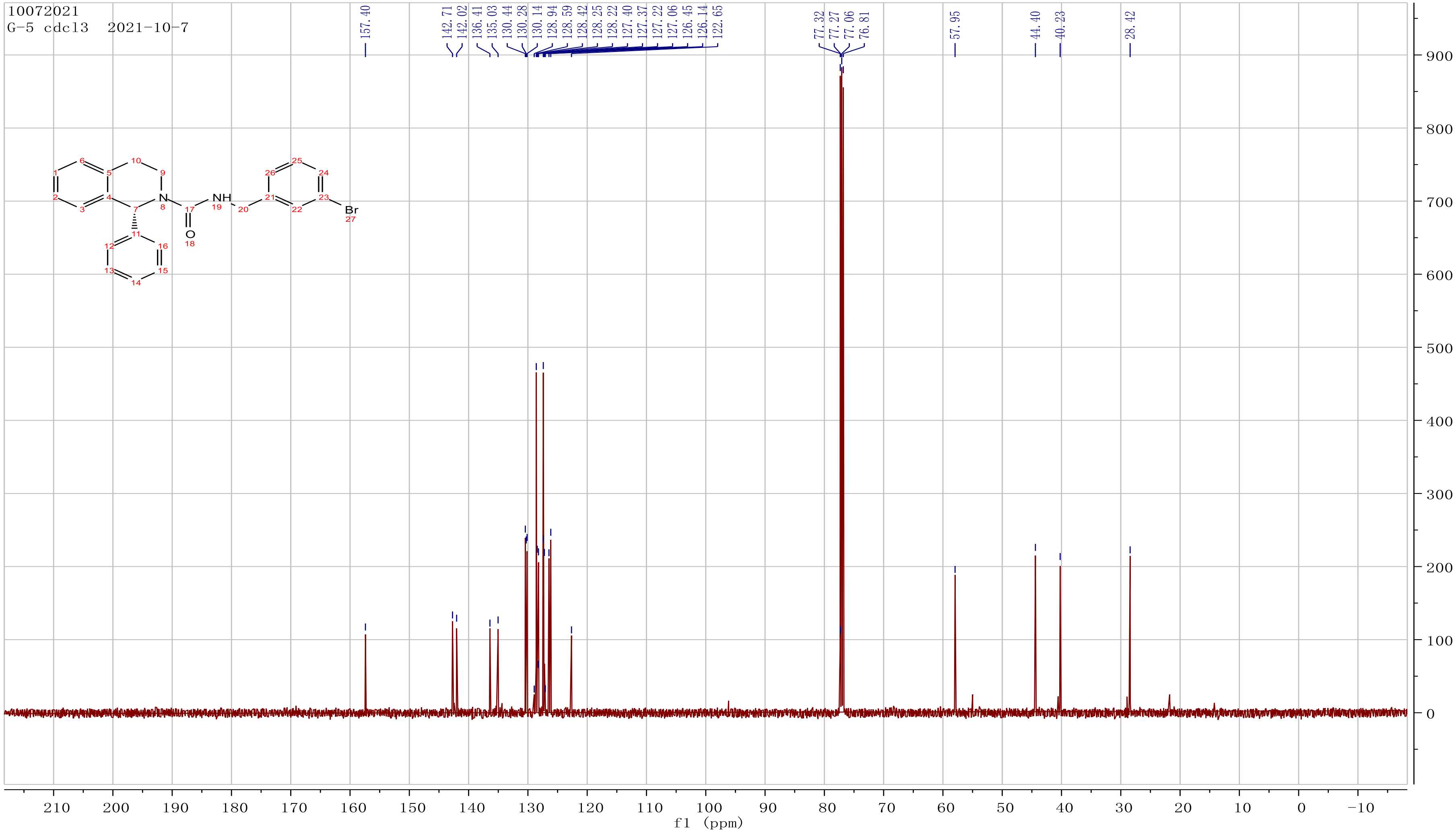

^{13}C-NMR Spectral of **2i**

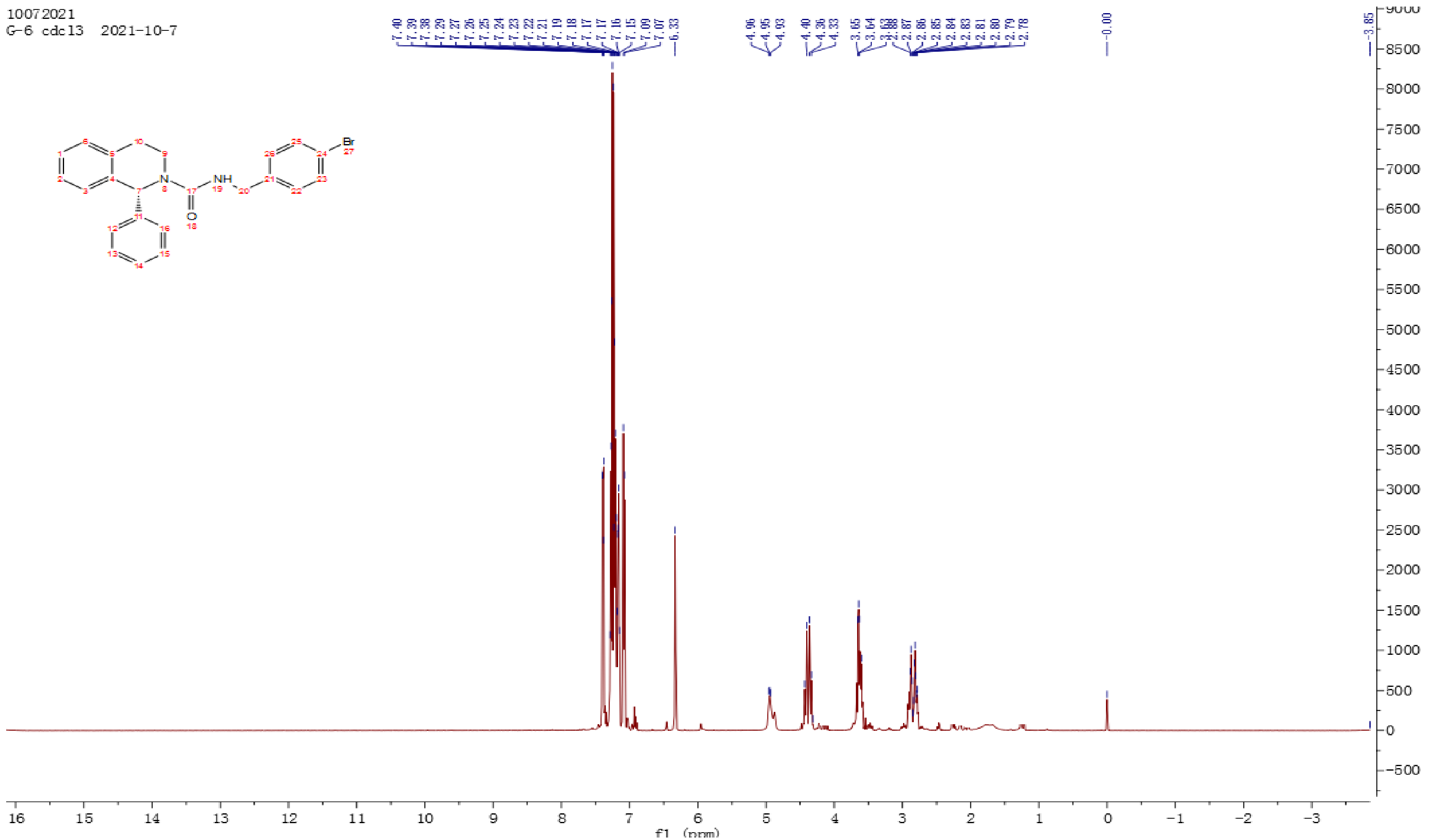

^{1}H-NMR Spectral of **2j**

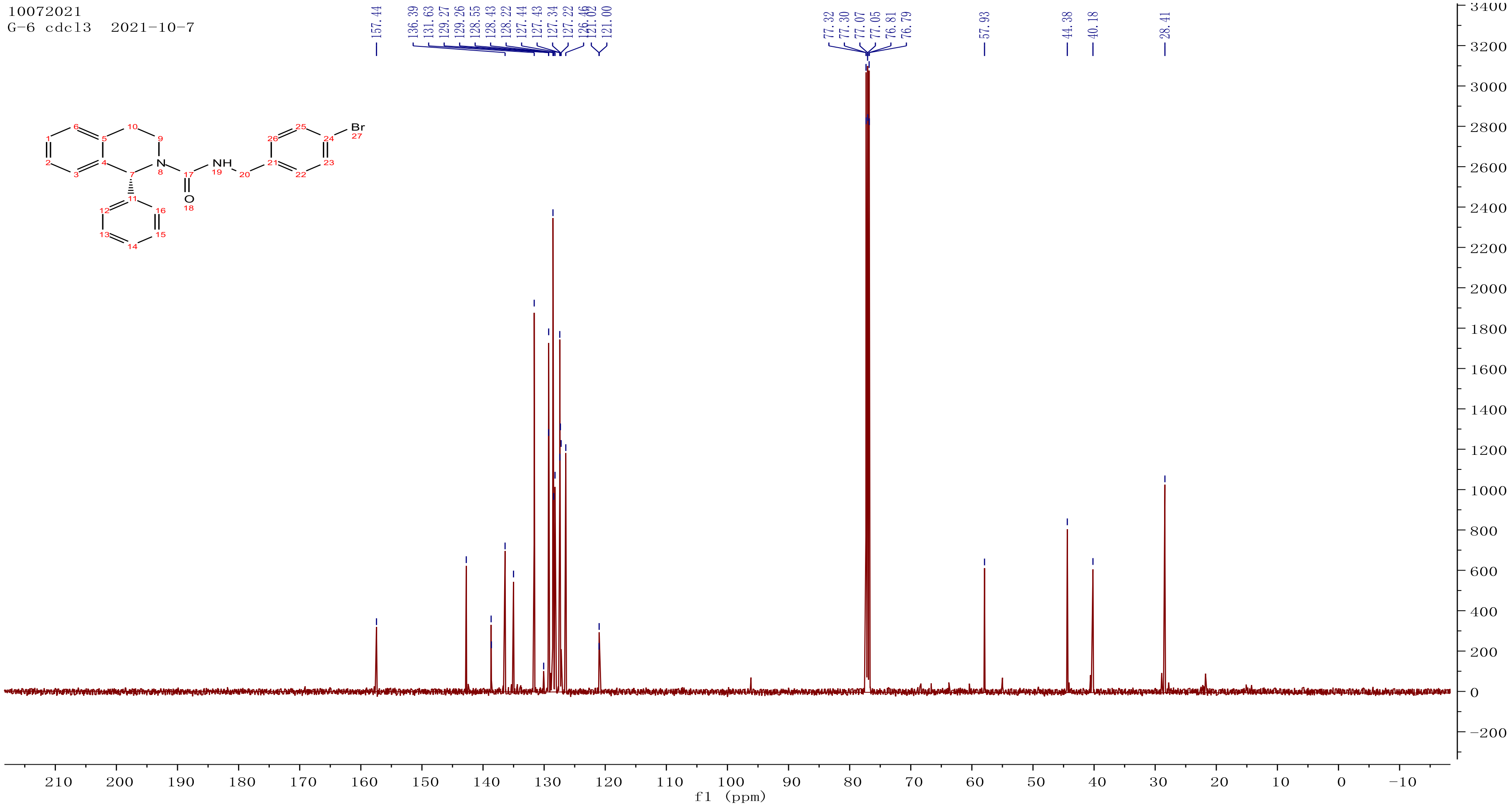

^{13}C-NMR Spectral of **2j**

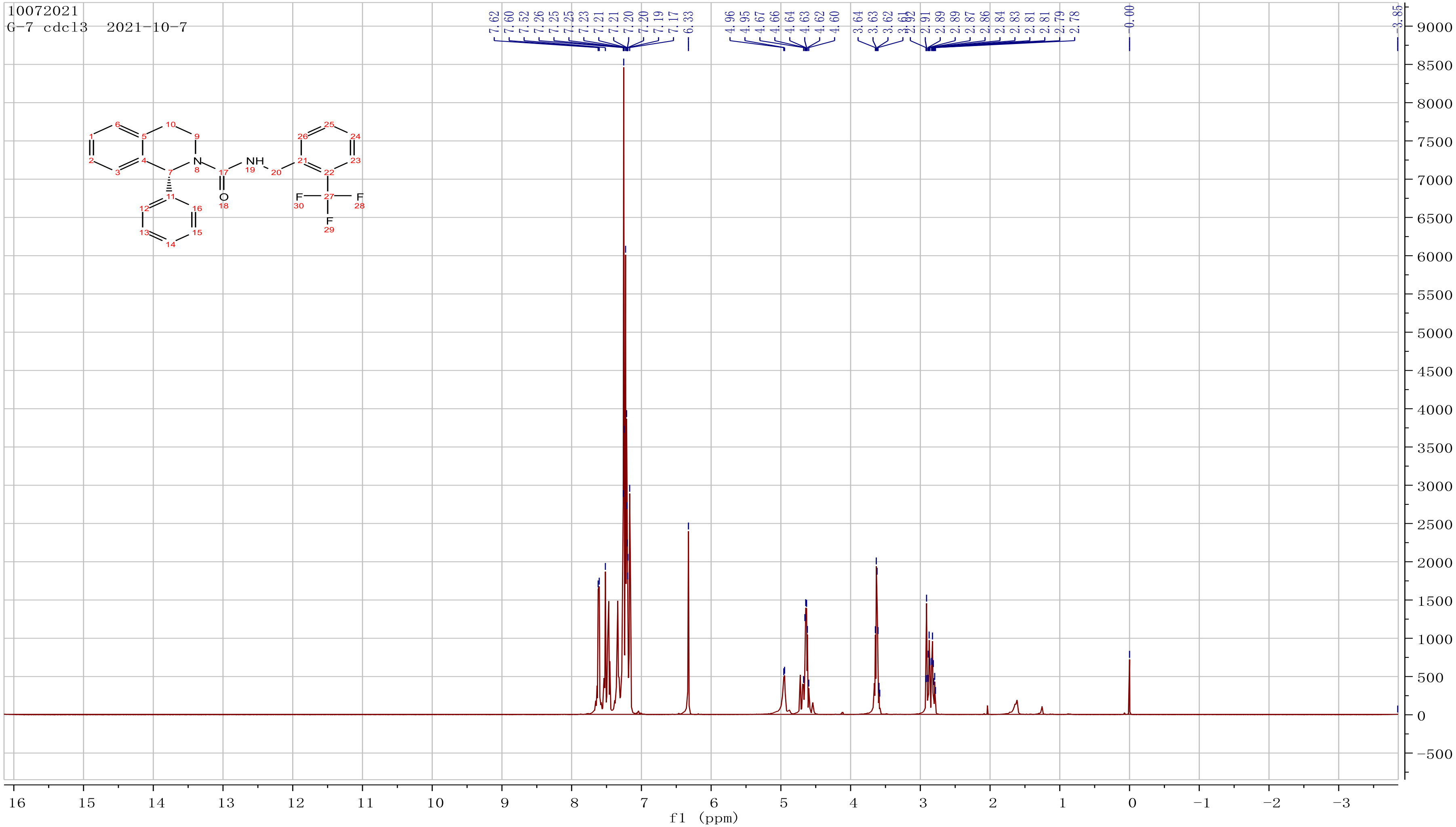

^{1}H-NMR Spectral of **2k**

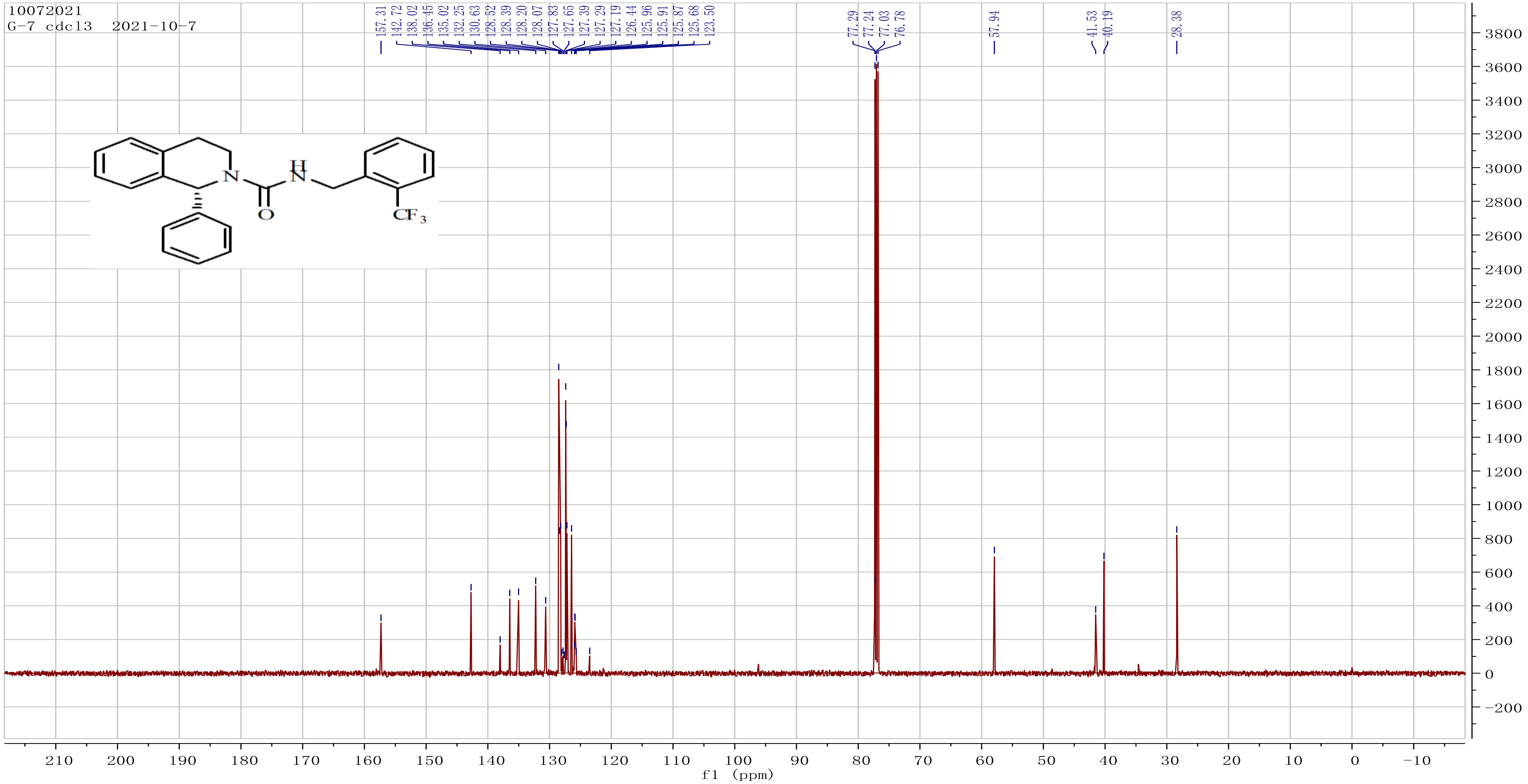

^{13}C-NMR Spectral of **2k**

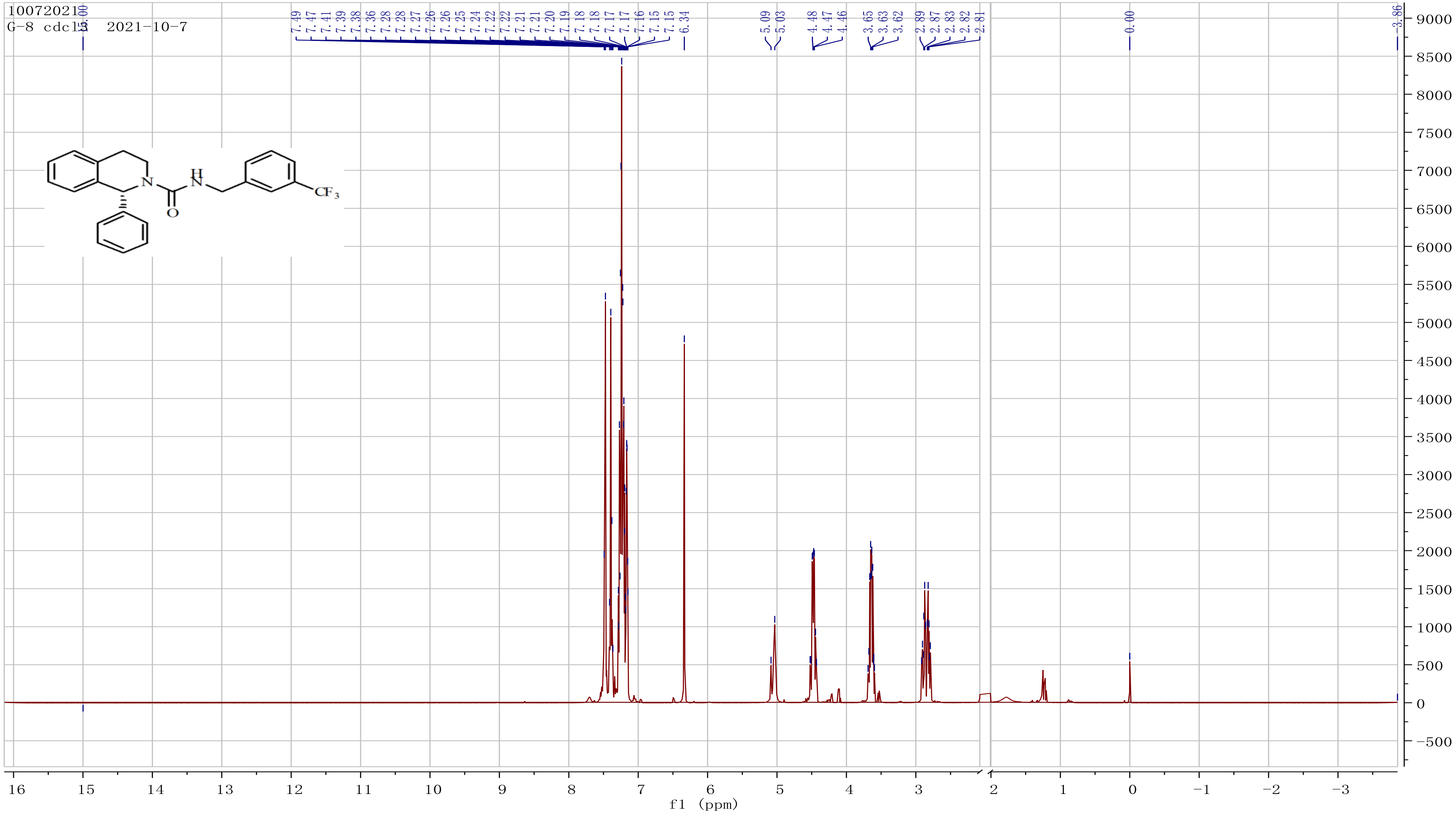

^{1}H-NMR Spectral of **2l**

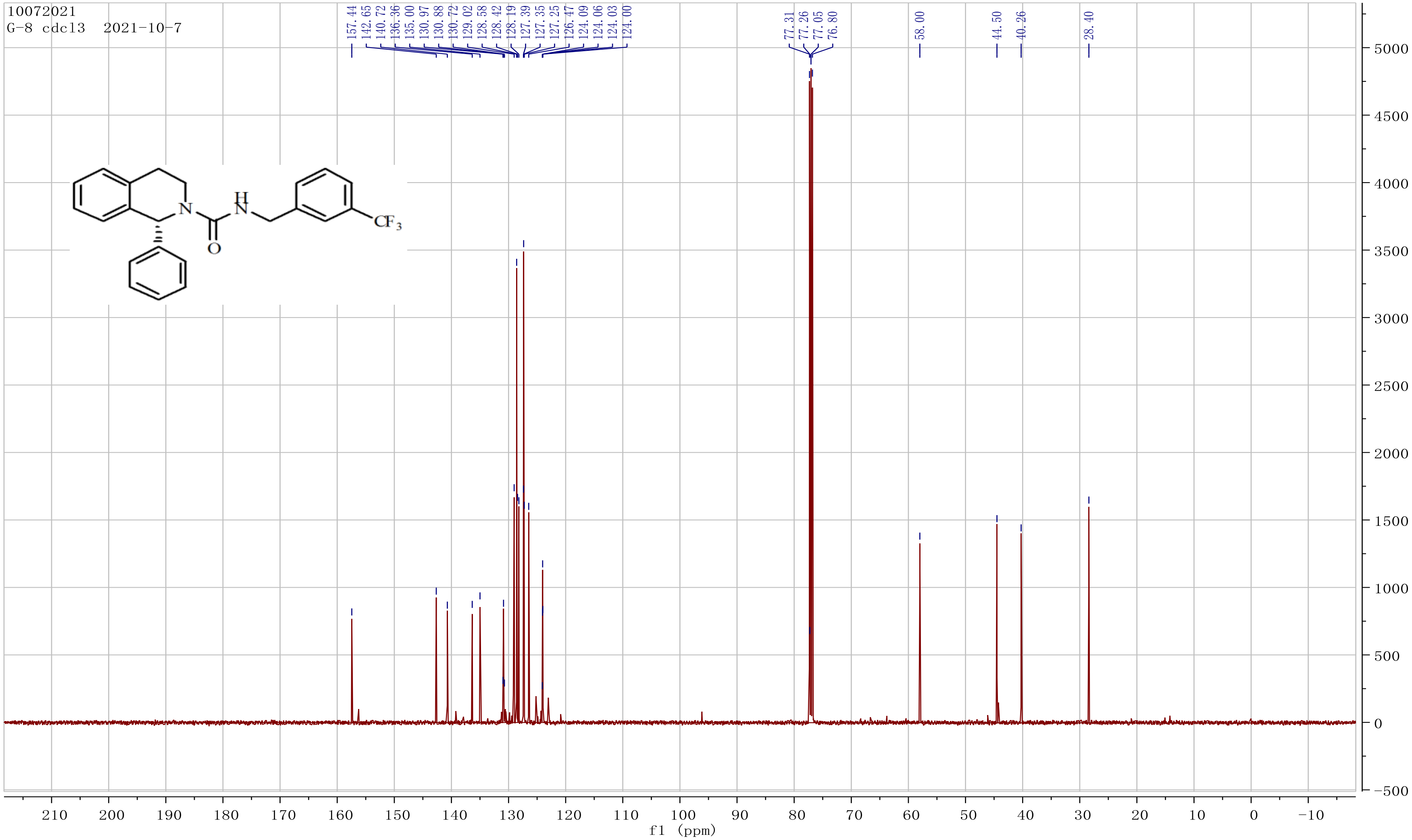

^{13}C-NMR Spectral of **2l**

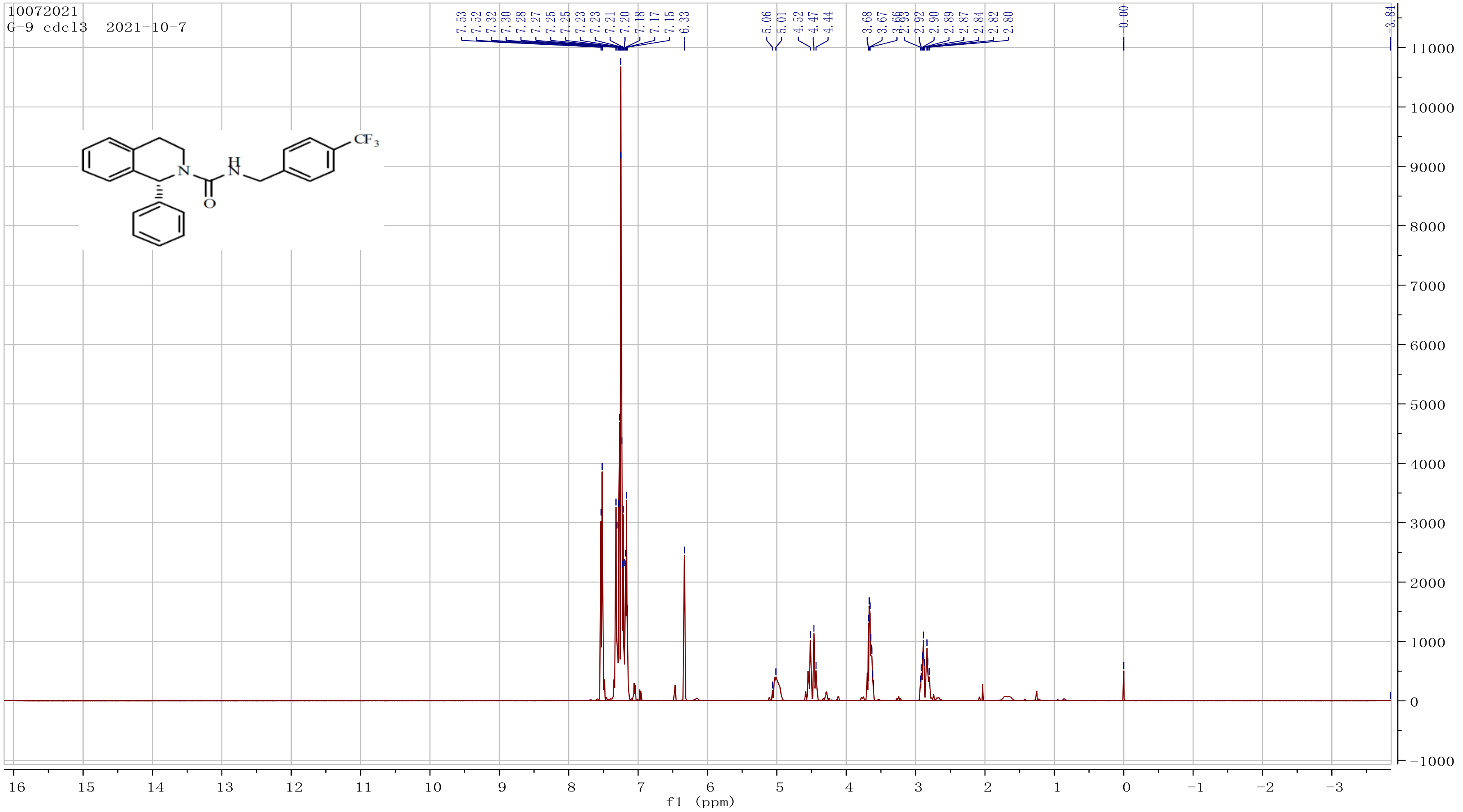

^{1}H-NMR Spectral of **2m**

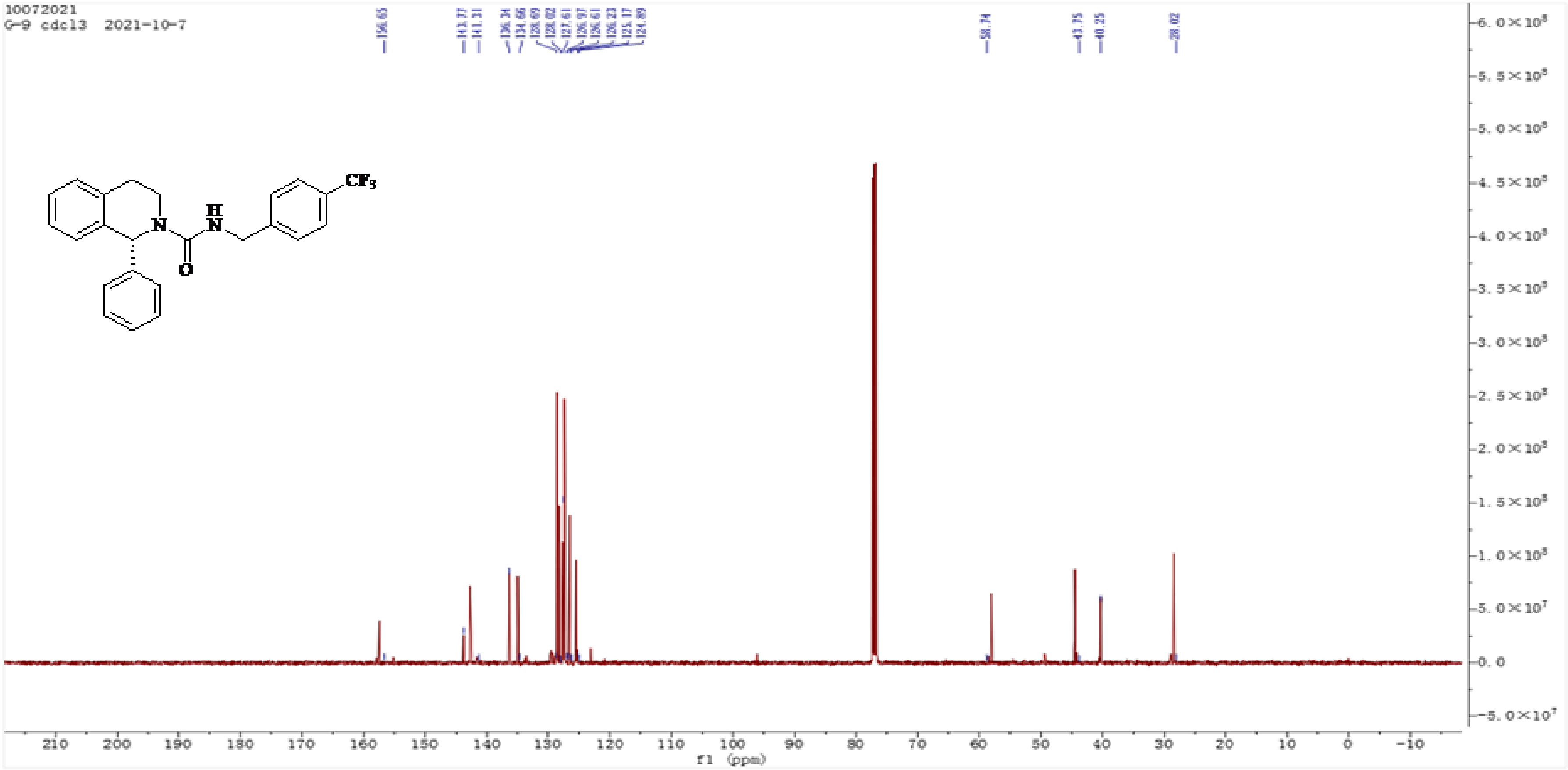

^{13}C-NMR Spectral of **2m**

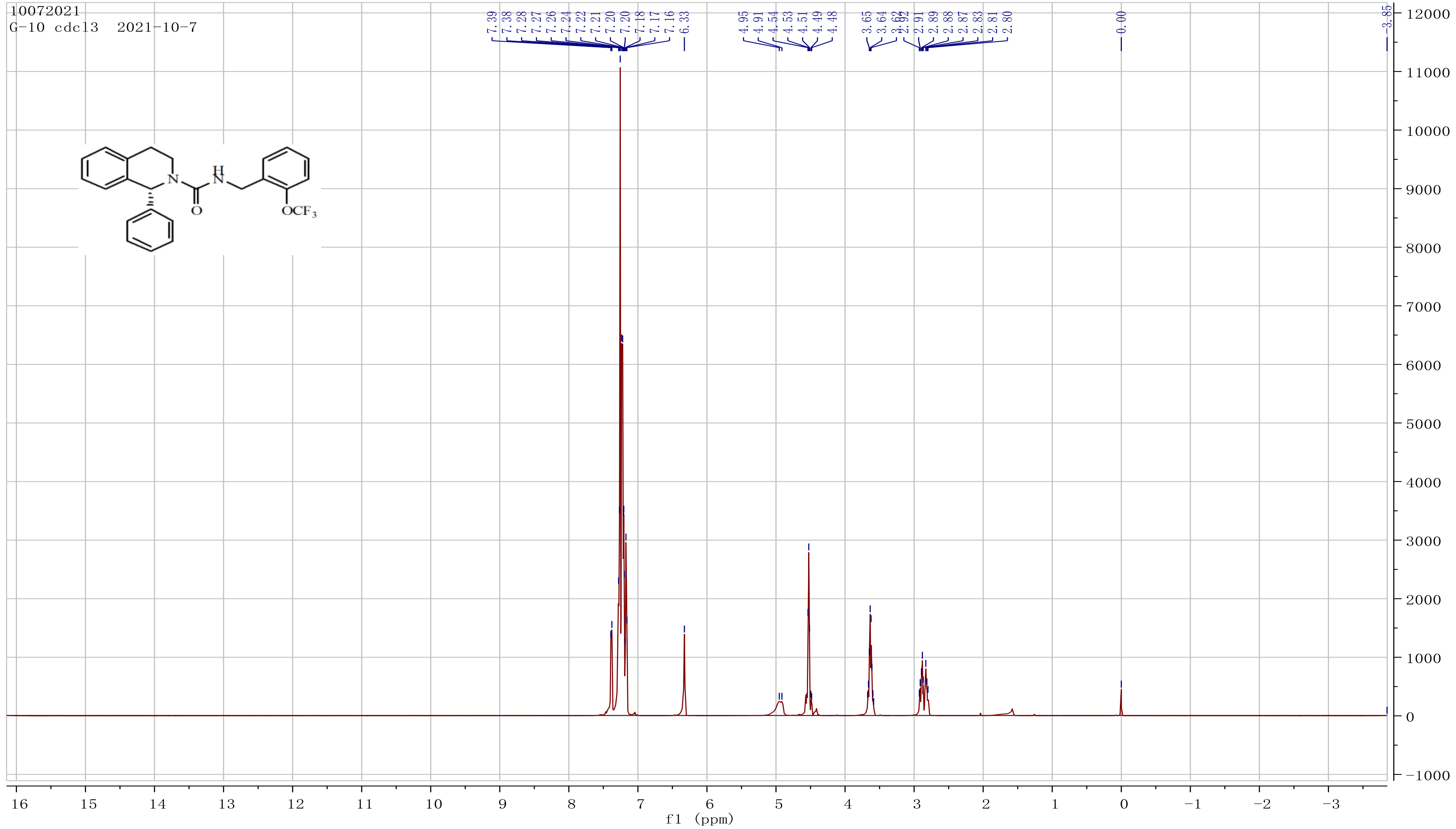

^{1}H-NMR Spectral of **2n**

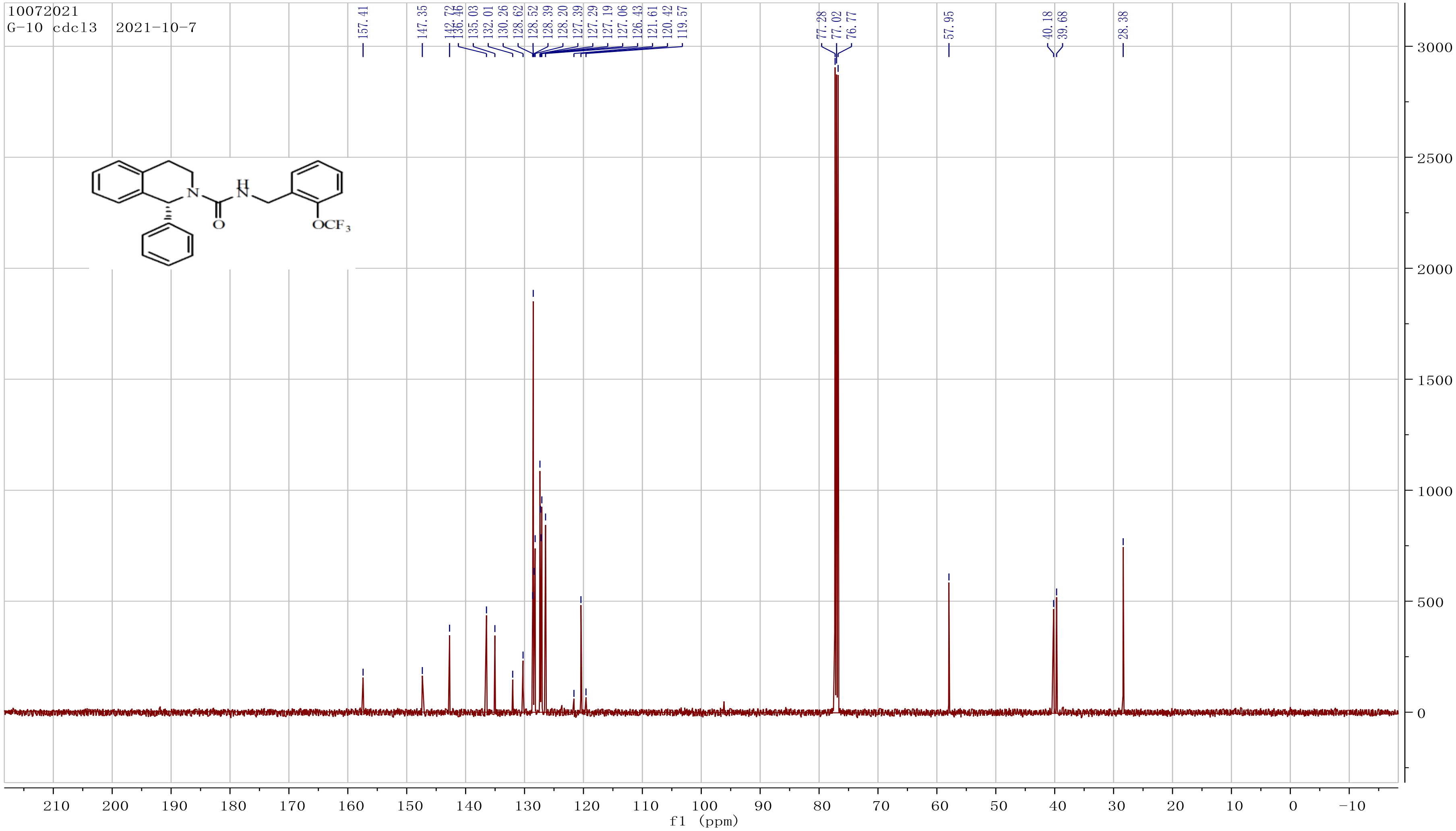

^{13}C-NMR Spectral of **2n**

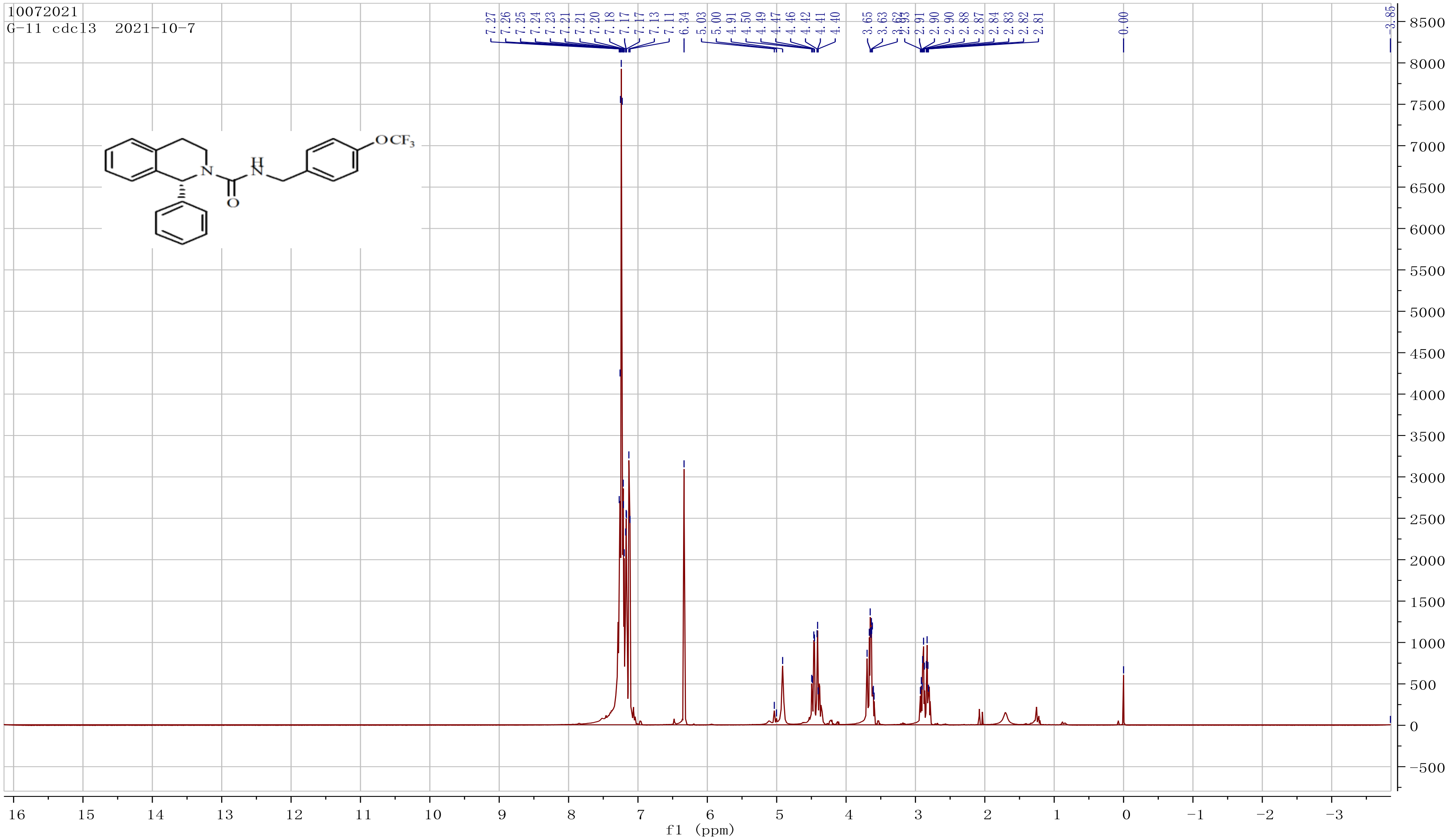

^{1}H-NMR Spectral of **2o**

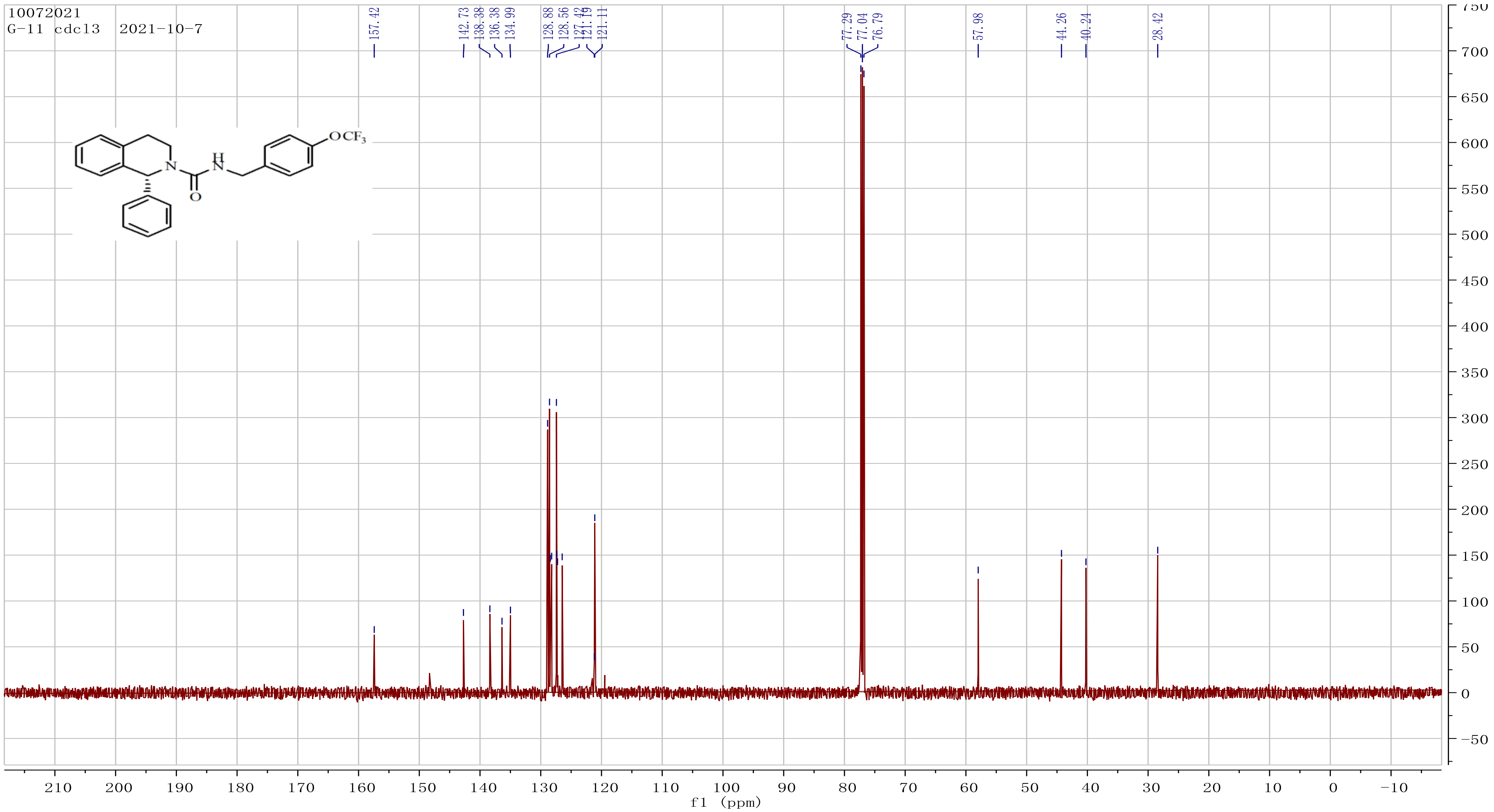

^{13}C-NMR Spectral of **2o**

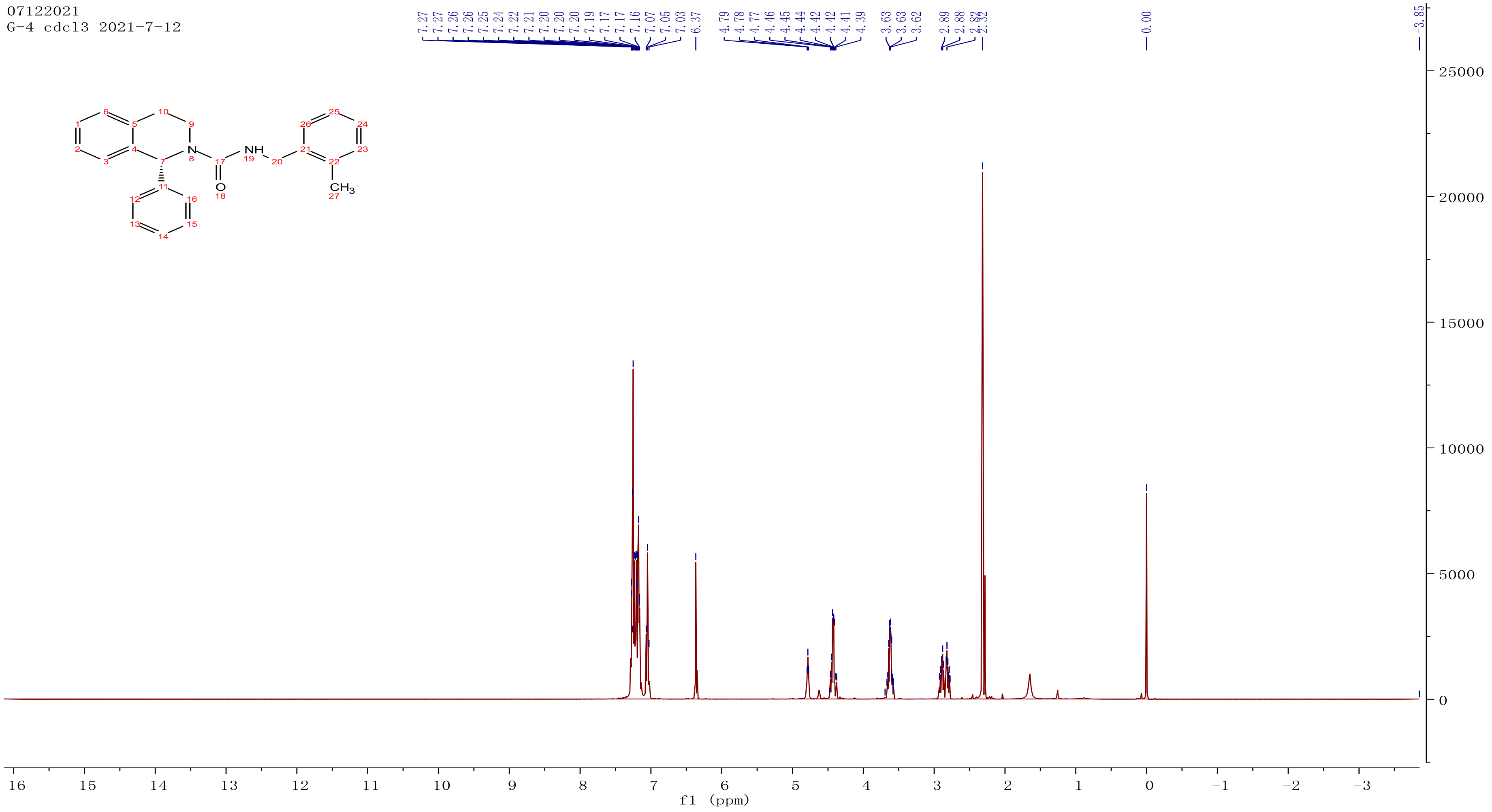

^{1}H-NMR Spectral of **2p**

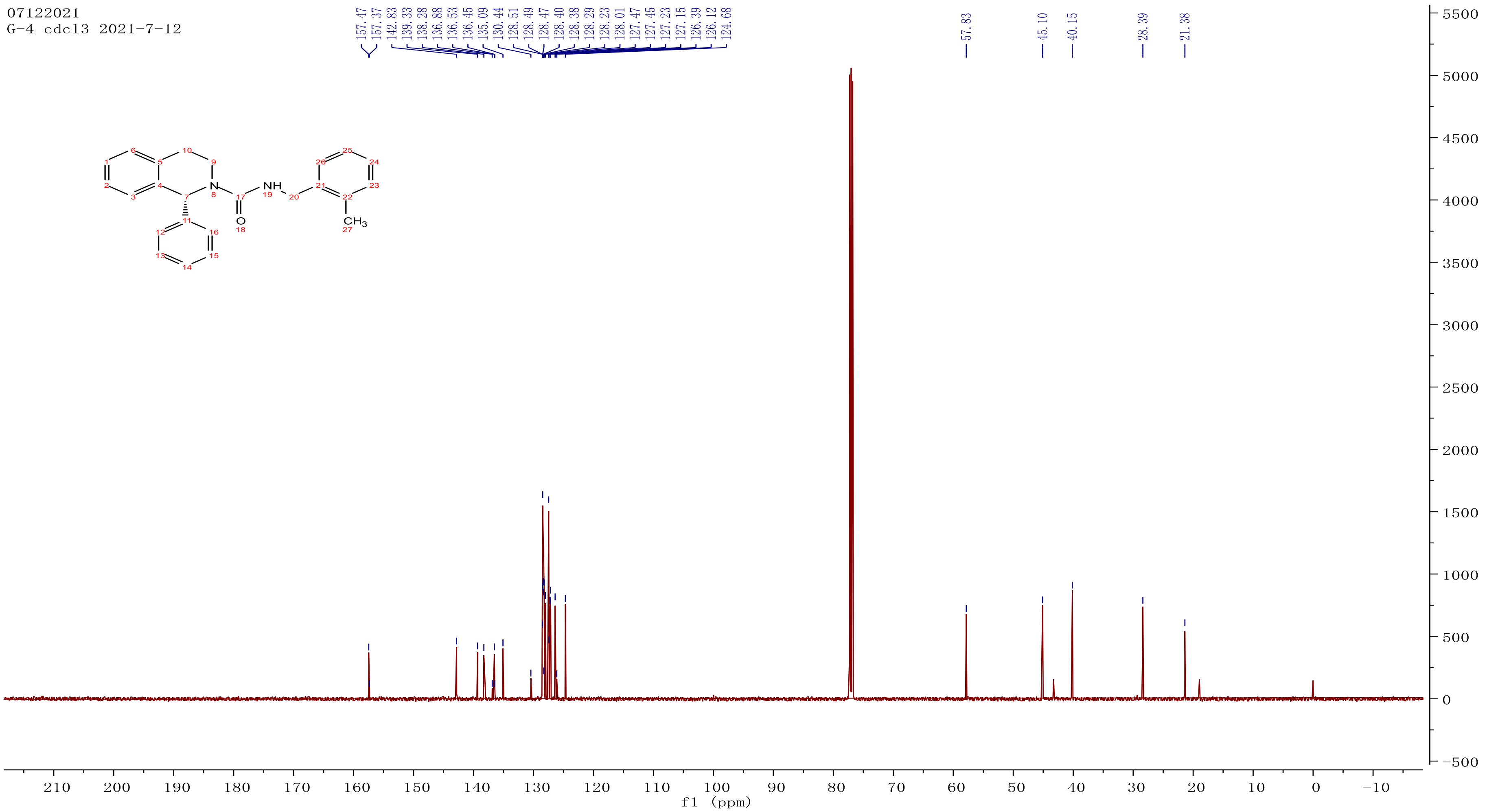

^{13}C-NMR Spectral of **2p**

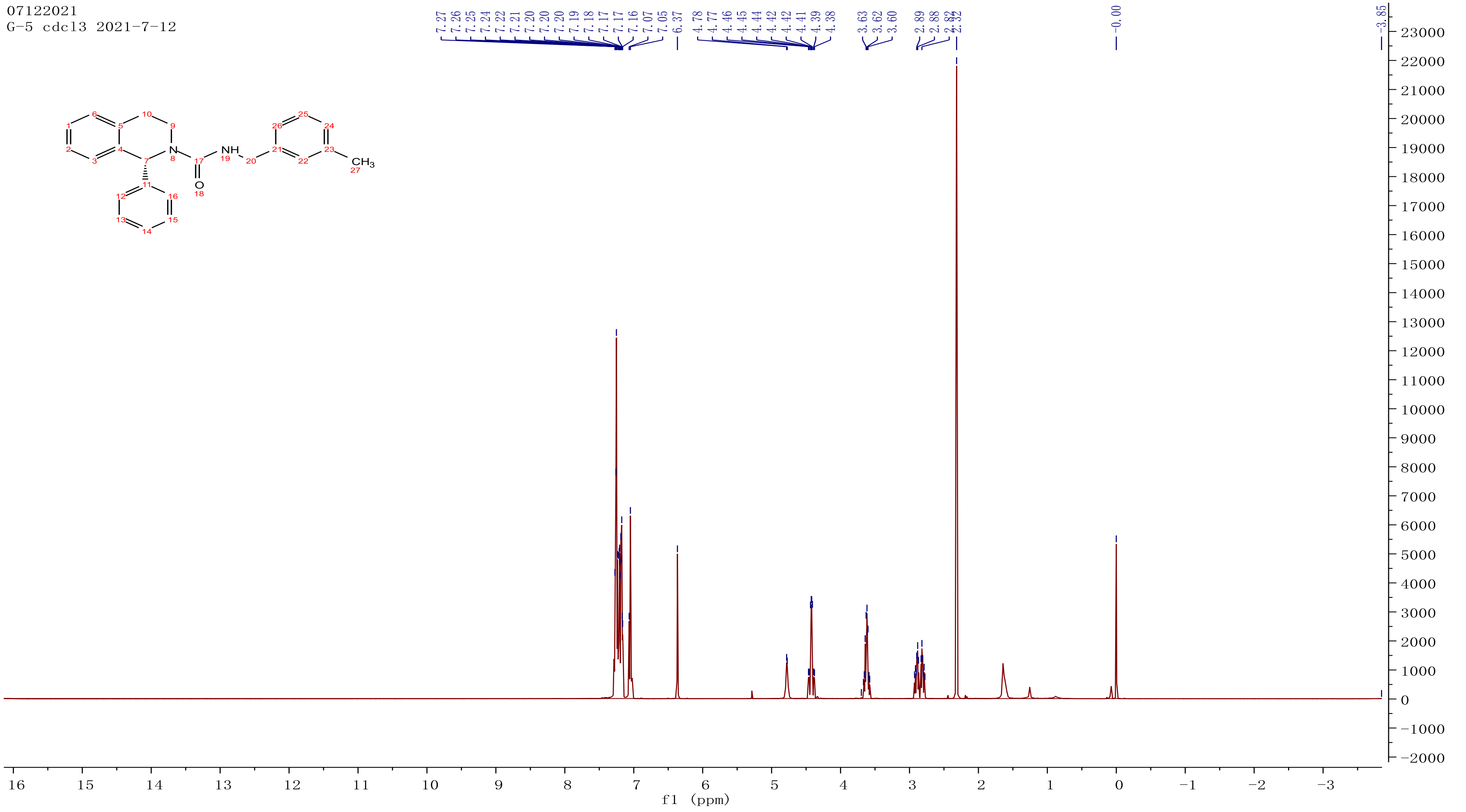

^{1}H-NMR Spectral of **2q**

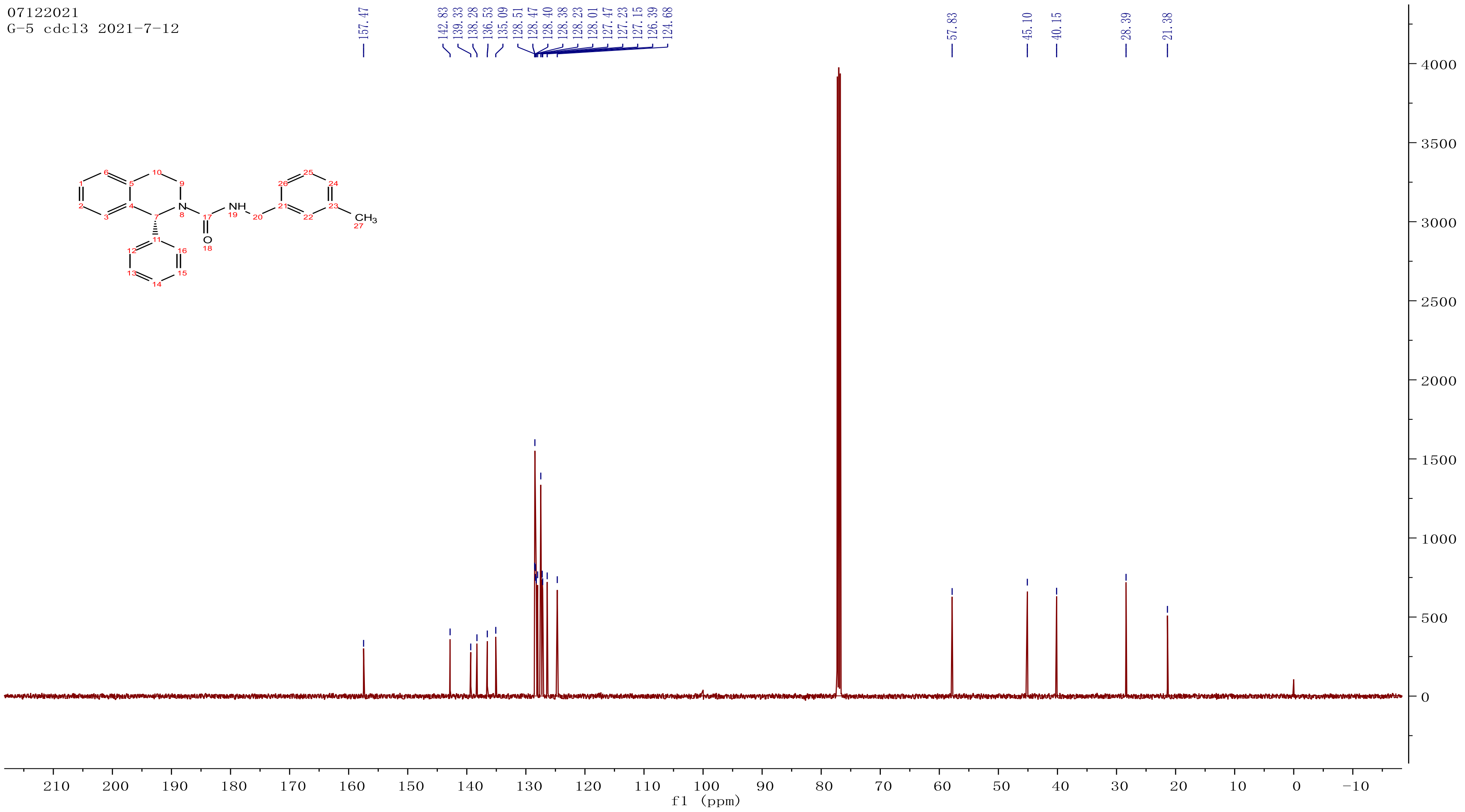

^{13}C-NMR Spectral of **2q**

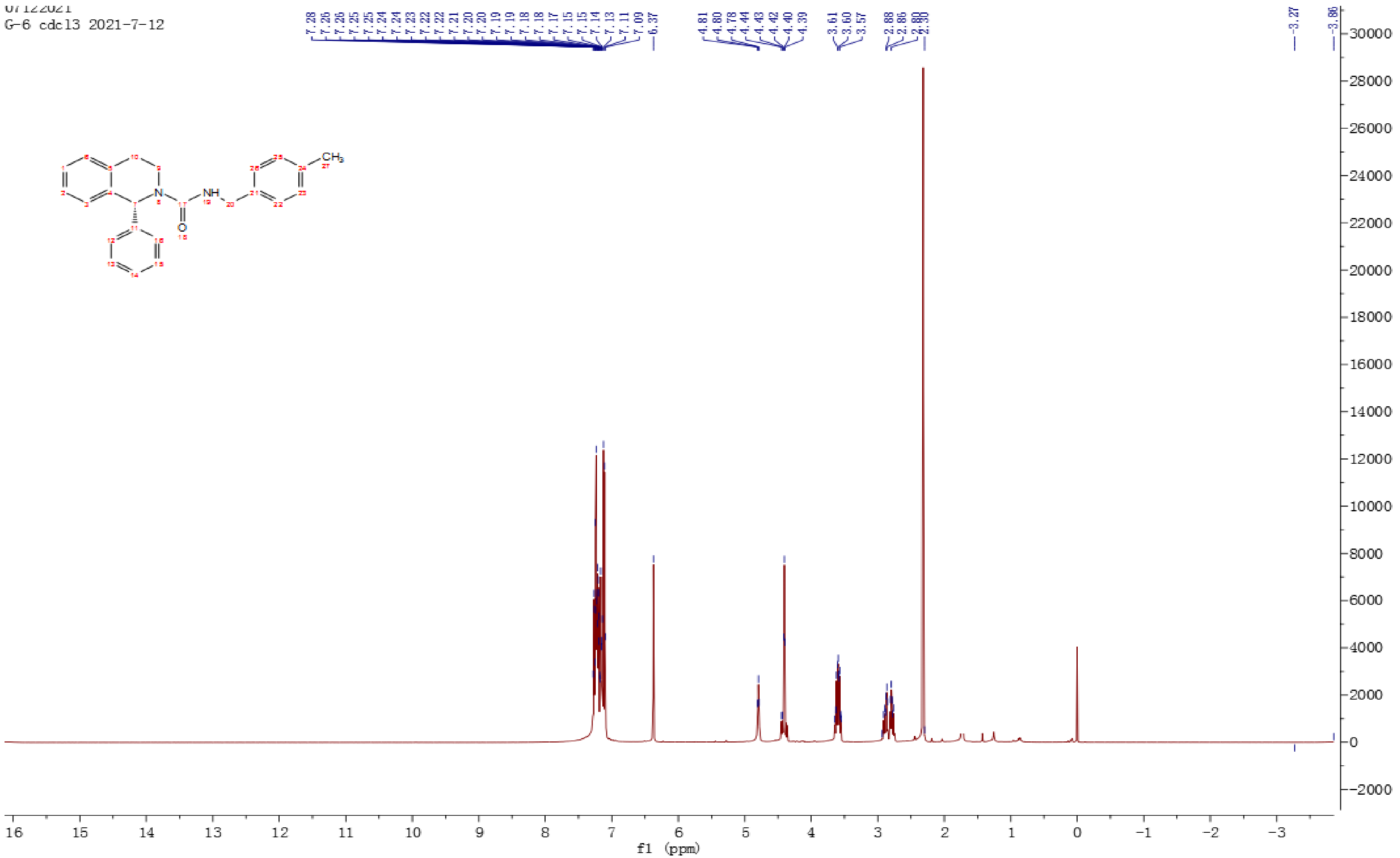

^{1}H-NMR Spectral of **2r**

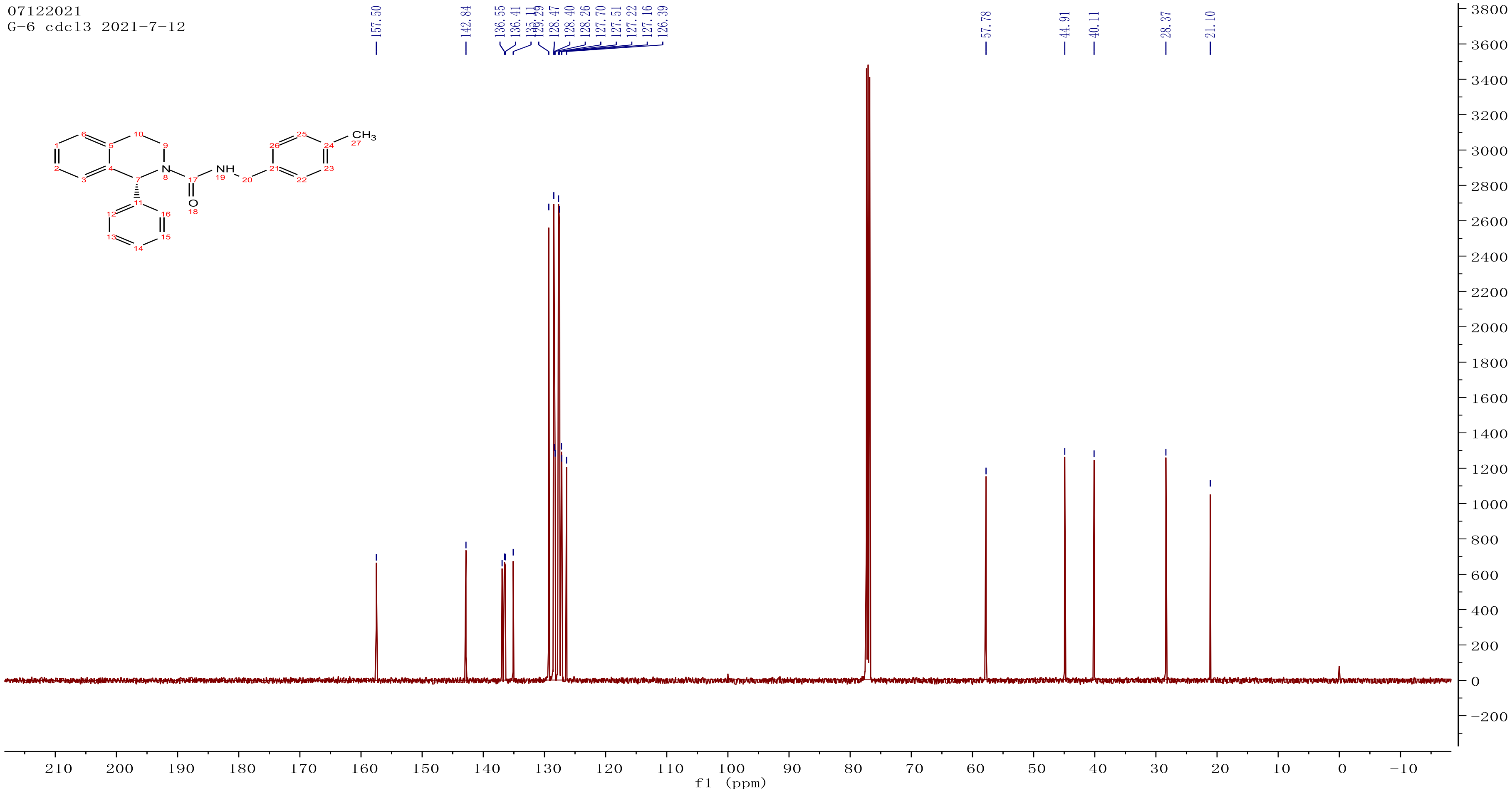

^{13}C-NMR Spectral of **2r**

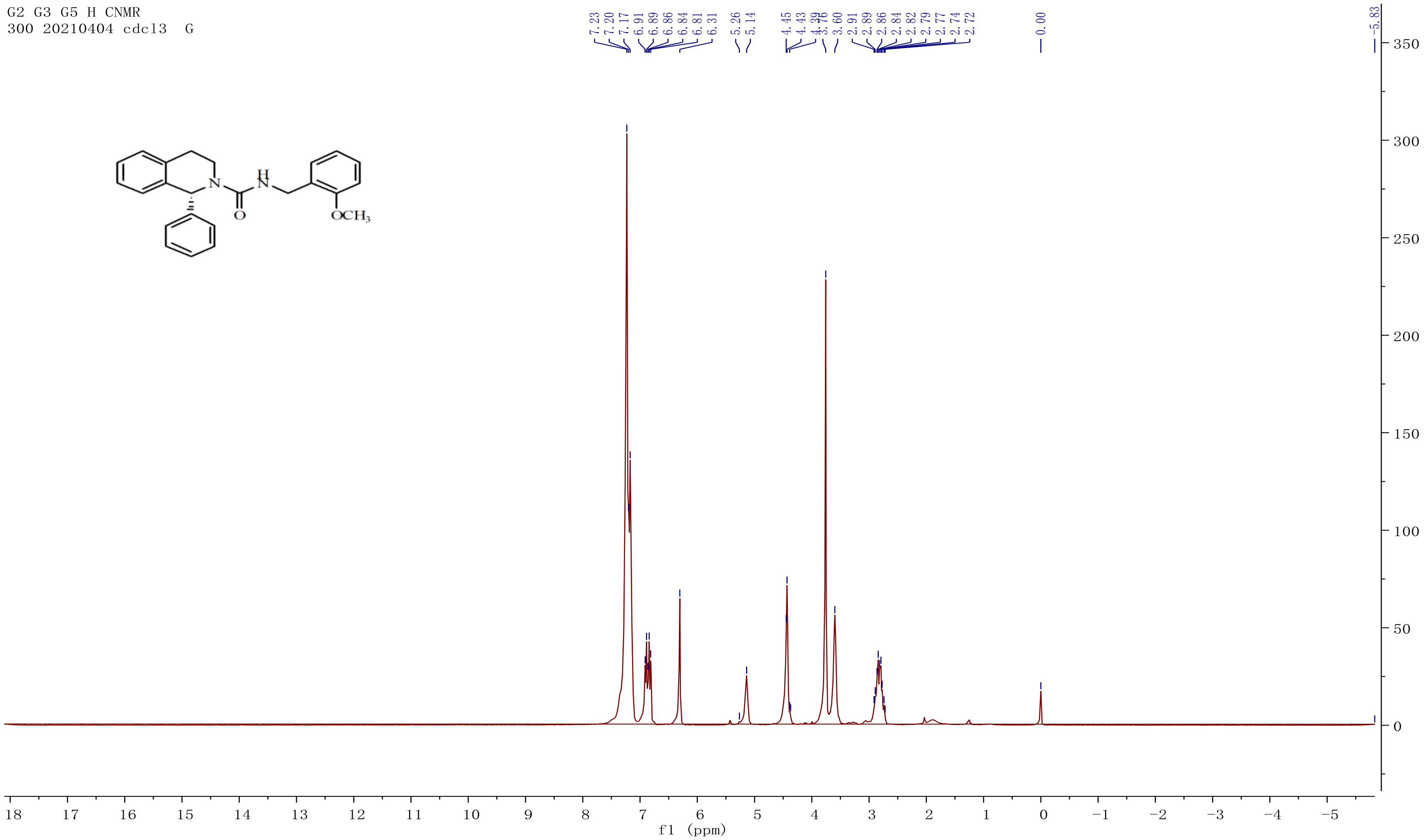

^{1}H-NMR Spectral of **2s**

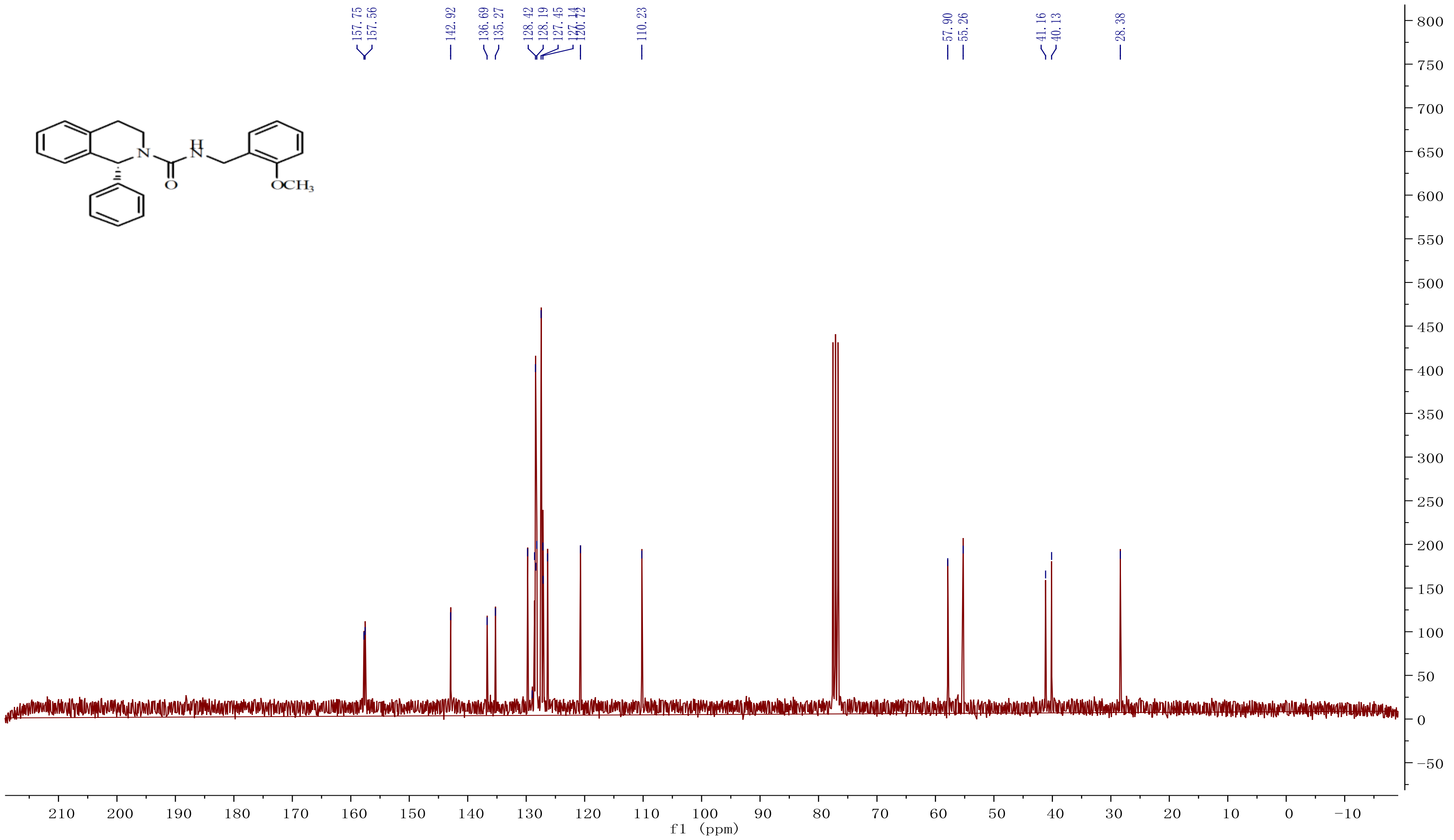

^{13}C-NMR Spectral of **2s**

07122021
G-7 cdcl3 2021-7-12

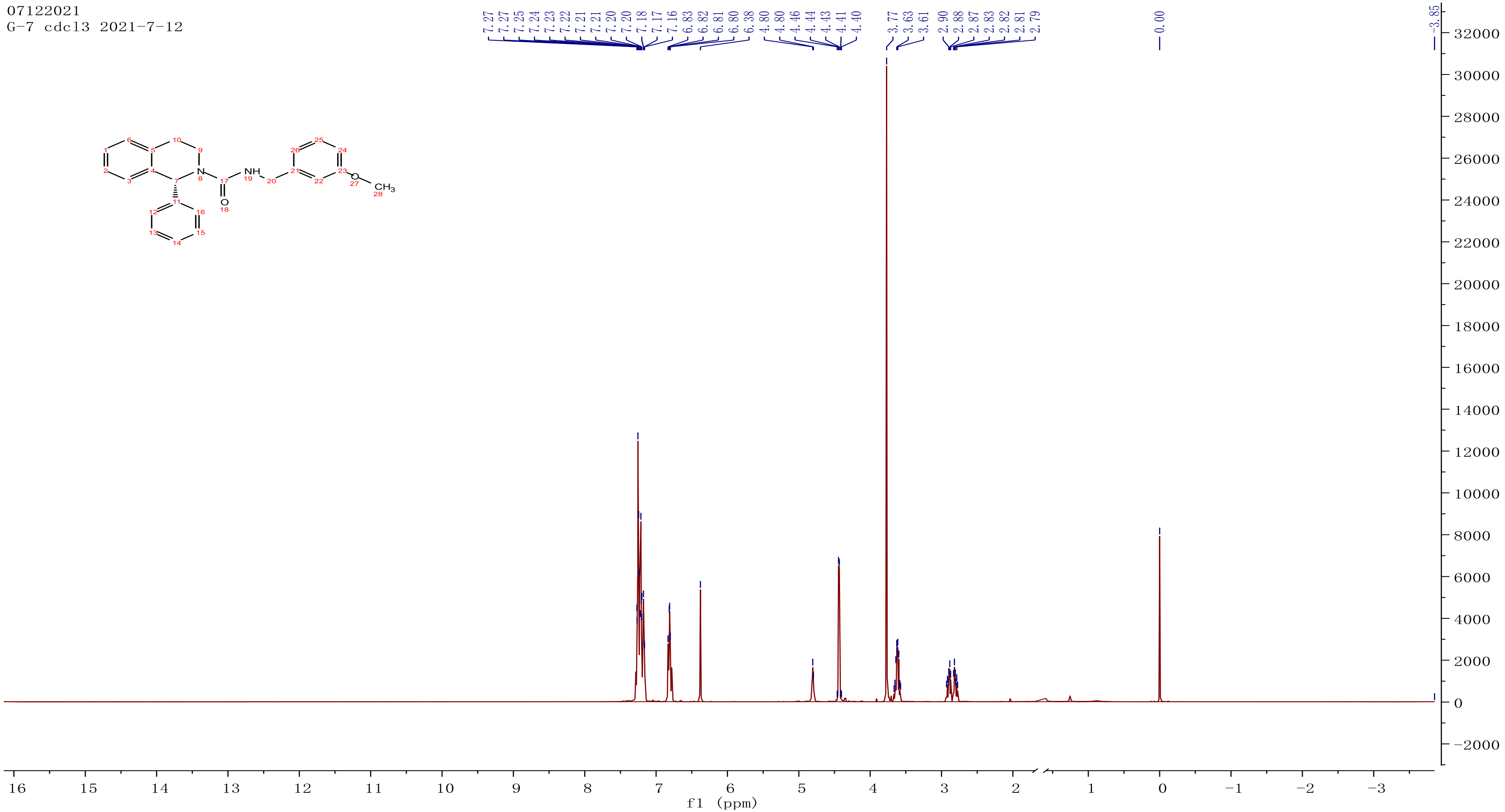

^{1}H-NMR Spectral of **2t**

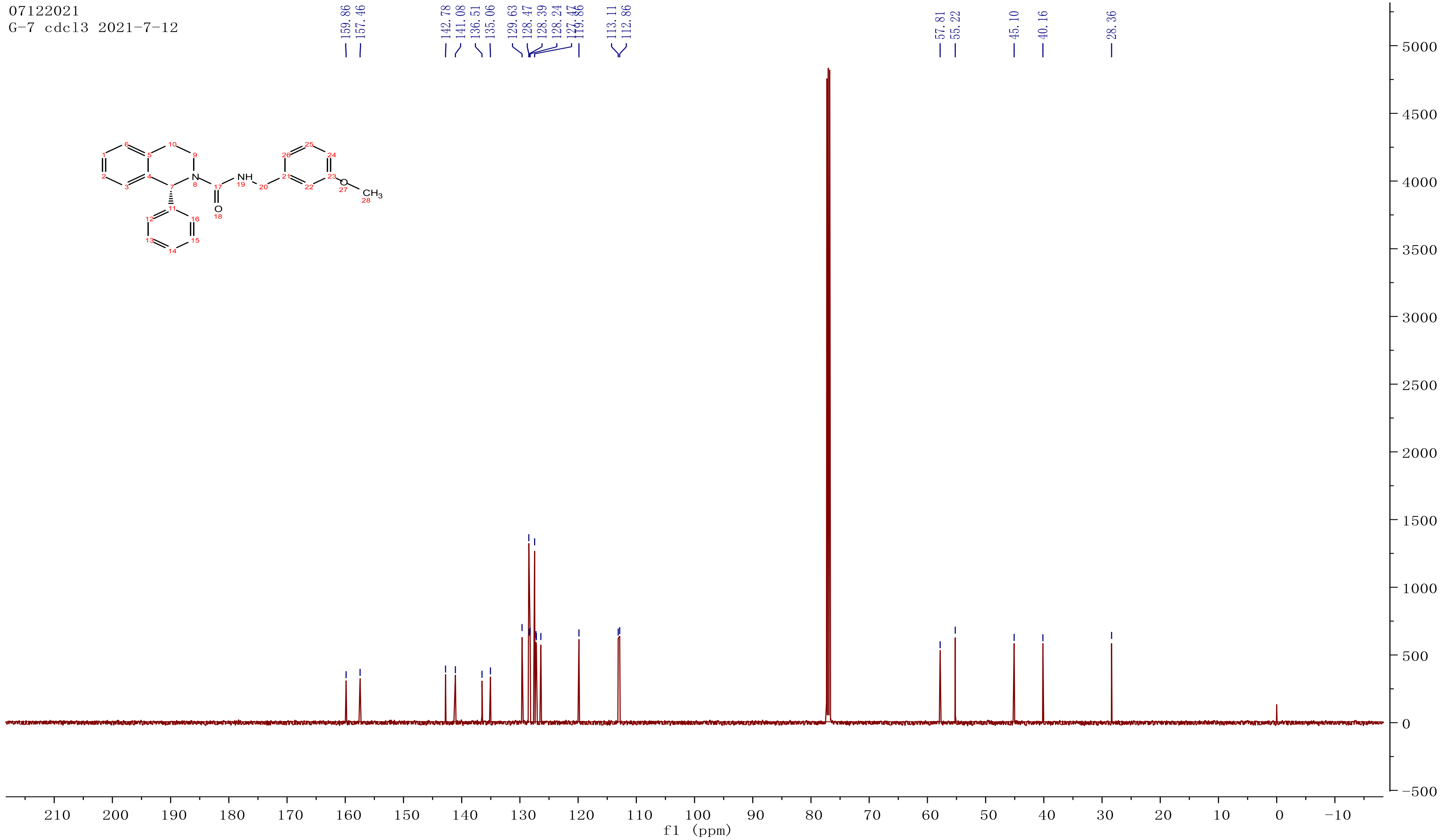

^{13}C-NMR Spectral of **2t**

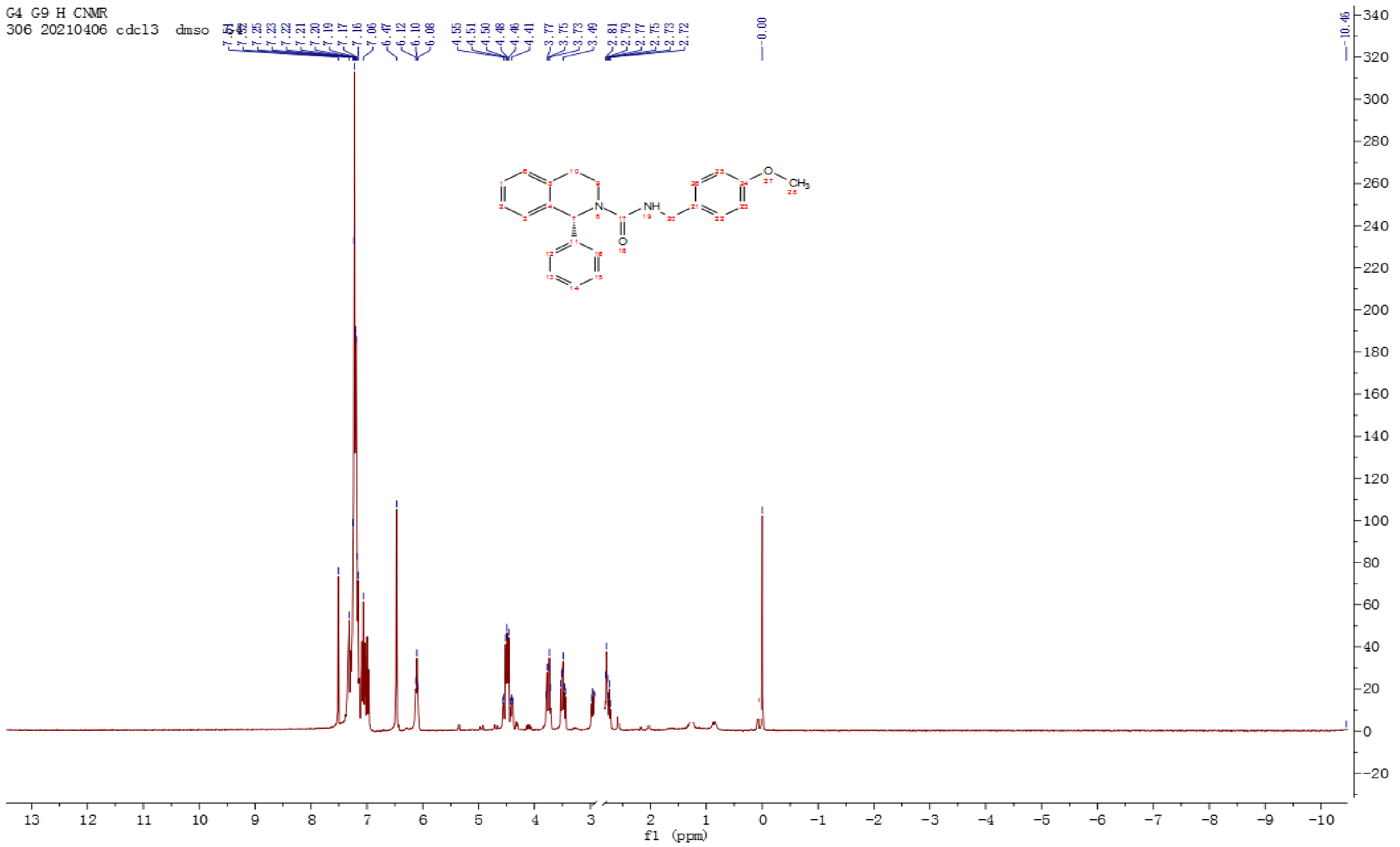

^{1}H-NMR Spectral of **2u**

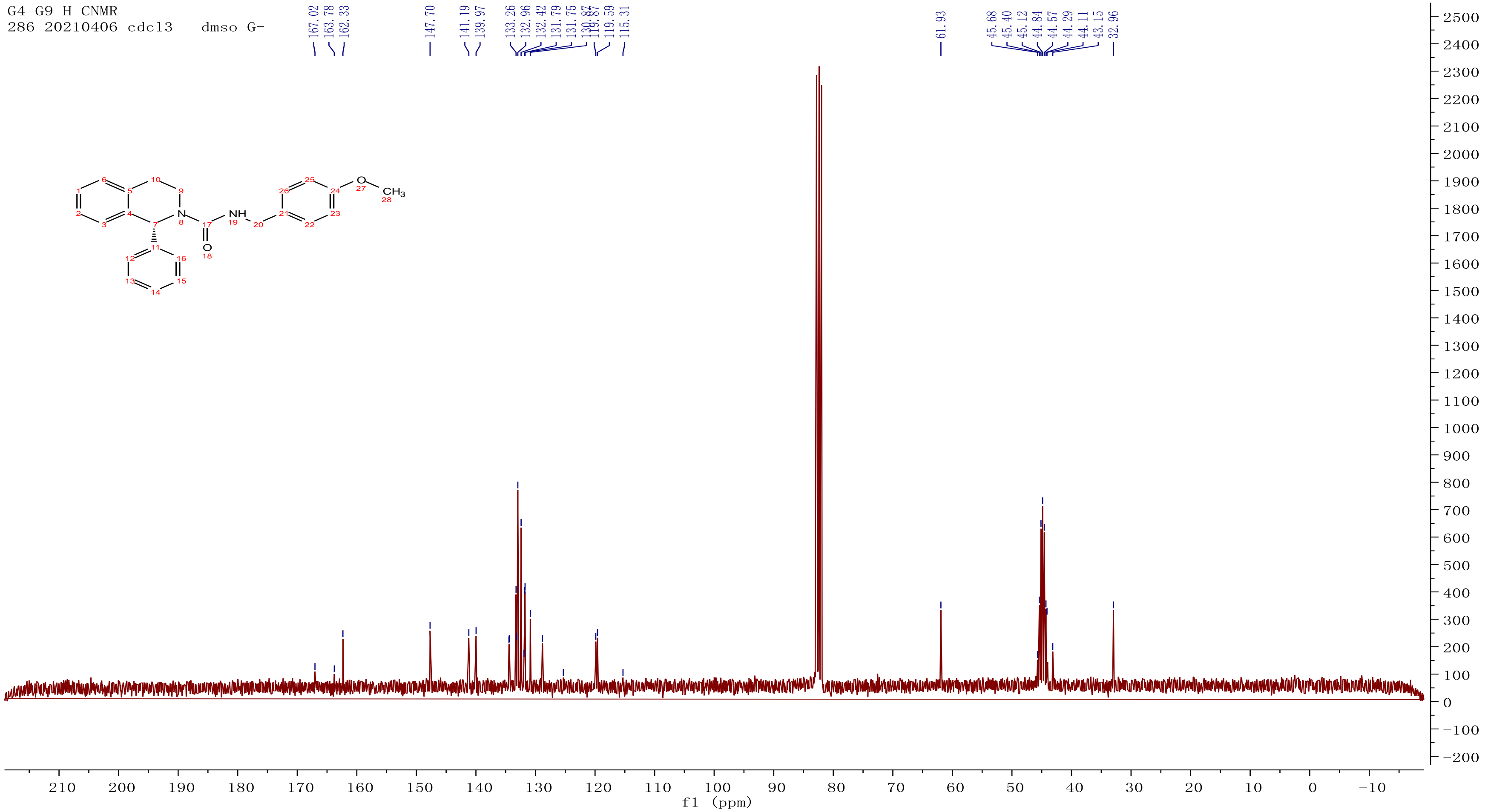

^{13}C-NMR Spectral of **2u**

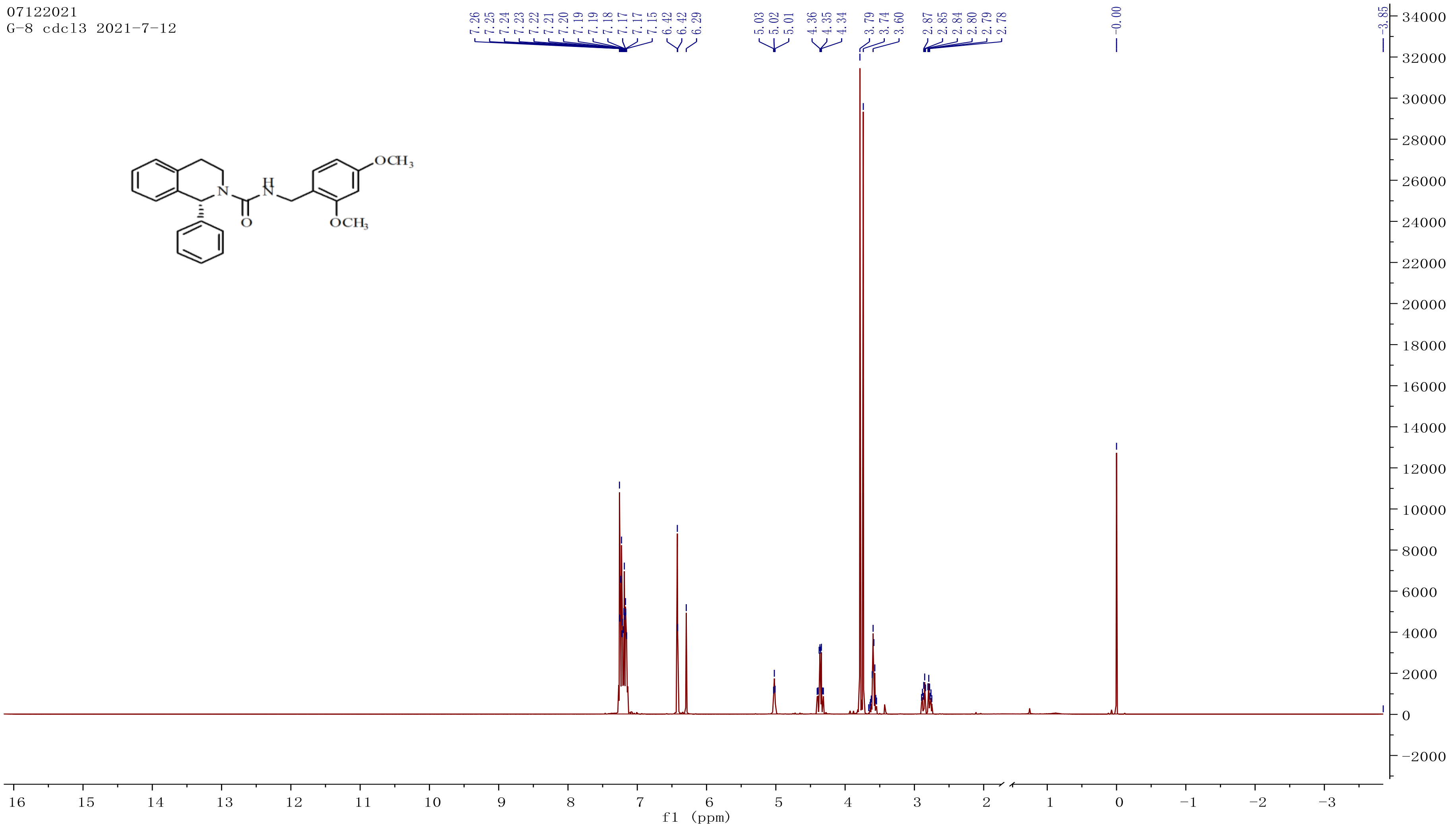

^{1}H-NMR Spectral of **2v**

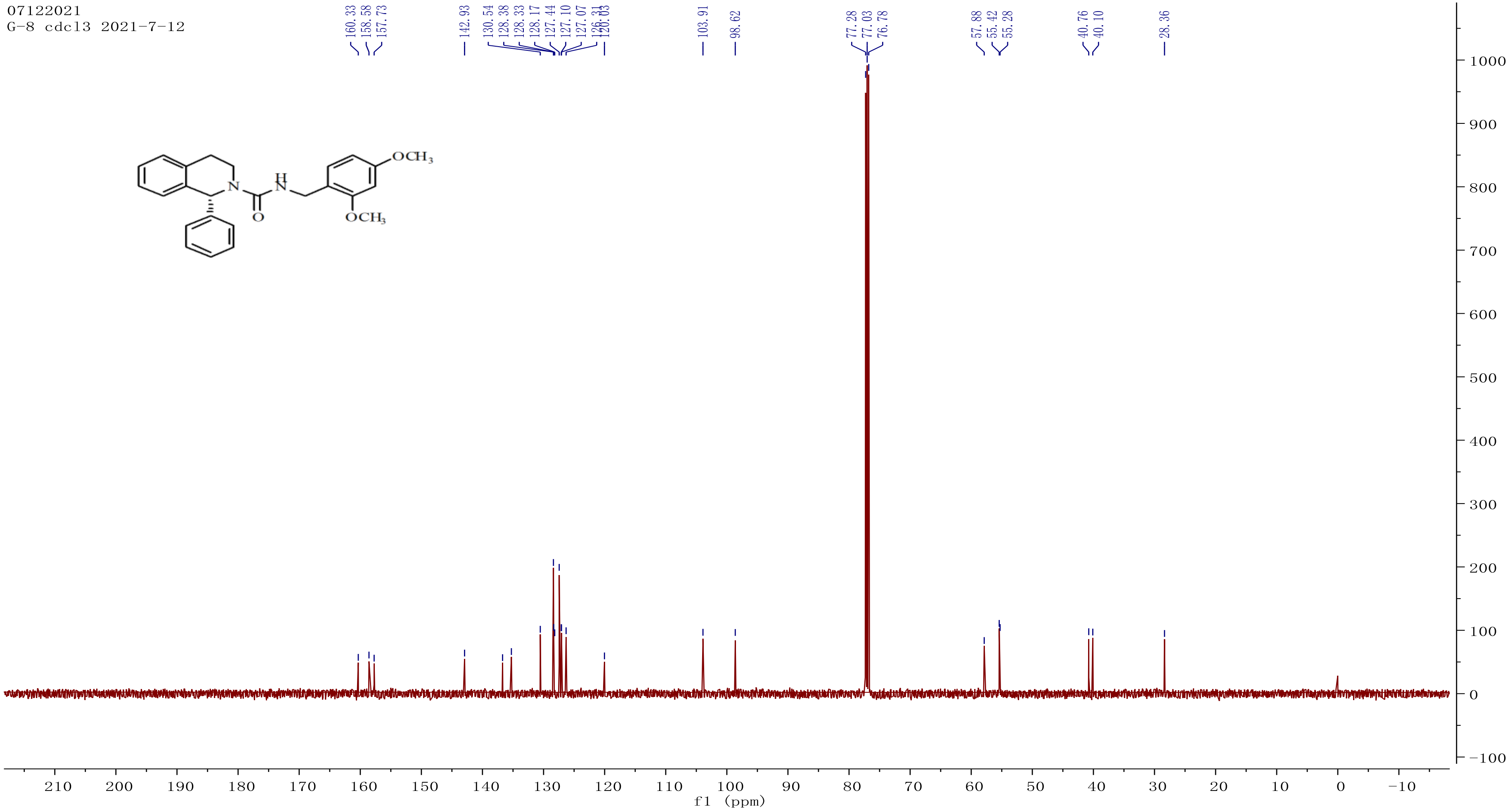

^{13}C-NMR Spectral of **2v**

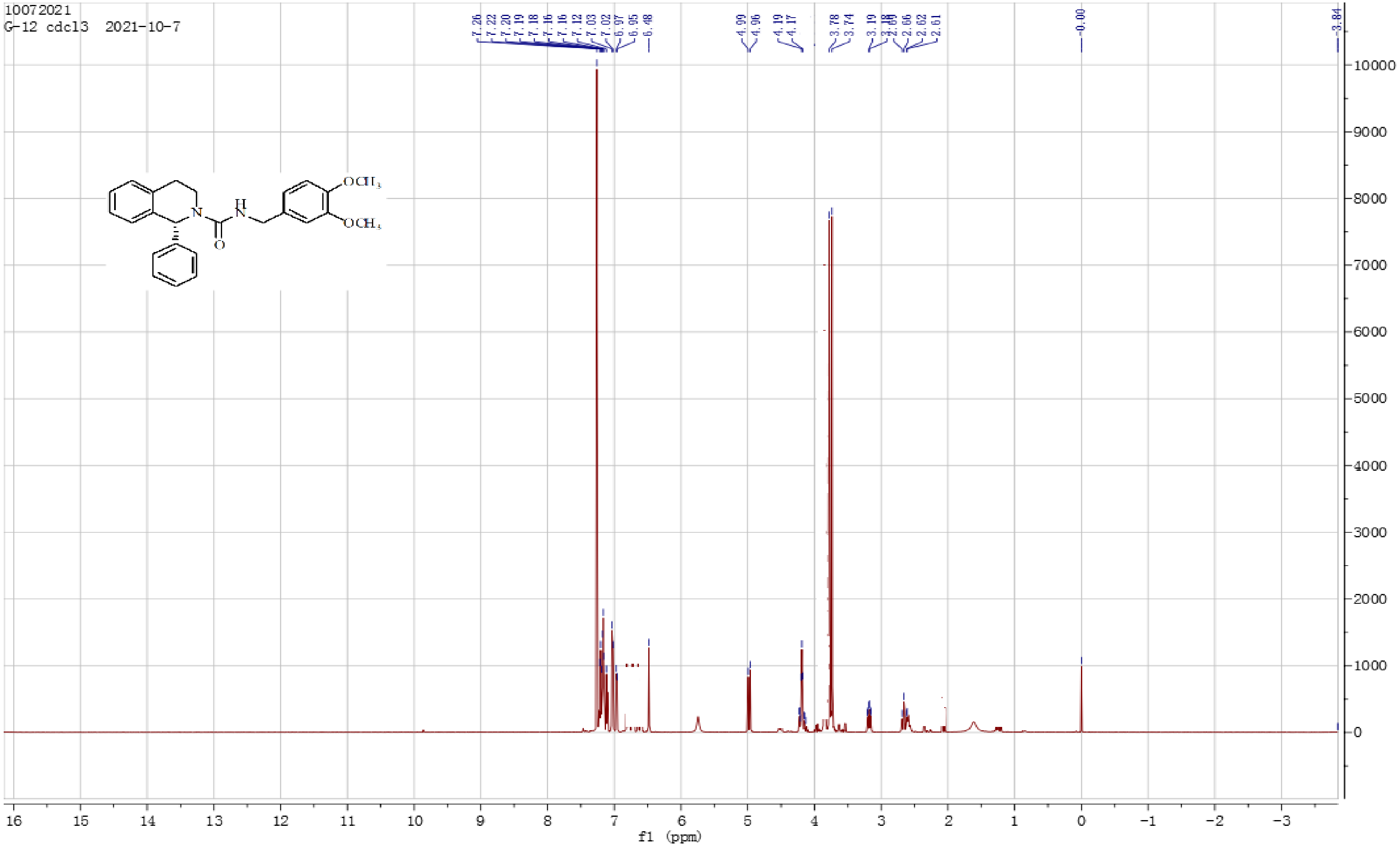

^{1}H-NMR Spectral of **2w**

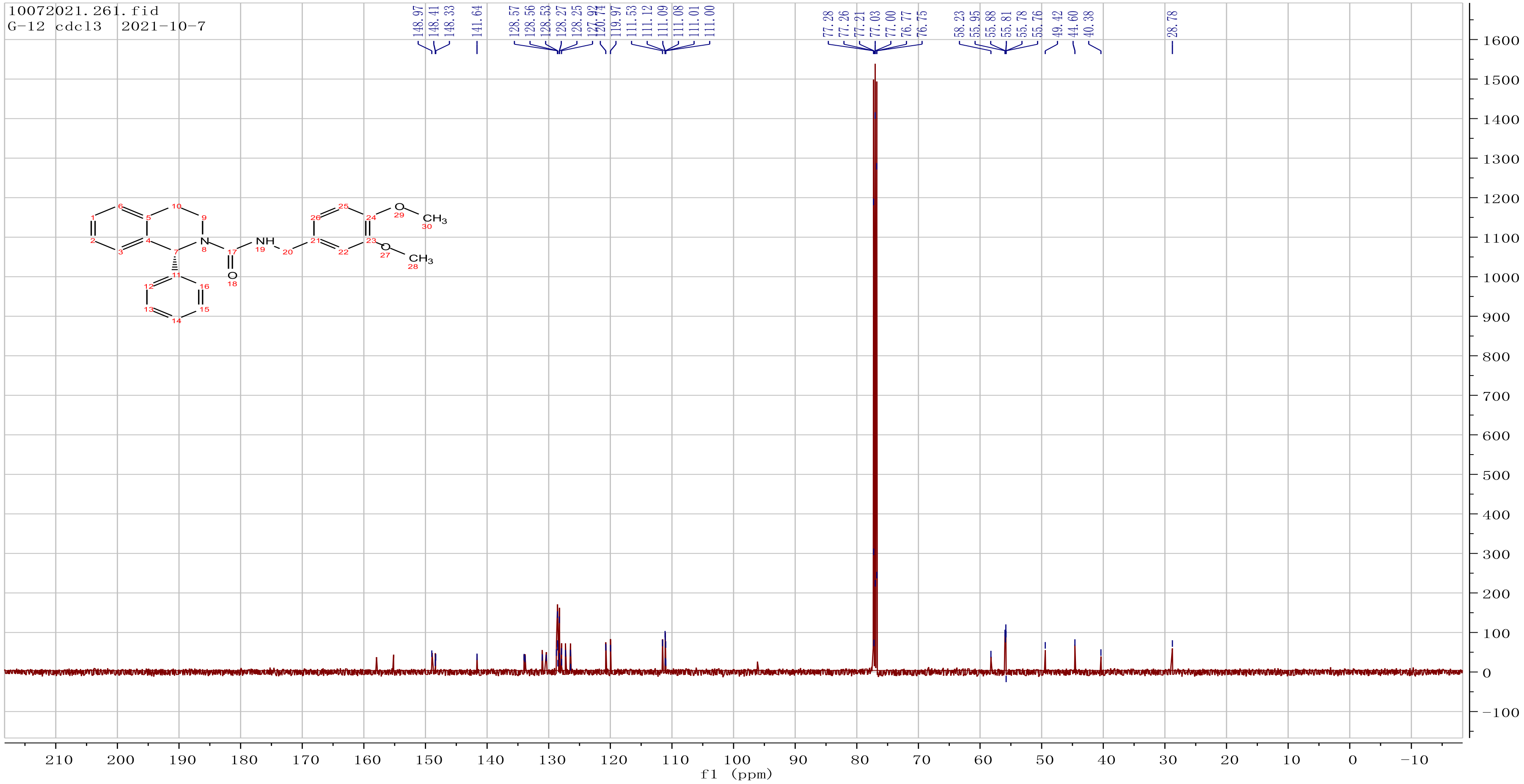

^{13}C-NMR Spectral of **2w**

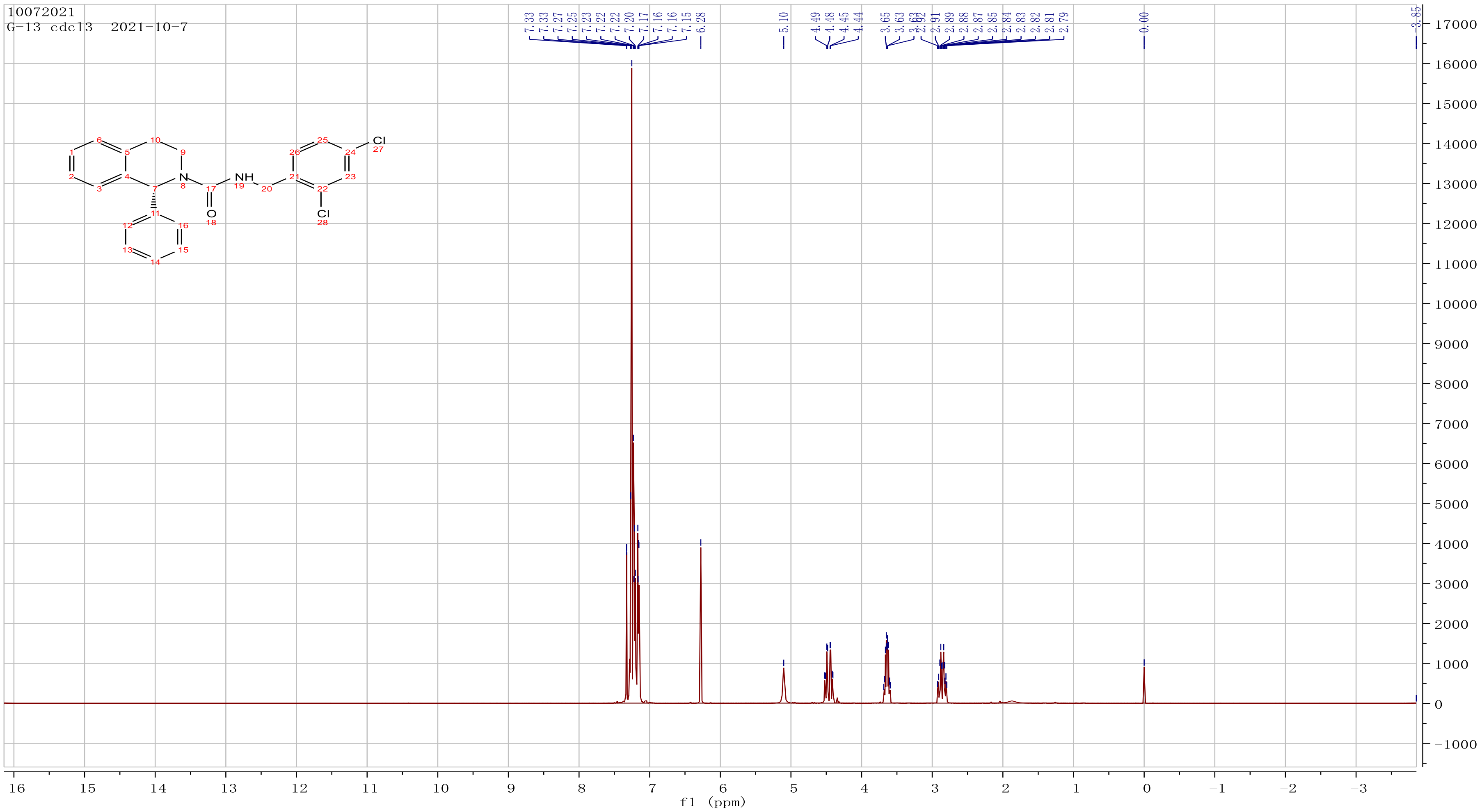

^{1}H-NMR Spectral of **2x**

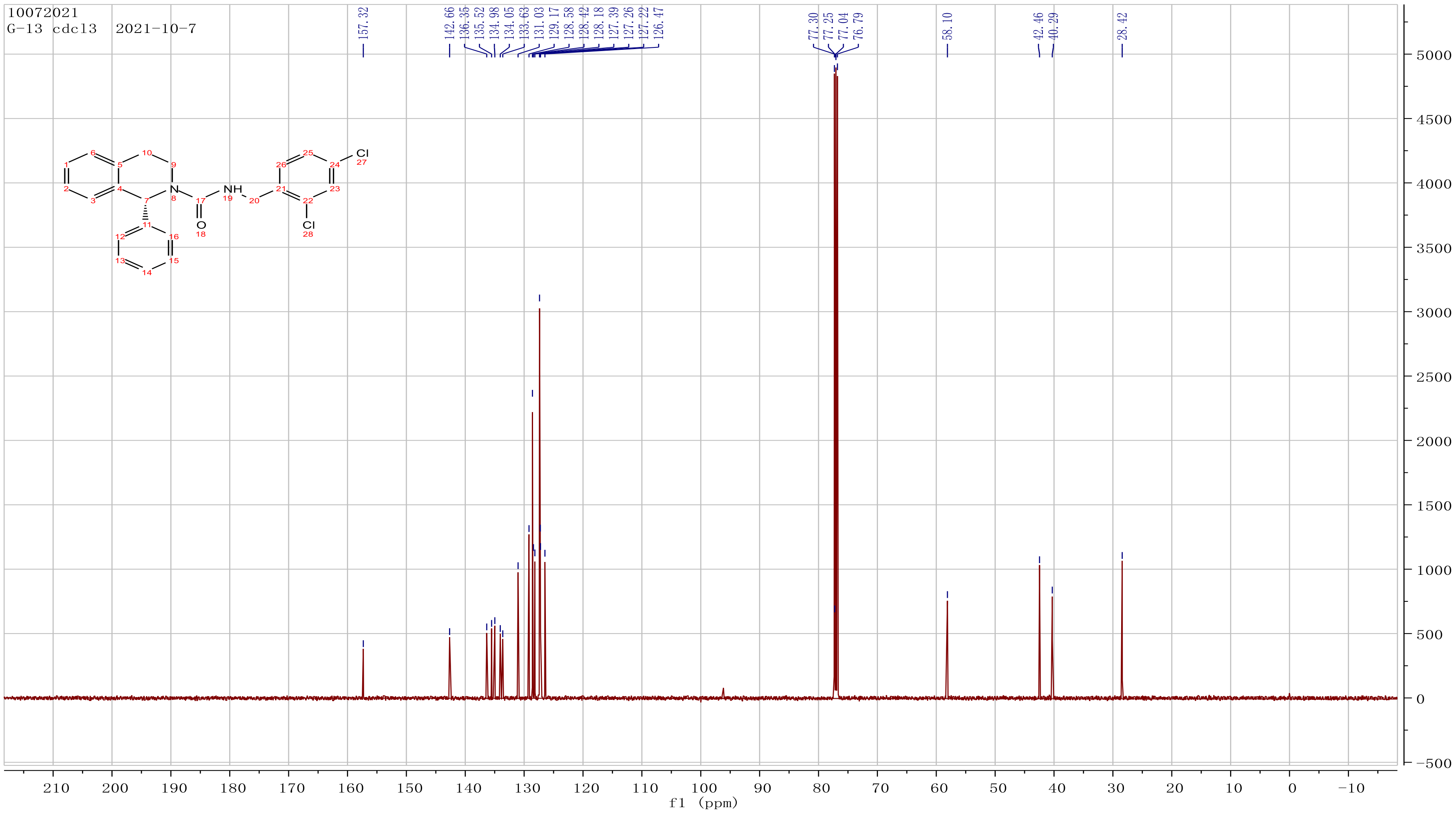

^{13}C-NMR Spectral of **2x**

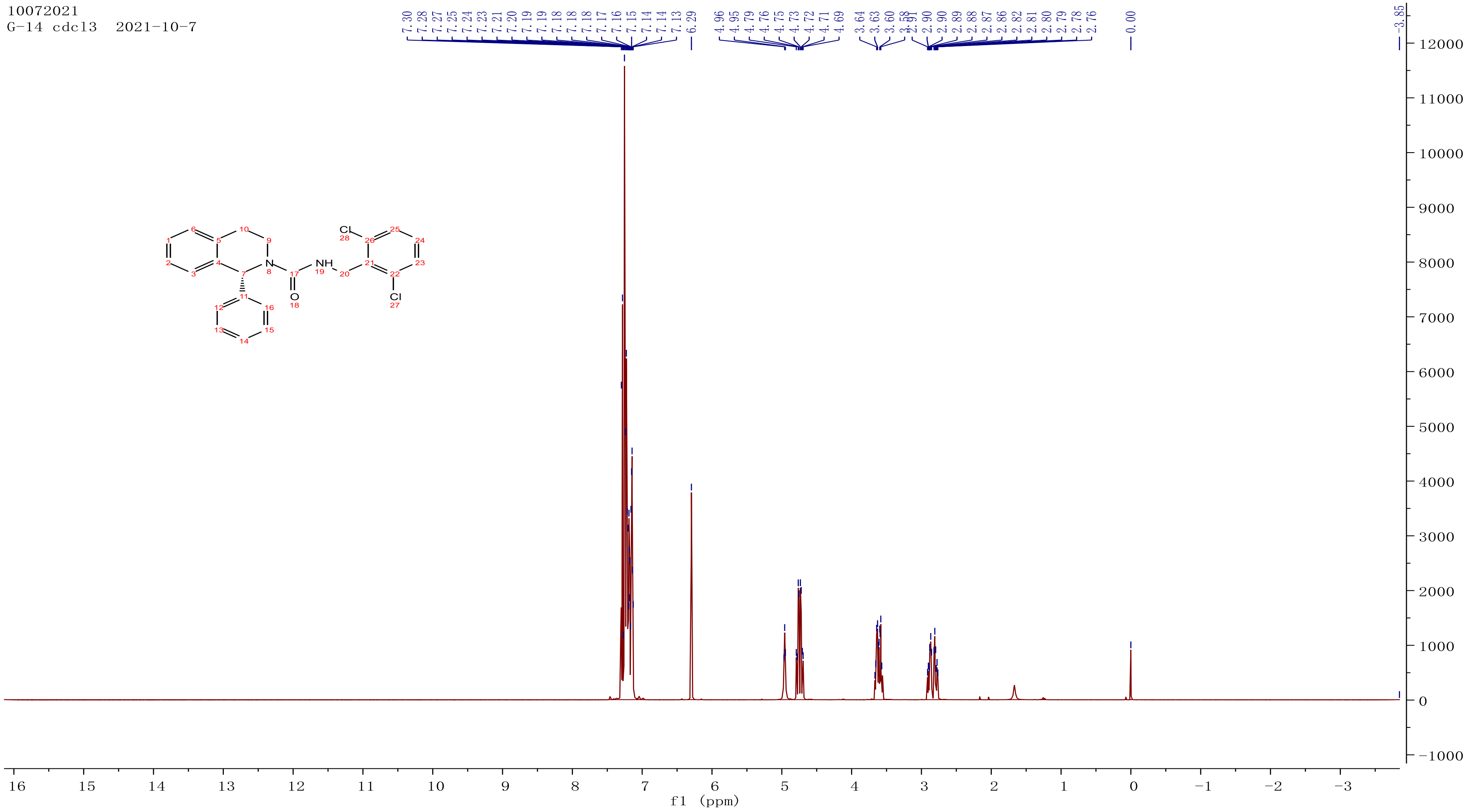

^{1}H-NMR Spectral of **2y**

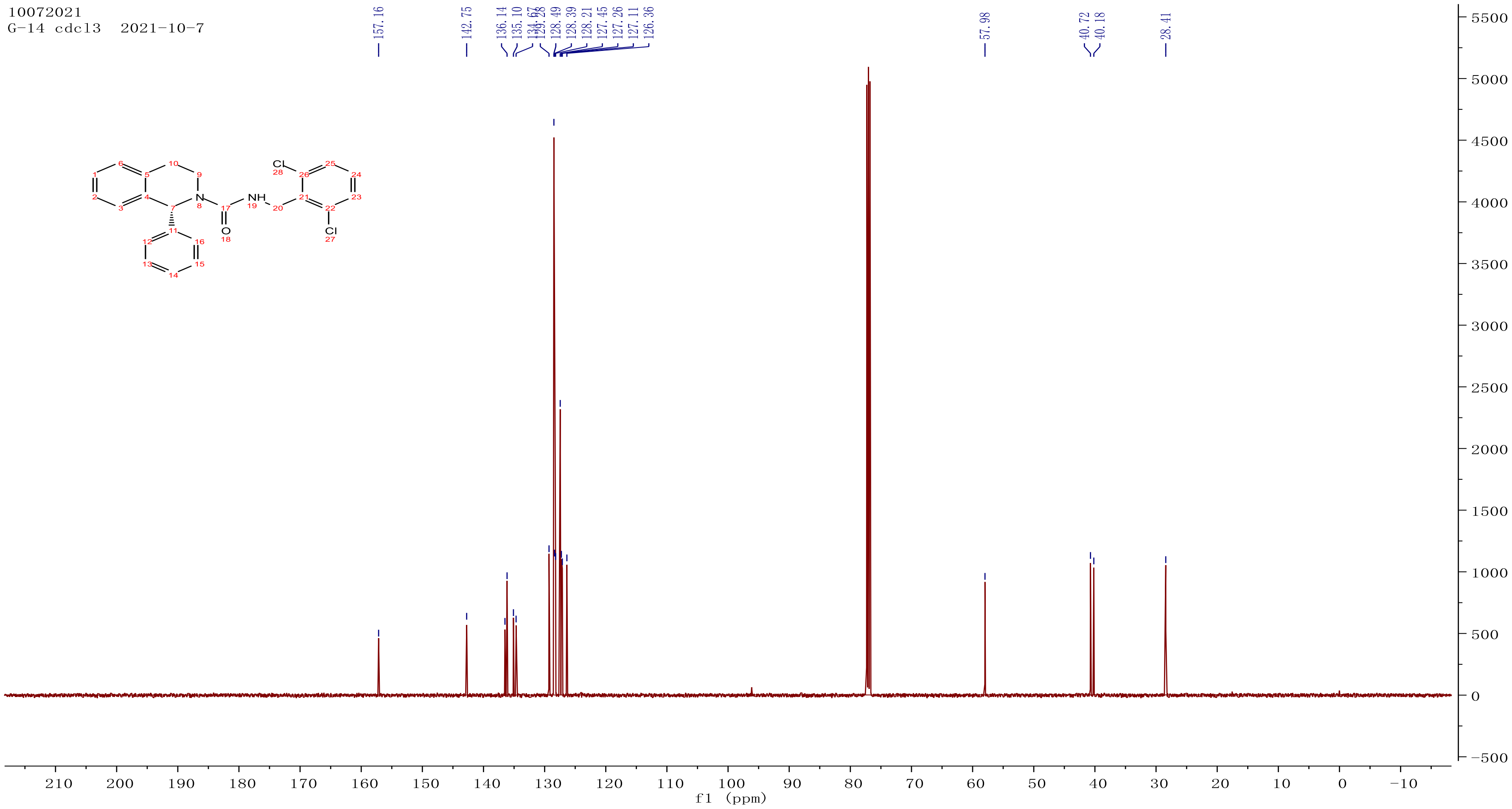

^{13}C-NMR Spectral of **2y**

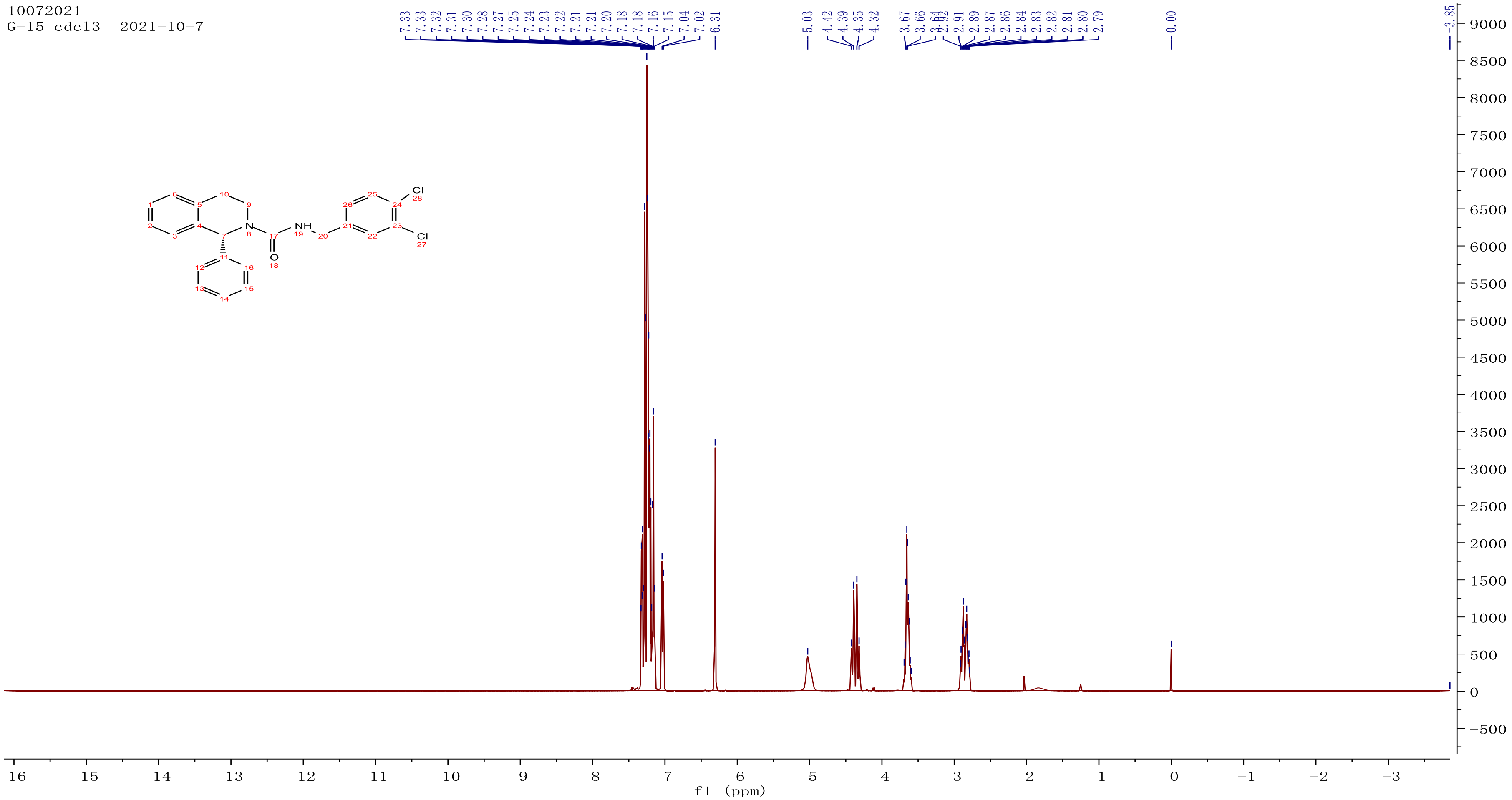

^{1}H-NMR Spectral of **2z**

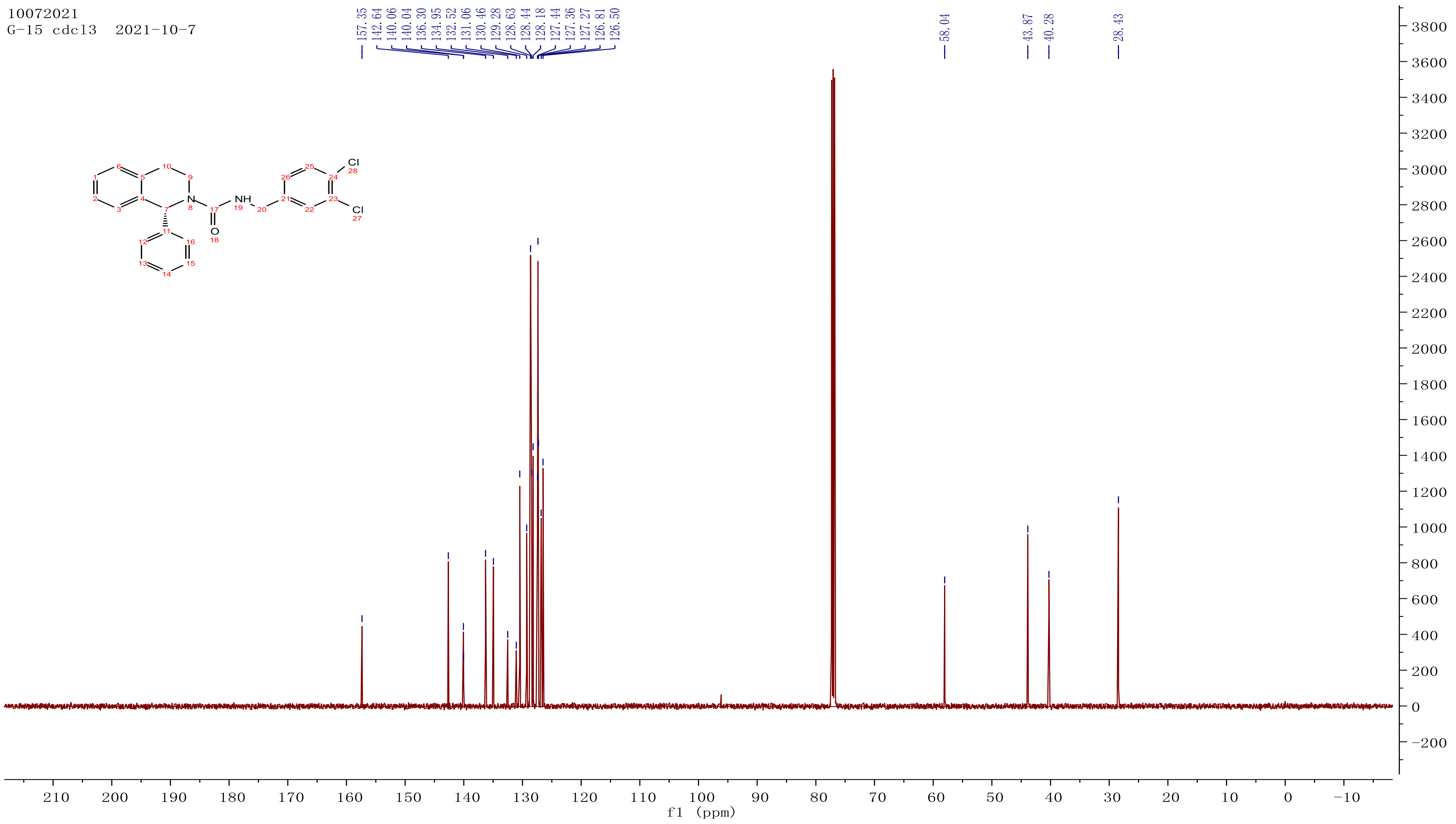

^{13}C-NMR Spectral of **2z**

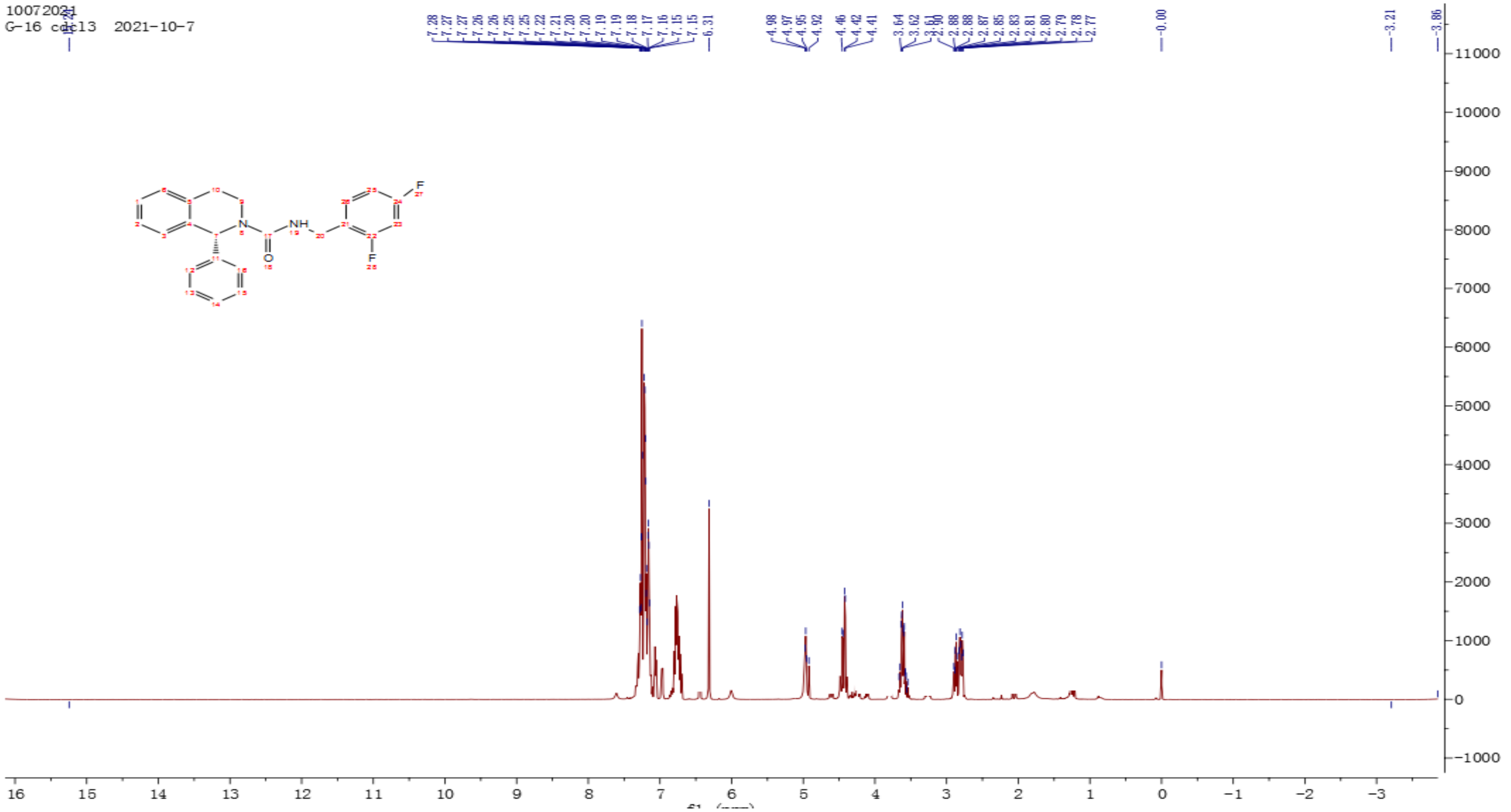

^{1}H-NMR Spectral of **2aa**

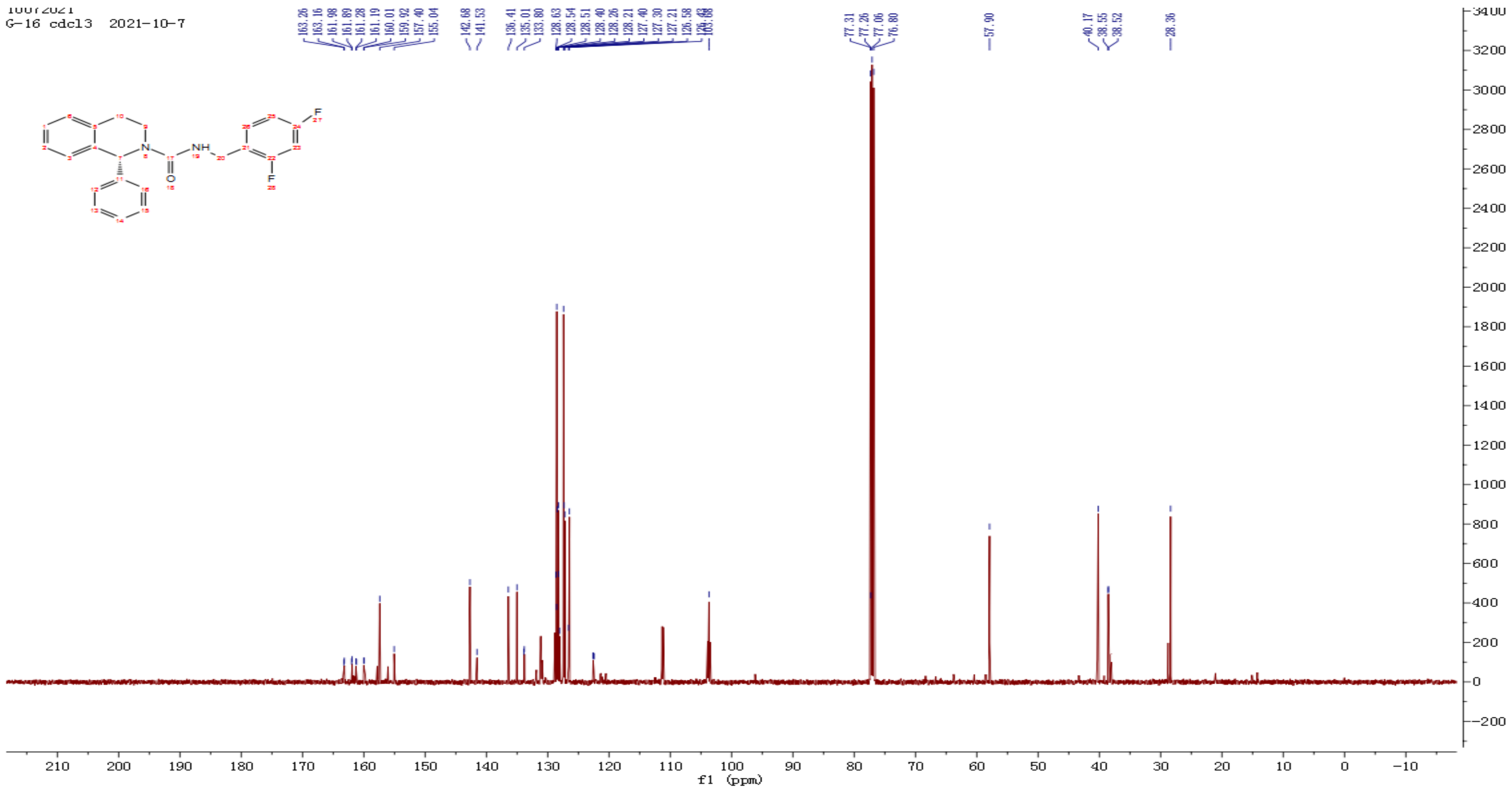

^{13}C-NMR Spectral of **2aa**

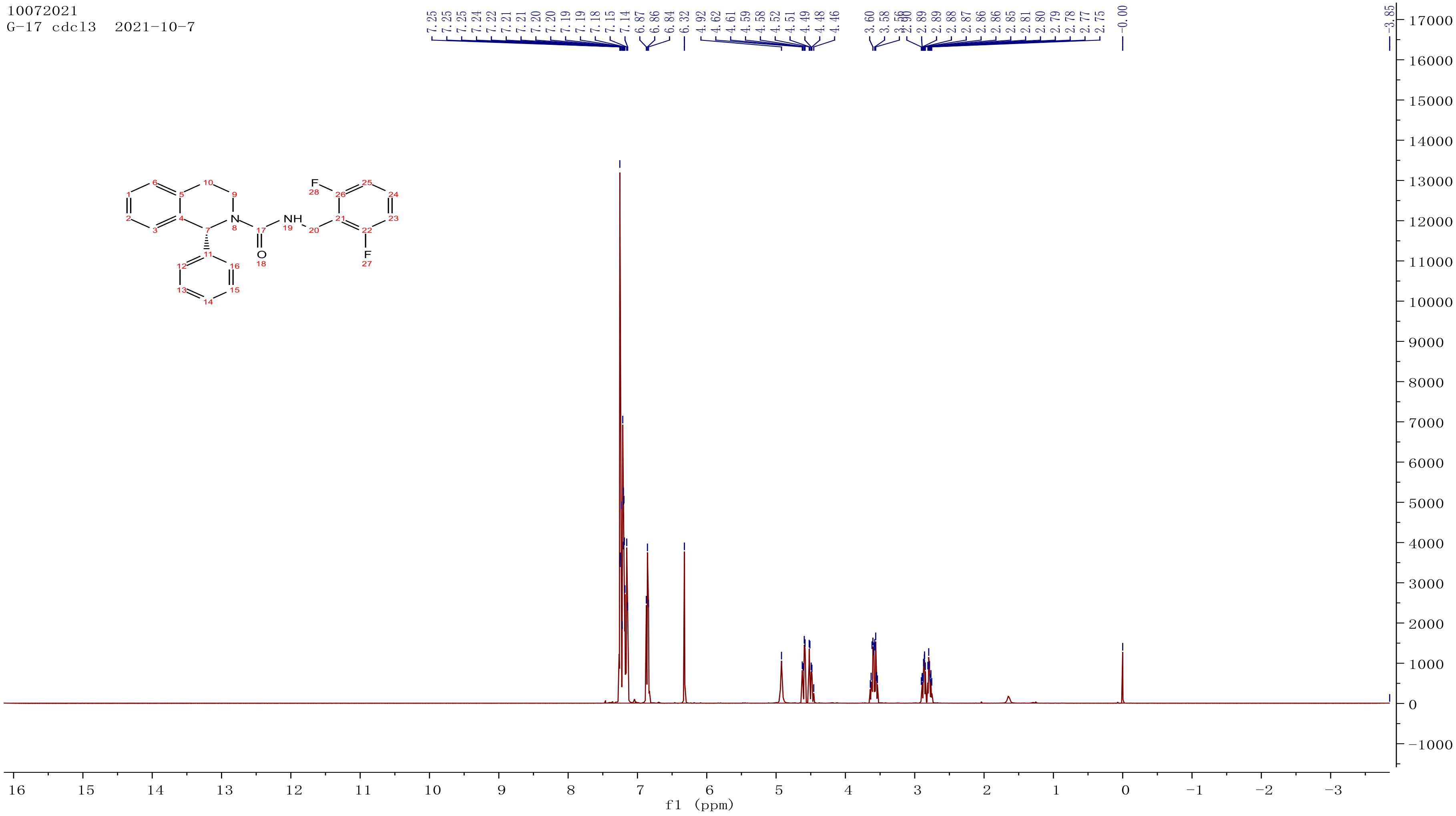

^{1}H-NMR Spectral of **2ab**

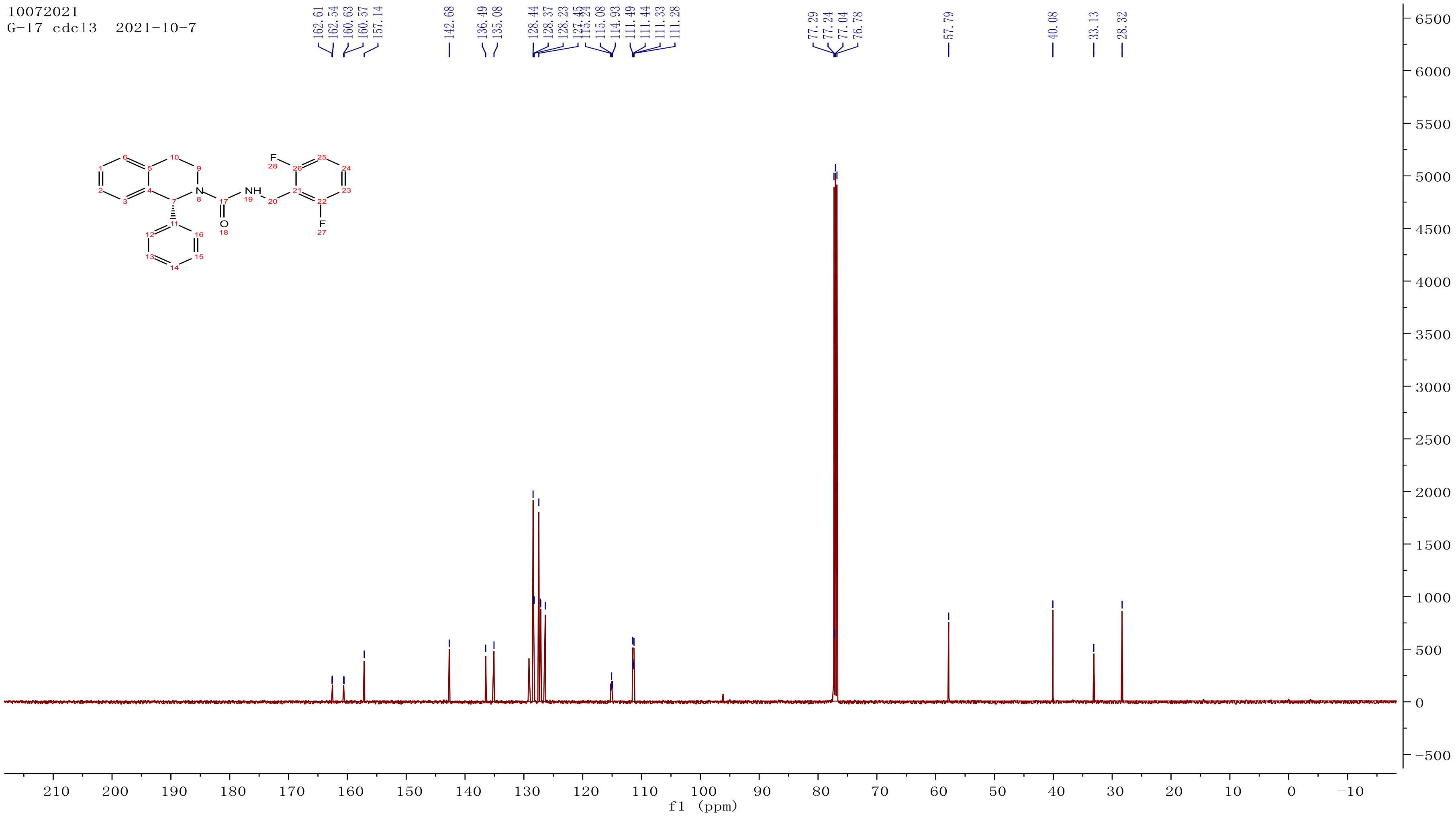

^{13}C-NMR Spectral of **2ab**

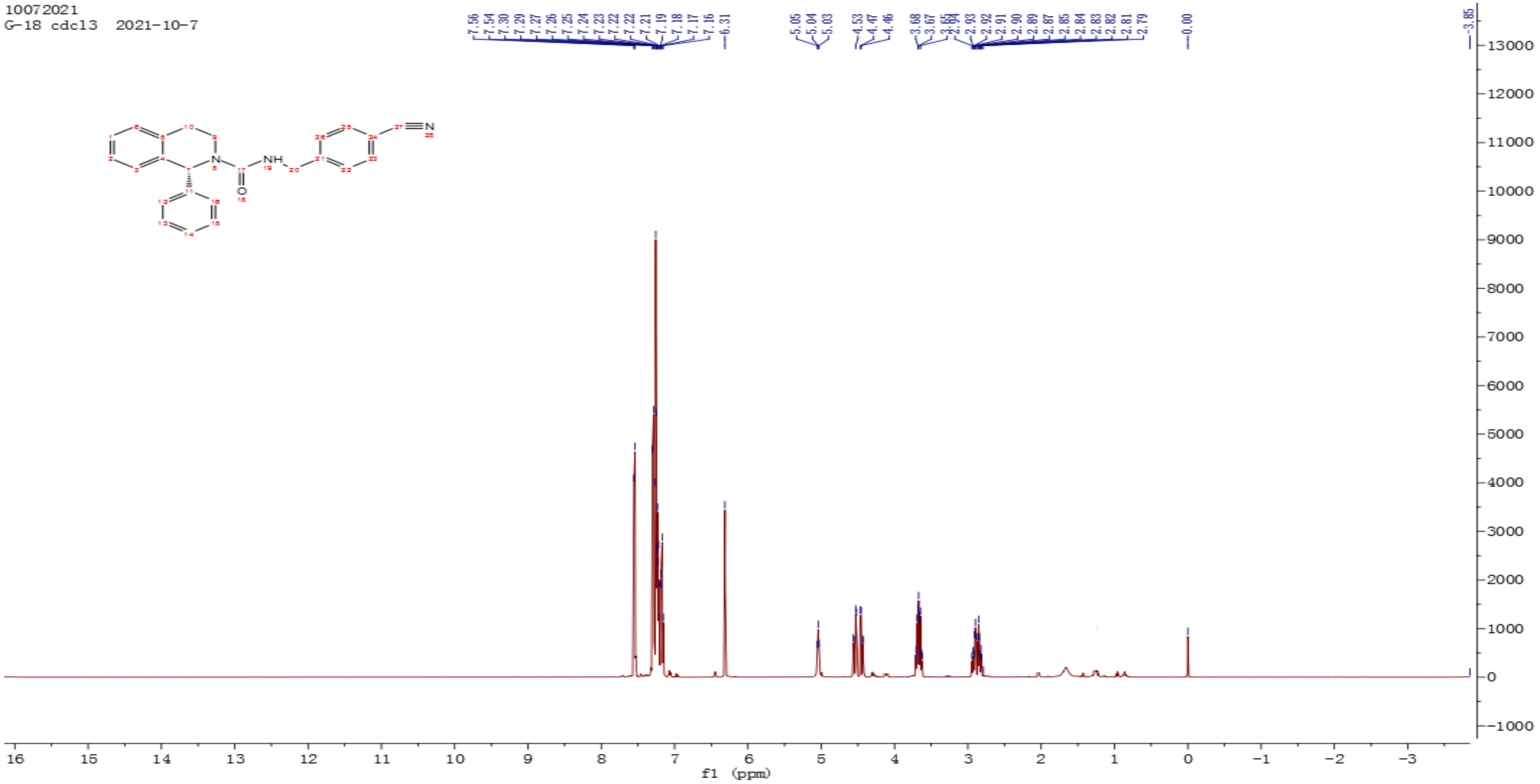

^{1}H-NMR Spectral of **2ac**

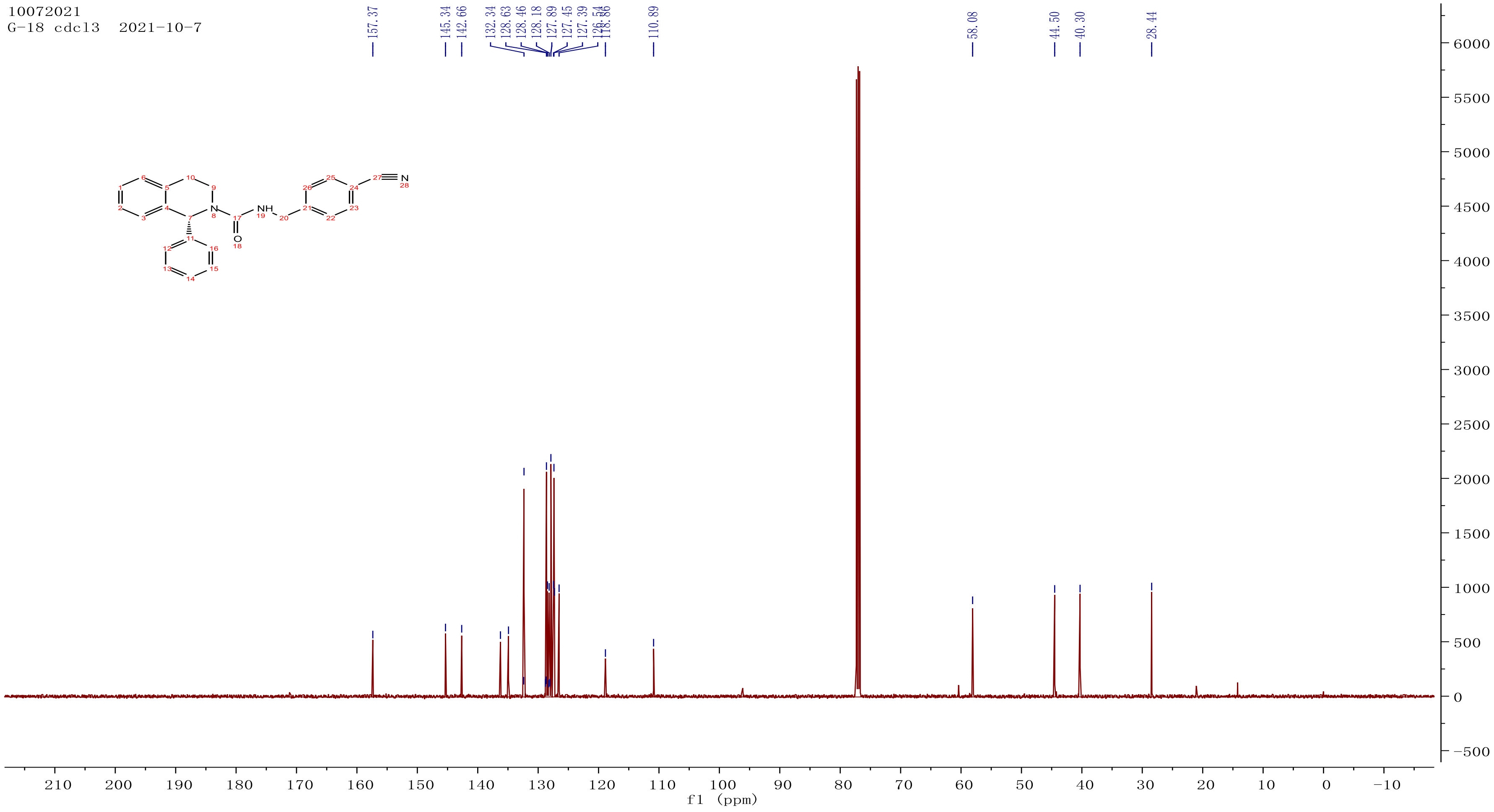

^{13}C-NMR Spectral of **2ac**

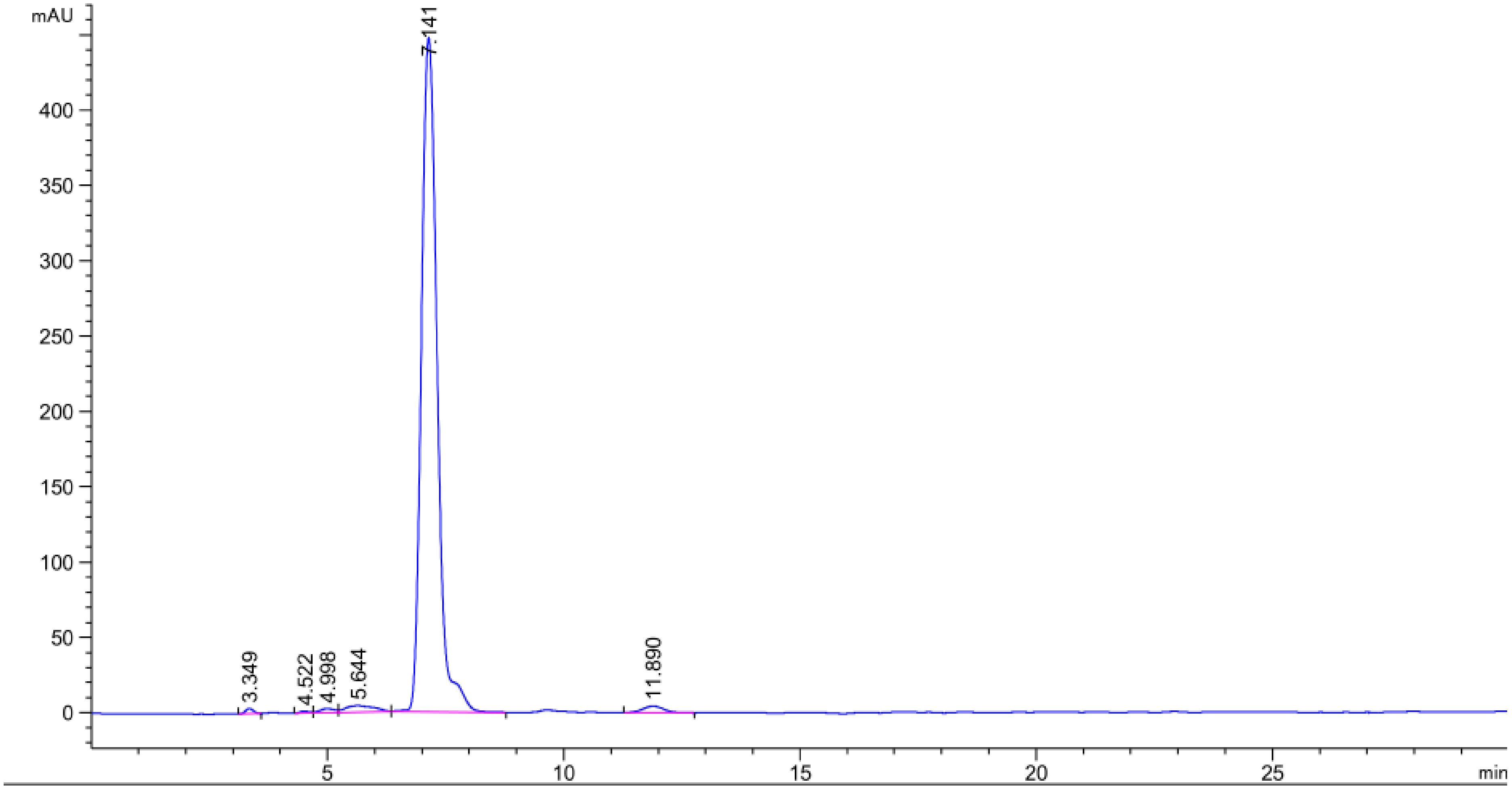

HPLC of compound 2d

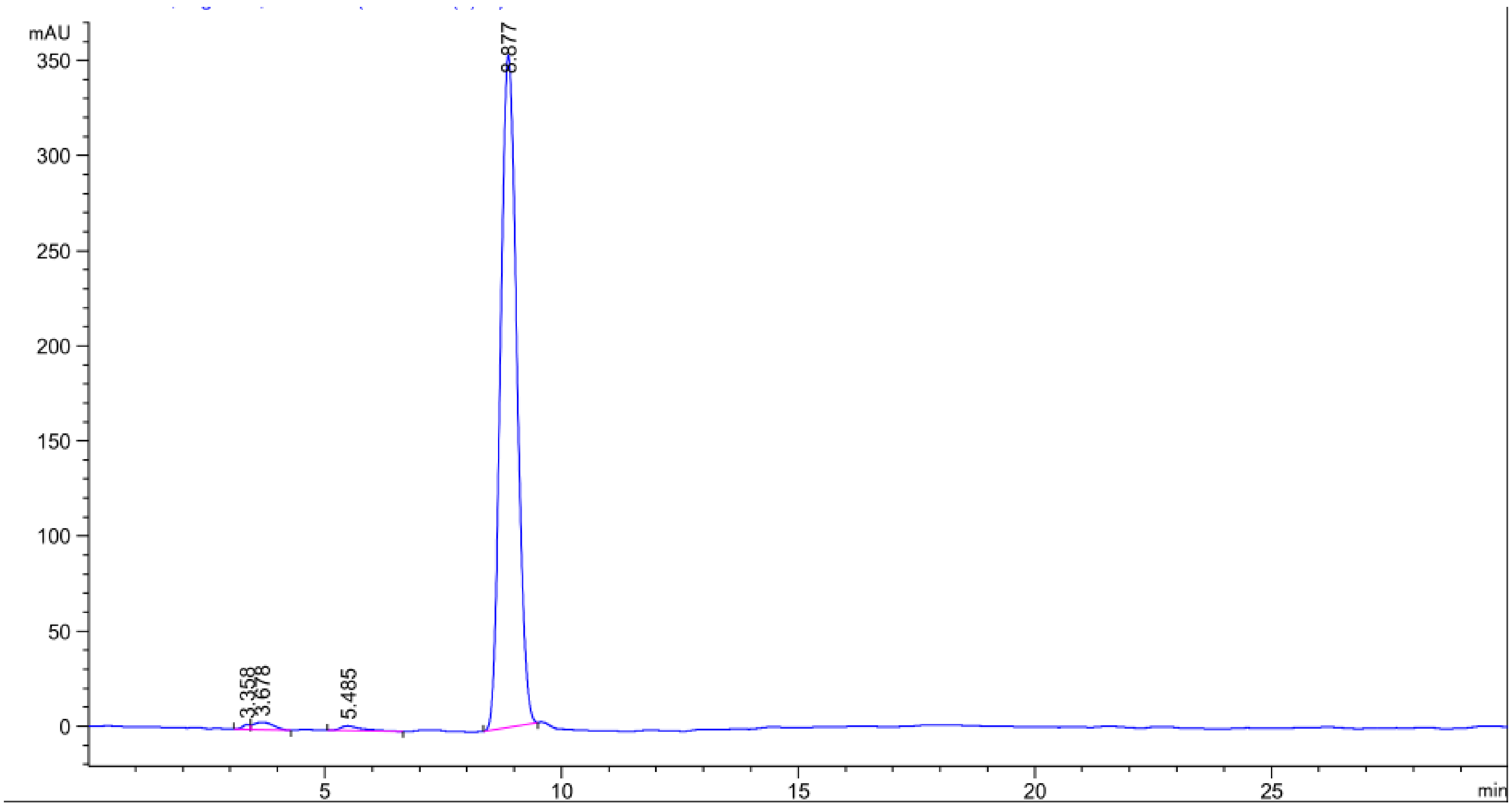

HPLC of compound 2i

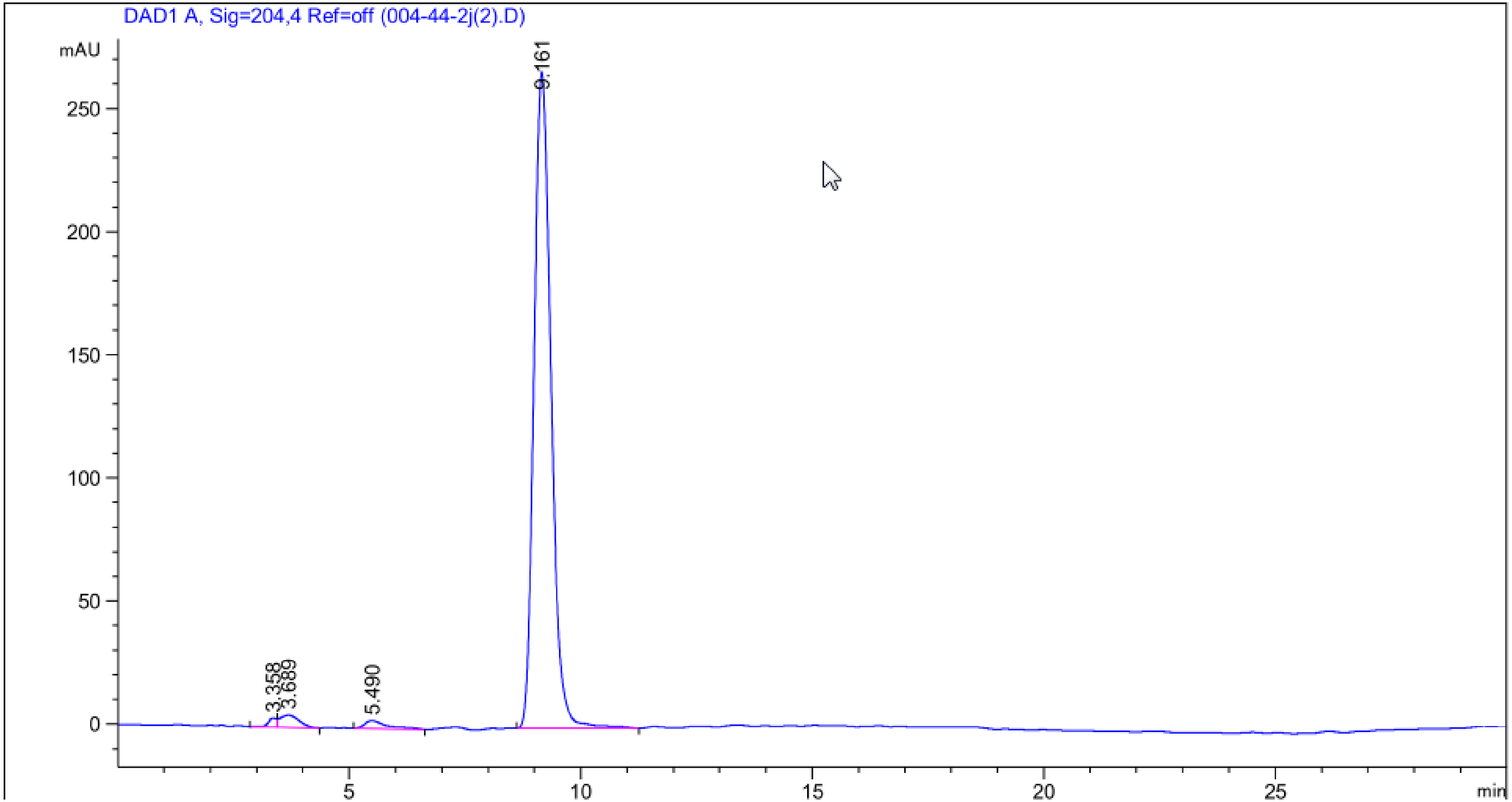

HPLC of compound 2j

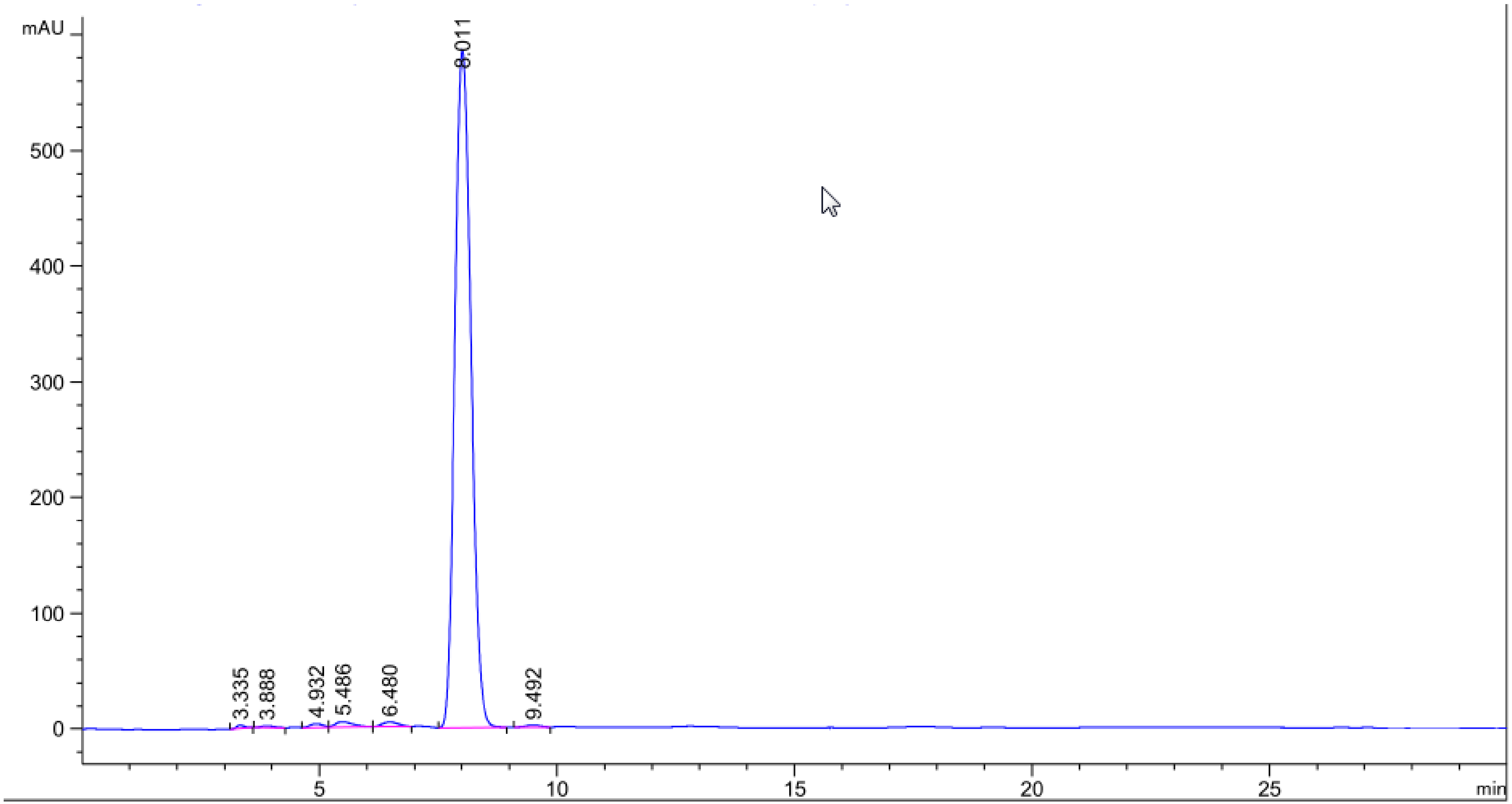

HPLC of compound 2p

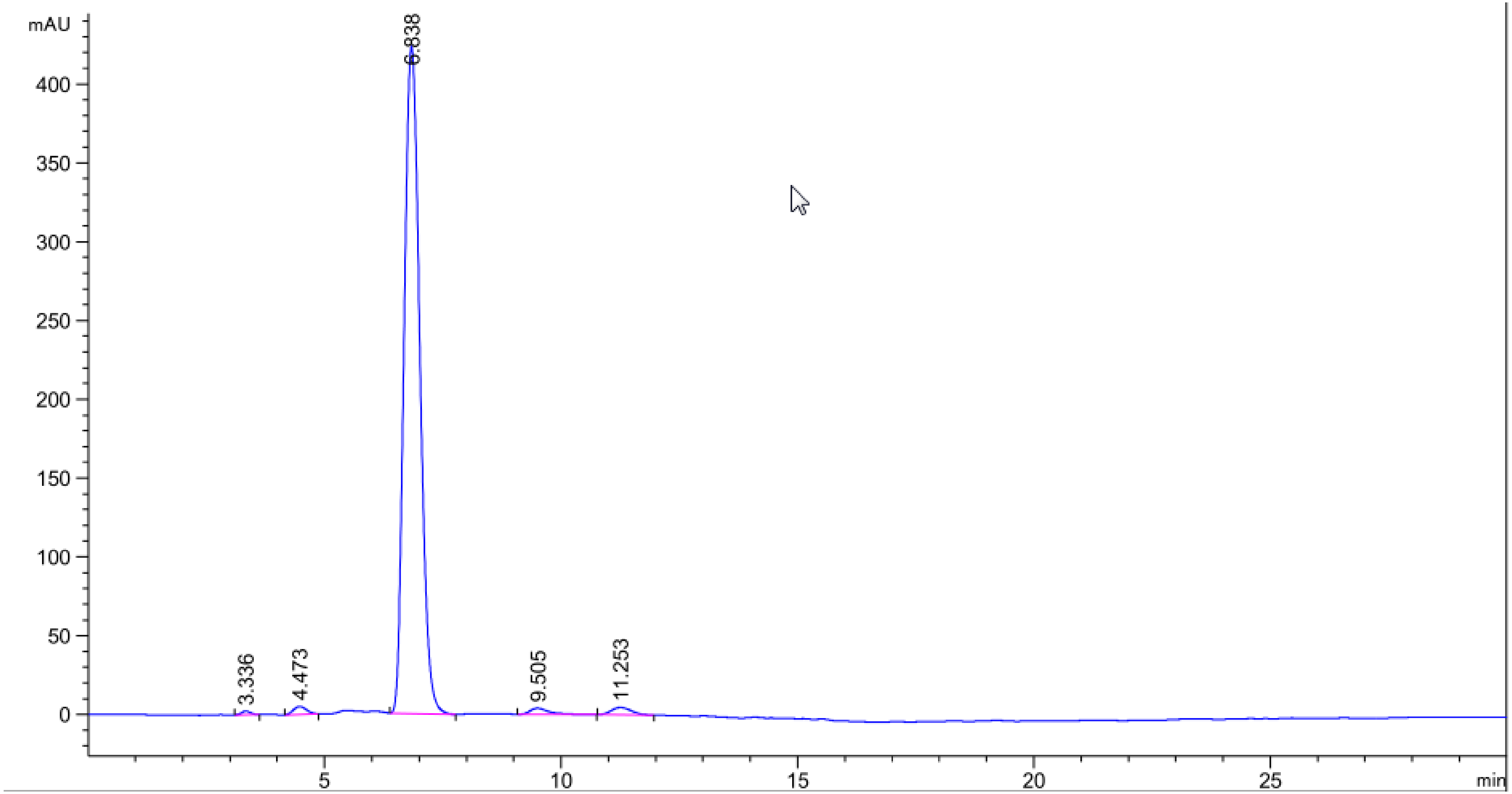

HPLC of compound 2t

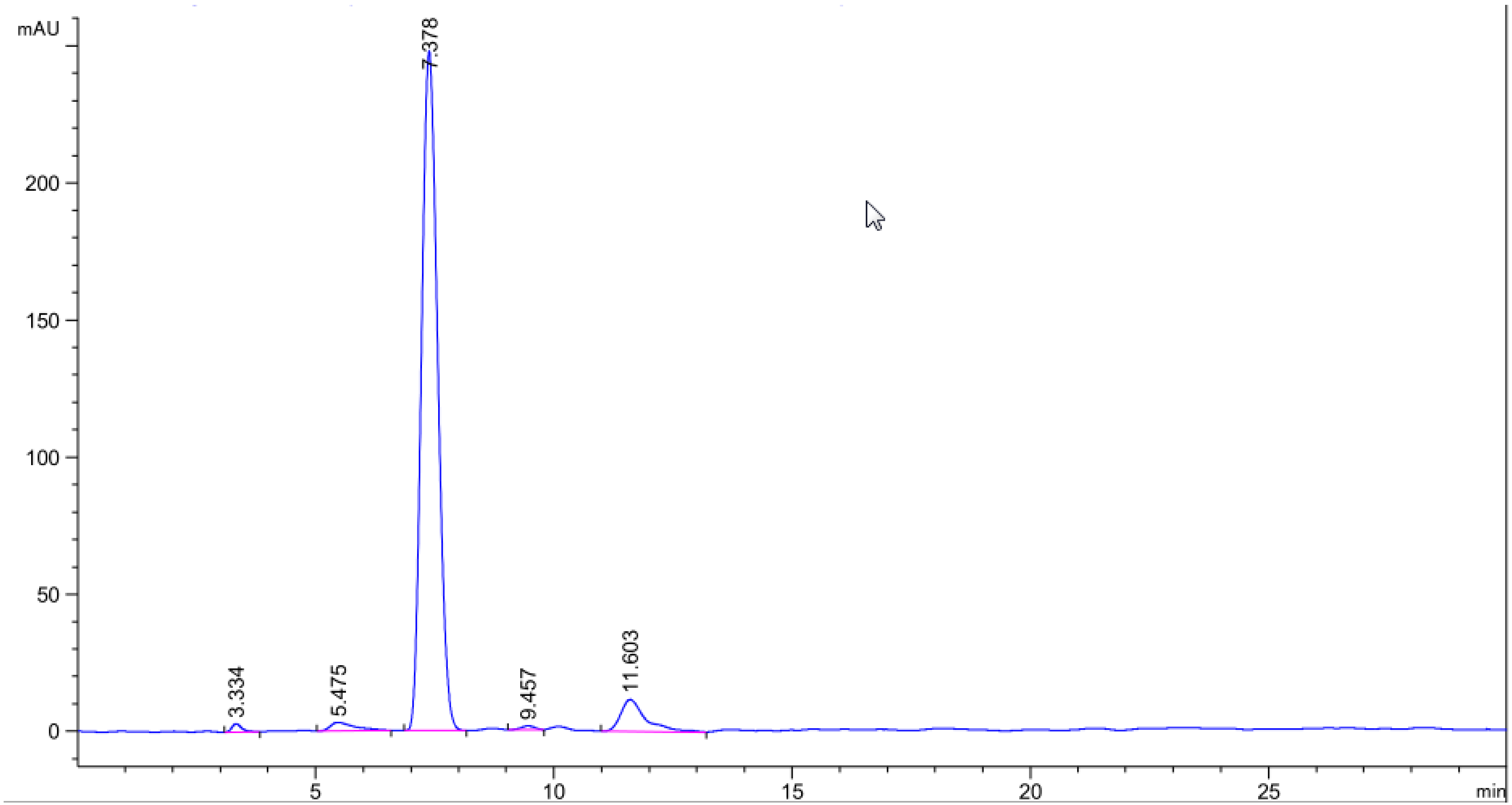

HPLC of compound 2v
